# Pyridine based dual binding site aromatase (CYP19A1) inhibitors†

**DOI:** 10.1039/d2md00352j

**Published:** 2023-01-03

**Authors:** Ahmed G. Eissa, Lauren E. Powell, Julia Gee, Paul A. Foster, Claire Simons

**Affiliations:** a School of Pharmacy & Pharmaceutical Sciences, Cardiff University King Edward VII Avenue Cardiff CF10 3NB UK simonsc@cardiff.ac.uk; b Institute of Metabolism & Systems Research (IMSR), University of Birmingham Birmingham B15 2TT UK; c Centre for Endocrinology, Diabetes, and Metabolism, Birmingham Health Partners Birmingham B15 2TT UK

## Abstract

Aromatase (CYP19A1) inhibitors are the mainstay therapeutics for the treatment of hormone dependant breast cancer, which accounts for approximately 70% of all breast cancer cases. However, increased resistance to the clinically used aromatase inhibitors, including letrozole and anastrazole, and off target effects, necessitates the development of aromatase inhibitors with improved drug profiles. The development of extended 4th generation pyridine based aromatase inhibitors with dual binding (haem and access channel) is therefore of interest and here we describe the design, synthesis and computational studies. Cytotoxicity and selectivity studies identified the pyridine derivative (4-bromophenyl)(6-(but-2-yn-1-yloxy)benzofuran-2-yl)(pyridin-3-yl)methanol (10c) as optimal with CYP19A1 IC_50_ 0.83 nM (*c.f.* letrozole IC_50_ 0.70 nM), and an excellent cytotoxicity and selectivity profile. Interestingly, computational studies for the 6-*O*-butynyloxy (10) and 6-*O*-pentynyloxy (11) derivatives identified an alternative access channel lined by Phe221, Trp224, Gln225 and Leu477, providing further insight into the potential binding mode and interactions of the non-steroidal aromatase inhibitors.

## Abstract 2

4th generation pyridine based dual site aromatase (CYP19A1) inhibitors were synthesised and evaluated for CYP19A1 inhibitory activity, cytotoxicity and CYP selectivity. Computational studies provided insight into the potential binding mode.

## Graphical abstract

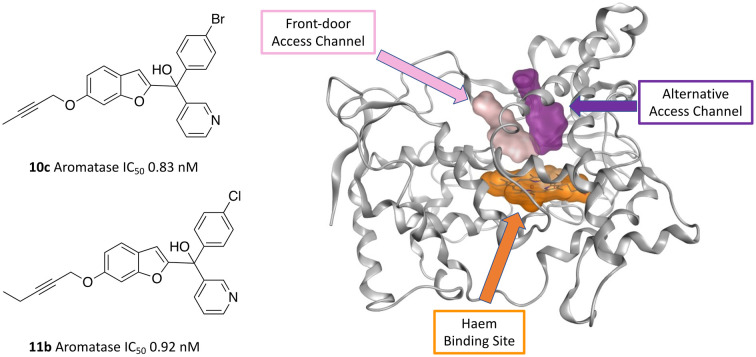

## Introduction

Aromatase inhibitors (AIs) have proved to be very successful clinically in the treatment and prophylaxis of hormone dependent breast cancer,^1^ which accounts for approximately 70% of all breast cancers.^2^ Although very effective, resistance to AIs in addition to the side effects associated with both hormone ablation and off target effects^3,4^ supports the further development of AIs that are as effective as the currently used clinical AIs but with improved selectivity to reduce unwanted side effects. Non-steroidal aromatase inhibitors (*e.g.* letrozole and anastrazole) bind to the enzyme active site through coordination of a heterocyclic nitrogen lone pair with the haem iron. Imidazole was reported to be more efficient in respect to the coordination potential followed by triazole then tetrazole, however, triazole compounds were found to be more selective.^5^ Pyridine nitrogen has availability of a lone pair with the potential to coordinate the haem iron, and the AI activity was found to fall between triazole and tetrazole moieties making pyridine an interesting heterocycle for the aromatase inhibition due to the increased size, which may lead to a closer interaction with the haem.^6^ Several studies have focused on the ability of pyridine compounds to bind to and inhibit the aromatase (CYP19A1) enzyme.^7–9^ In a previous study reported by us on benzofuran derivatives, the aromatase inhibitory activity was found to be directly proportional to the heterocyclic nitrogen basicity (p*K*_a_: imidazole, 14.5; triazole, 10; pyridine, 5.2; tetrazole, 4.8),^6^ however we also observed improved cytotoxicity profile (LC_50_/IC_50_ > 2000) of pyridine derivatives compared with triazole derivatives.^11^

**Fig. 1:**
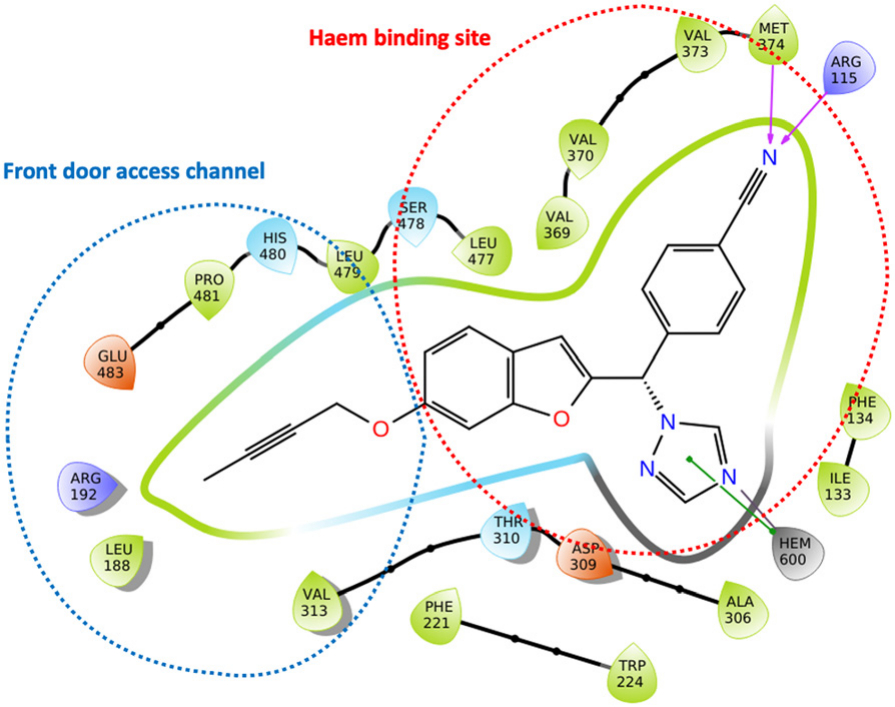
Lead extended triazole benzofuran AI^10^ with binding in the haem proximal site (red dotted circle) and front door access channel (blue dotted circle) gated by Arg192 and Glu483.

We have recently reported potent low nanomolar/picomolar extended 4th generation aromatase inhibitors based on a benzofuran-2-yl pharmacophore containing a triazole group as the azole required for haem binding.^10^ Inclusion of a long alkynyloxy chain at the 6-position of the benzofuran ring resulted in these AIs having dual CYP19A1 binding properties by binding in both the haem and the front door access channel sites (Fig. 1), which allows better fill of the enzyme and good selectivity profiles with respect to human CYP enzymes. Further development of these AIs was considered, maintaining the benzofuran pharmacophore and the most effective long alkynyloxy groups (but-2-ynyloxy and pent-2-ynyloxy), but replacing the triazole haem binding group with a pyridine to determine both CYP19A1 inhibitory activity, cytotoxicity and selectivity profiles for comparison with the respective triazole AIs.

**Fig. 2:**
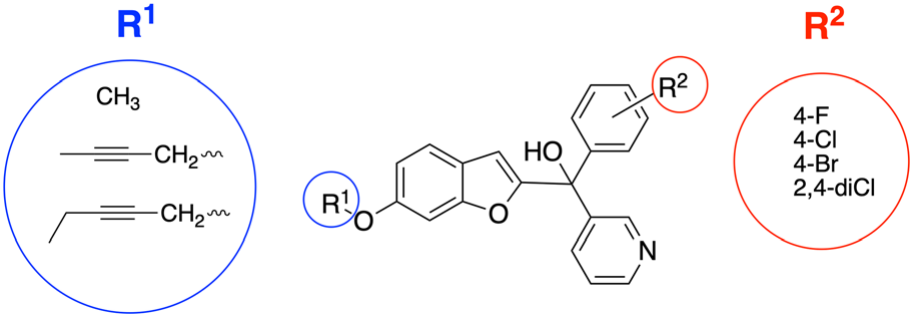
Designed pyridine benzofuran AIs with alkoxy substituents (R^1^) to explore access channel binding and phenyl substitutions (R^2^) to determine effect on enzyme inhibition.

A series of pyridine-based benzofuran compounds was designed based on the parent scaffold previously reported by our research group (Fig. 2, R^1^ = OCH_3_, R^2^ = 4-F or 4-Cl), which used a human placental microsomal assay to determine aromatase inhibition.^11^ The halides were the most promising derivatives (IC_50_, 44 and 49 nM respectively^11^), therefore the first modification was to prepare and evaluate the 4-bromo and 2,4-dichloro as well as the parent 4-fluoro and 4-chlorophenyl benzofuran pyridines for evaluation in the placental choriocarcinoma JEG-3 cell aromatase assay, producing four different derivatives to provide better understanding of the SAR. The second modification was achieved by changing the methoxy group on the benzofuran ring with longer chain substituents, namely but-2-ynyloxy and pent-2-ynyloxy, to investigate the binding potential in the front door access channel of the enzyme to provide dual binding aromatase inhibitors.

## Results and discussion

### Chemistry

**Scheme 1:**
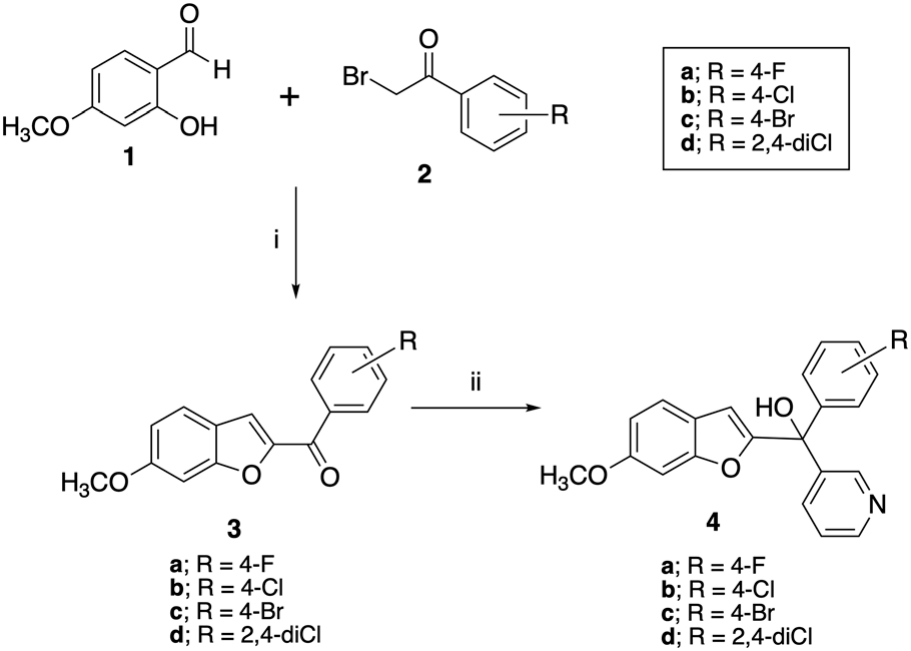
Synthesis of methoxy substituted compounds (4). Reagents and conditions: (i) K_2_CO_3_, CH_3_CN, 70 °C, 3 h, 74–96%; (ii) pyridine 3-magnesium bromide, THF, 70 °C, 16 h, 25–92%.

A two-step synthetic pathway as originally reported was used to prepare the required parent compounds (4) with the 6-methoxy substitution on the benzofuran ring^11^ (Scheme 1). The first step involved a Rap–Stoermer condensation reaction between 2-hydroxy-4-methoxybenzaldehyde (1) and the substituted bromoacetophenones (2) to form the benzofuran ketone derivatives (3), which were then used in a Grignard reaction with pyridine 3-magnesium bromide, prepared *in situ*, to produce the final compounds (4). The yields varied from 25% to 92% (Table 1), which can be attributed to the extent of formation of the Grignard reagent, or the time allowed for the reaction.

**Table 1:** Yields and characteristic ^13^C NMR C-1 signal

Compound	R	Yield (%)	C-1 signal (ppm)
4a	4-F	83	75.86 (B)
4b	4-Cl	92	76.51 (A)
4c	4-Br	25	75.92 (B)
4d	2,4-DiCl	32	77.18 (A)
10a	4-F	80	84.06 (A)
10b	4-Cl	94	84.08 (A)
10c	4-Br	70	84.07 (A)
10d	2,4-DiCl	34	84.09 (A)
11a	4-F	20	89.83 (A)
11b	4-Cl	91	89.87 (A)
11c	4-Br	50	89.87 (A)
11d	2,4-DiCl	15	89.89 (A)

**Scheme 2:**
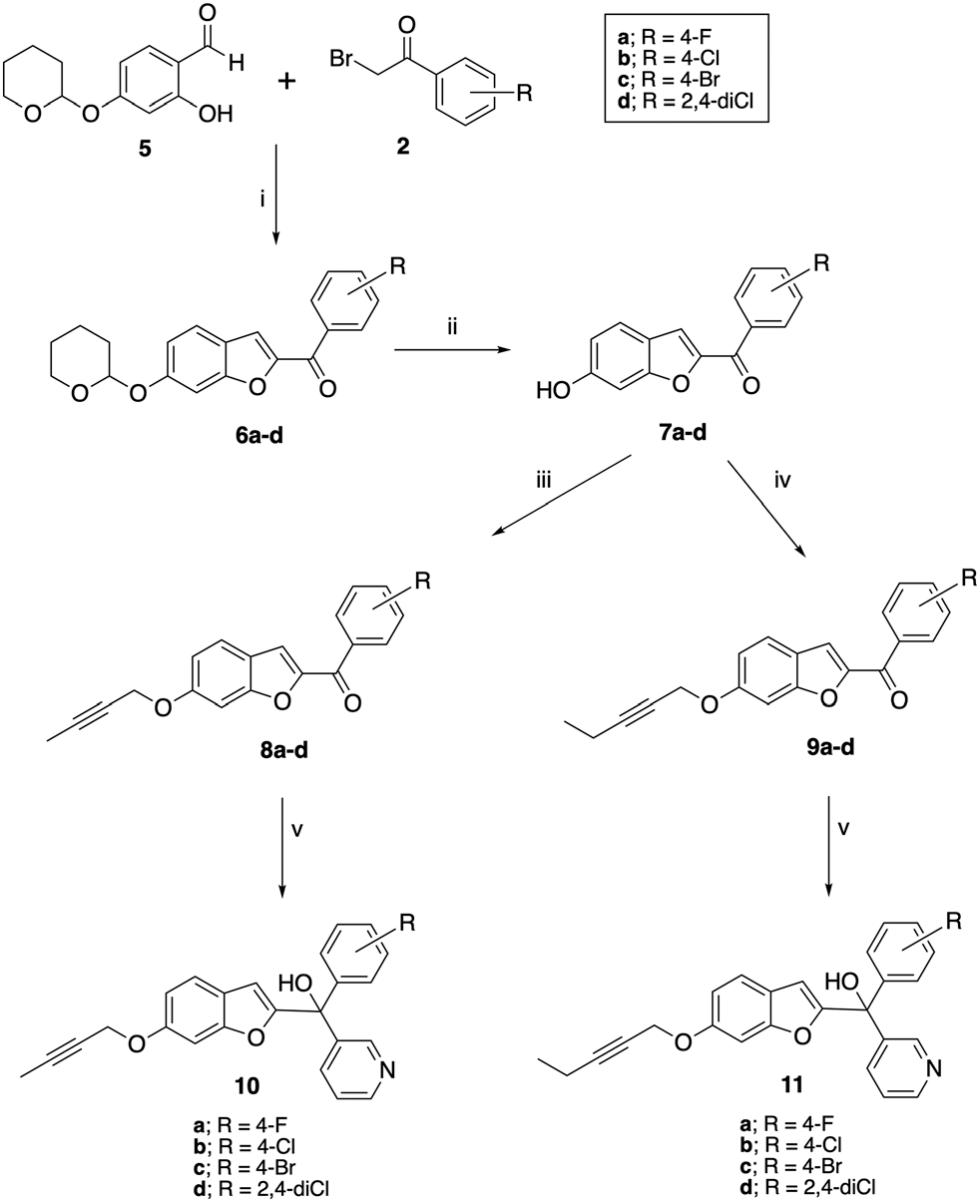
Synthesis of 6-*O*-but-2-ynyl (10) and 6-*O*-pent-2-ynyl (11) compounds. Reagents and conditions: (i) K_2_CO_3_, CH_3_CN, 70 °C, 3 h, 52–86%; (ii) HCl, dioxane, rt, 1 h 64–71%; (iii) K_2_CO_3_, CH_3_CN, 40 °C, 1 h, then 1-bromobut-2-yne, rt, 16 h, 34–86%; (iv) K_2_CO_3_, CH_3_CN, 40 °C, 1 h, then 1-bromopent-2-yne, rt, 16 h, 35–76%; (v) pyridine 3-magnesium bromide (prepared *in situ*), THF, 70 °C, 16 h, 10 34–90%, 11 15–91%.

Preparation of the longer chain substituted compounds, 10 and 11, required a different approach with a four-step synthetic pathway (Scheme 2). The pyran protected ketones (6) were prepared as previously described^10,11^ from pyran protected salicylaldehyde (5) and the substituted bromoacetophenones (2), followed by removal of the pyran group under acidic conditions to give the phenols (7). The phenol derivatives were then deprotonated using K_2_CO_3_ and treated with either 1-bromobut-2-yne or 1-bromopent-2-yne to give the corresponding alkyne ethers (8 and 9). Grignard reaction of 8 and 9 with pyridine 3-magnesium bromide then provided the final pyridyl products 10 in yields of 34–90% and 11 in yields of 15–91% (Table 1).

The pyridine products (4, 10 and 11) were confirmed by the pyridine signals in the aromatic region of the ^1^H and ^13^C NMR, a broad singlet of the tertiary OH group in the ^1^H NMR and by the loss of the carbonyl signal ∼*δ* 182–183 observed in the precursor (3, 8 and 9). The distinct new C-1 quaternary carbon was observed in ^13^C NMR at *δ* 75.9–77.2 for methoxy derivatives 4, ∼*δ* 84.1 for the but-2-ynyl derivatives (10) and ∼*δ* 89.9 for the pent-2-ynyl derivatives (11) (Table 1).

### Aromatase (CYP19A1) inhibition

**Fig. 3:**
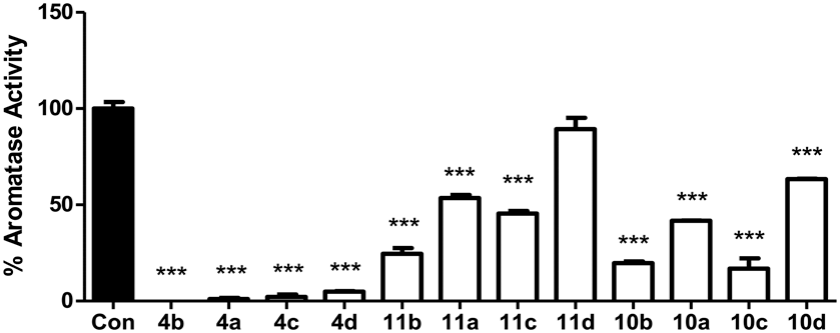
Aromatase activity assay at single concentration (10 nM) for compounds 4, 10 and 11. Stats are one-way ANOVA followed by a Tukey's multiple comparison test comparing all compounds against control. Data represents *n* = 3 technical replicates ± SEM. *** *p* < 0.001 compared to control.

The twelve final compounds (4a–d, 10a–d, 11a–d) were tested for their aromatase inhibitory activity using a modified tritiated water assay previously reported^12^ at a single concentration (10 nM). Briefly, placental choriocarcinoma JEG-3 cells, known to have high aromatase activity, were grown to approximately 80% confluence in six-well culture plates. Once established, cells were treated with androst-4-ene-3,17-dione[1β-^3^H] as aromatase substrate. Aromatase activity was measured in the absence and presence of inhibitors (Fig. 3).

From the initial screen at 10 nM concentration, eight of the 12 compounds were progressed to IC_50_ determination using a range of concentrations (0.001–10 nM). Aromatase activity results were determined as a concentration of product formed per mg of protein per hour. Each data point was measured in triplicates and the error in the IC_50_ calculations represented as 95% confidence interval (Table 2).

**Table 2:** Aromatase (CYP19A1) inhibitory activity of 6-OMe (4), 6-*O*-but-2-yne (10) and 6-*O*-pent-2-yne (11) benzofuran pyridyl derivatives

Compound	R	CYP19A1 IC_50_ (nM)	95% confidence interval (nM)
*6-OCH*_*3*_			
4a	4-F	0.74	0.598–0.925
4b	4-Cl	0.46	0.375–0.567
4c	4-Br	0.40	0.352–0.456
4d	2,4-diCl	1.90	1.527–2.398
*6-O-but-2-yne*			
10a	4-F	∼10	—
10b	4-Cl	1.05	0.763–1.446
10c	4-Br	0.83	0.665–1.038
10d	2,4-diCl	>10	—
*6-O-pent-2-yne*			
11a	4-F	∼10	—
11b	4-Cl	0.92	0.744–1.133
11c	4-Br	4.9	4.093–5.971
11d	2,4-DiCl	>10	—
Letrozole		0.70	0.556–0.883

The methoxy derivatives (4) were more potent (IC_50_ 0.4–1.90 nM) than the respective extended compounds (10 and 11). However, for the 6-*O*-but-2-yne derivatives the 4-Cl (10b IC_50_ 1.05 nM), and 4-Br (10c IC_50_ 0.83 nM) were comparable with the standard, letrozole (IC_50_ 0.70 nM), and for the 6-*O*-pent-2-yne derivatives the 4-Cl (11b IC_50_ 0.92 nM) was also comparable with letrozole. The least favourable substitution was 2,4-dichloro (10d and 11d, IC_50_ > 10 nM) followed by 4-F (10a and 11a, IC_50_ ∼ 10 nM), which mirrored the trend observed for the simpler methoxy derivatives (4a, IC_50_ 0.94 nM; 4d, IC_50_ 1.90 nM).

### Cytotoxicity

Compounds 4a–d, 10b–c and 11b–c were tested at 1 μM over 48 hours along with doxorubicin as positive control by BrdU proliferation assay to evaluate the cytotoxicity against non-oestrogen dependent breast cancer cells (MDA-MB-231), oestrogen-dependent breast cancer cells (MCF-7), and non-cancerous breast epithelial cells (MCF-10A).

**Fig. 4:**
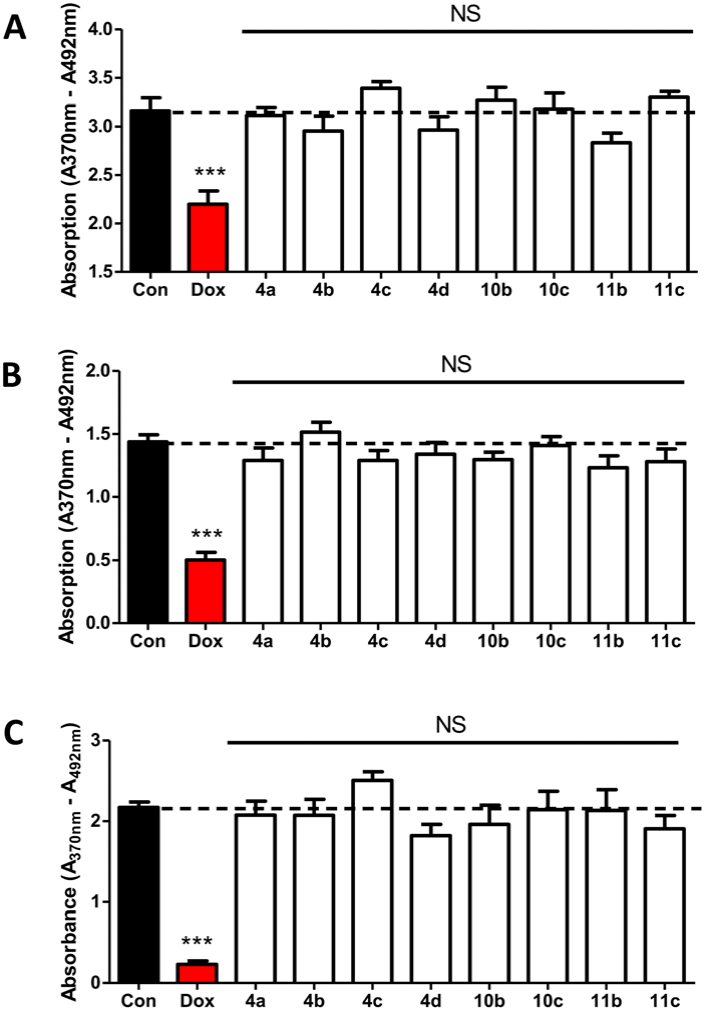
Cytotoxicity of pyridine compounds against (A) MDA-MB-231 (B) MCF-7 and (C) MCF-10A growth tested at 1 μM for 48 hours treatment followed by BrdU proliferation assay. Stats are one-way ANOVA followed by a Tukey's multiple comparison test comparing all compounds against control doxorubicin (1 μM). Data represents (A) *n* = 6 and (B and C) *n* = 5 technical replicates ± SEM. *** *p* < 0.001 compared to control. NS – non-significant compared to control.

Statistics using one-way ANOVA followed by a Tukey's Multiple Comparison test comparing all compounds against control showed no significant difference between the tested compounds and the negative control indicating that the compounds had no impact on MDA-MB-231 (Fig. 4A), MCF-7 (Fig. 4B) or MCF-10A (Fig. 4C) growth. These results are indicative of negligible off-target effects.

### Selectivity

The most active extended compound, (4-bromophenyl)(6-(but-2-yn-1-yloxy)benzofuran-2-yl)(pyridin-3-yl)methanol (10c), was tested for inhibitory activity against a CYP panel (1A2, 2C9, 2C19, 2D6 and 3A4) by Cyprotex Discovery Limited using a human liver microsomal assay with a CYP isoform specific probe substrate.^13^ Compound 10c displayed excellent selectivity for CYP19A1 compared with CYPs 1A2 and 2D6 (>30 000) and very good selectivity compared with CYPs 2C9, 2C19 and 3A4 (5904, 892 and 4072 respectively) (Table 3).

**Table 3:** CYP IC_50_ (μM) profile of compound 10c

CYP isoform	IC_50_ (μM)	Selectivity CYP19A1
1A2	>25	30 120
2C9	4.90 ± 0.94	5904
2C19	0.74 ± 0.16	892
2D6	>25	30 120
3A4	3.38 ± 0.51	4072
19A1	0.00083	—

### Computational studies

**Fig. 5:**
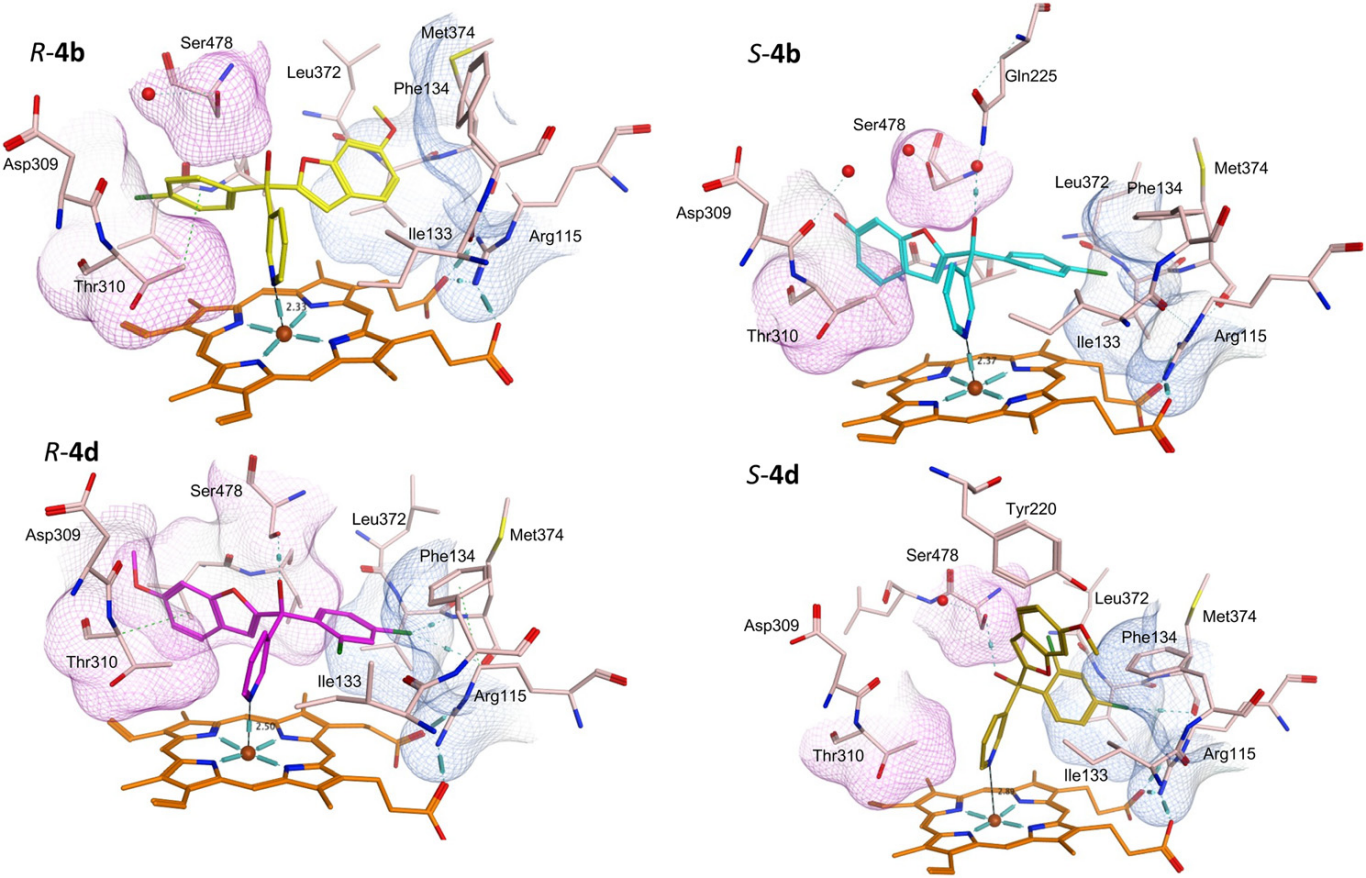
Positioning of the enantiomers of exemplar methoxy derivatives 4b and 4d in the haem active site of CYP19A1. Pocket lined by Arg115, Ile133, Phe134 and Met374 in blue, pocket lined by Asp309, Thr310 and Ser478 in pink.

Protein-ligand complexes of both *R*- and *S*-enantiomers of compounds 4, 10 and 11 were prepared by docking the compounds with the X-ray crystal structure of CYP19A1 (PDB 3S79)^14^ using molecular operating environment (MOE) software.^15^ The prepared protein–ligand complexes were then subject to 200 ns molecular dynamics simulations using the Desmond programme of Schrödinger Maestro software.^16,17^ The methoxy derivatives 4a, 4c and 4d were consistent in the positioning of the *R*- and *S*-enantiomers in the haem active site, with the *R*-enantiomers positioned with the benzofuran moiety in the pocket lined by Arg115, Ile133, Phe134 and Met374 and the phenyl halide moiety in the pocket lined by Asp309, Thr310 and Ser478, while this positioning was flipped in the *S*-enantiomers (*e.g.*4bFig. 5 and S1†). For the dichloro derivative (4d), the *R*-enantiomer was orientated similarly to the *S*-enantiomers of 4a-c, however for *S*-4d the benzofuran is not positioned in the expected Asp309, Thr310 and Ser478 pocket (Fig. 5) and the pyridine N is further away from the haem (2.89 Å) compared with the monosubstituted phenyl halide derivatives (4a–c) (2.33–2.57 Å) (Table 4), which may account for the reduced CYP19A1 inhibition observed for 4d (Table 2).

**Table 4:** Pyridine N–Fe^3+^ haem distance and main binding interactions for methoxy derivatives (4)

Cmpd	N–Fe^3+^ (Å)	Binding interactions	Compd	N–Fe^3+^ (Å)	Binding interactions
*R*-4a	2.46	O*H*–Ser478 (HB), *O*H–Asp309 (*via* H_2_O)	*S*-4a	2.65	*O*CH_3_–Ser478 (*via* H_2_O)
		*O*CH_3_–Met374 (HB)			Pyridine–Ile333 (VdW)
		BF *benzene*–Val373 (VdW)			
					F-*phenyl*–Phe134 (π–π)
*R*-4b	2.33	Cl-*phenyl*–Phe310 (VdW)	*S*-4b	2.37	O*H*–Gln225 (*via* H_2_O)
		*O*CH_3_–Met374 (HB)			
					*Cl*–Met374 (H-Hal)
*R*-4c	2.50	O*H*–Ser478 (HB)	*S*-4c	2.57	*Br*–Met374 (H-Hal)
		Br-*phenyl*–Ser478 (VdW)			O*H*–Ser478 (*via* H_2_O)
*R*-4d	2.50	O*H*–Ser478 (HB)	*S*-4d	2.89	O*H*–Ser478 (HB)
		BF *benzene*–Thr310 (VdW)			BF *benzene*–Trp224 (VdW)
		*Cl*–Met374 (H-Hal)			
					*O*CH_3_–Tyr220 (HB)
					*Cl*–Met374 (H-Hal)

**Fig. 6:**
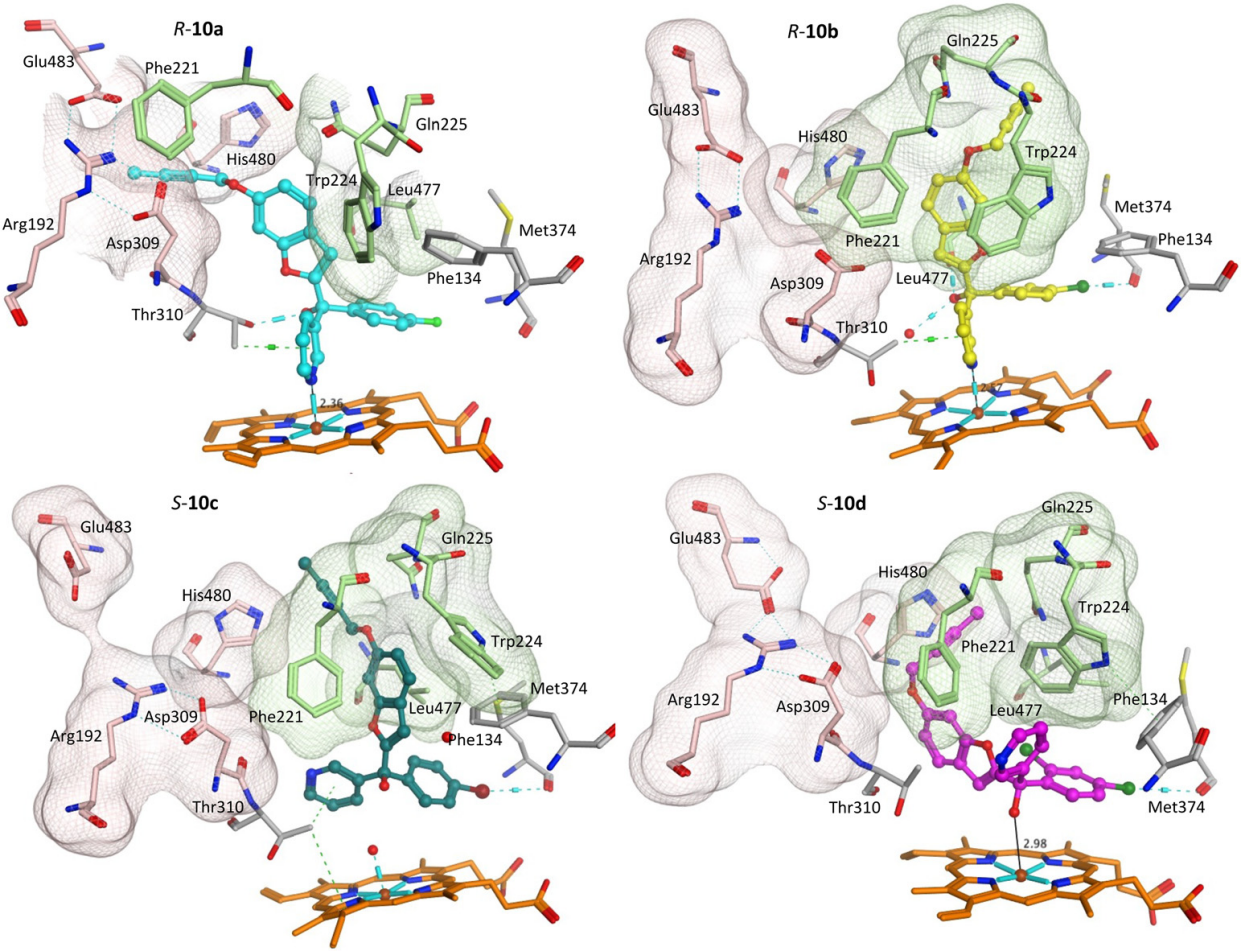
Positioning of the enantiomers of exemplar but-2-ynyloxy derivatives (10) in the haem active site of CYP19A1 and access channels. Front door access channel lined by Arg192, Asp309, His480 and Glu483 (light pink) while an alternative access channel is lined by Phe221, Trp224, Gln225 and Leu477 (green).

Addition of the but-2-ynyloxy group resulted in compounds (10) that were more sterically restrained within the CYP19A1 active site, with the *S*-enantiomers unfavourable compared with the *R*-enantiomers for all substitutions (10a-d) (Fig. S2† and exemplars Fig. 6).

Both enantiomers of the fluoro derivative (10a) and the *R*-enantiomer of the dichloro derivative (*R*-10d) were positioned in the haem active site and the front door access channel (*e.g. R*-10a, Fig. 6) as previously observed for the triazole derivatives (Fig. 1), however with limited binding interactions observed (Table 5). Although both enantiomers of the bromo derivative (10c) and the *R*-enantiomer of the chloro derivative (*R*-10b) were positioned in the haem active site, the benzofuran but-2-ynyloxy portion was positioned in a different site (*e.g. R*-10b and *S*-10c, Fig. 6) involving Phe221, Trp224, Gln225 and Leu477. The *S*-enantiomers of the bromo (10c) and dichloro derivative (10d) did not bind to the haem *via* the pyridine N, however they did bind with the haem iron through the hydroxy group either directly (*S*-10d, Fig. 6) or water mediated (*S*-10c) (Table 5).

**Table 5:** Pyridine N–Fe^3+^ haem distance and main binding interactions of compounds 10 and 11

Compd	N–Fe^3+^ (Å)	Binding interactions	Compd	N–Fe^3+^ (Å)	Binding interactions
*R*-10a	2.36	*O*H–Thr310 (HB)	*R*-11a	2.61	O*H*–Ser478 (HB)
		Pyridine–Thr310 (VdW)			
*S*-10a	2.52	BF *benzene*–Thr310 (VdW)	*S*-11a	2.97	*O*H–Gln225 (HB), *O*H–H_2_O (HB)
*R*-10b	2.57	*Cl*–Met374 (H-Hal)	*R*-11b	2.43	*Cl*–Met374 (H-Hal)
		Pyridine–Thr310 (VdW)			
		O*H*–Leu477 (HB), *O*H–H_2_O (HB)			
*S*-10b	—	*Cl*–Met374 (H-Hal)	*S*-11b	2.43	*Cl*–Met374 (H-Hal)
		Pyridine–Trp224 (VdW)			
		O*H*–Leu477 (HB), *O*H–H_2_O (HB)			
*R*-10c	2.37	O*H*–Ser478 (HB), *O*H–H_2_O (HB)	*R*-11c	2.75	*Br*–Met374 (H-Hal)
		*O*-Butyne–Gln225 (*via* H_2_O)			
		*Br*–Met374 (H-Hal)			
		Br-*phenyl*–Leu477 (VdW)			
*S*-10c	—	*Br*–Met374 (H-Hal)	*S*-11c	2.55	*Br*–Met374 (H-Hal)
					BF *benzene*–Thr310 (VdW)
		*O*H – Fe haem (*via* H_2_O 3.27 Å)			
					O*H*–H_2_O
		Pyridine–Thr310 (VdW)			
		*O*-Butyne–Gln225 (HB)			
*R*-10d	2.60	*Cl*–Ile133 (*via* H_2_O)	*R*-11d	2.40	Furan–haem (VdW)
		Pyridine–Thr310 (VdW)			*OH*–H_2_O
		Furan–haem (VdW)			
*S*-10d	—	*O*H–Fe haem (2.98 Å)	*S*-11d	—	*Cl*–Met374 (H-Hal)
		*Cl*–Met374 (H-Hal)			BF *benzene*–Thr310 (VdW)
					Furan–Thr310 (VdW)
					*O*H–Fe haem (3.30 Å)

**Fig. 7:**
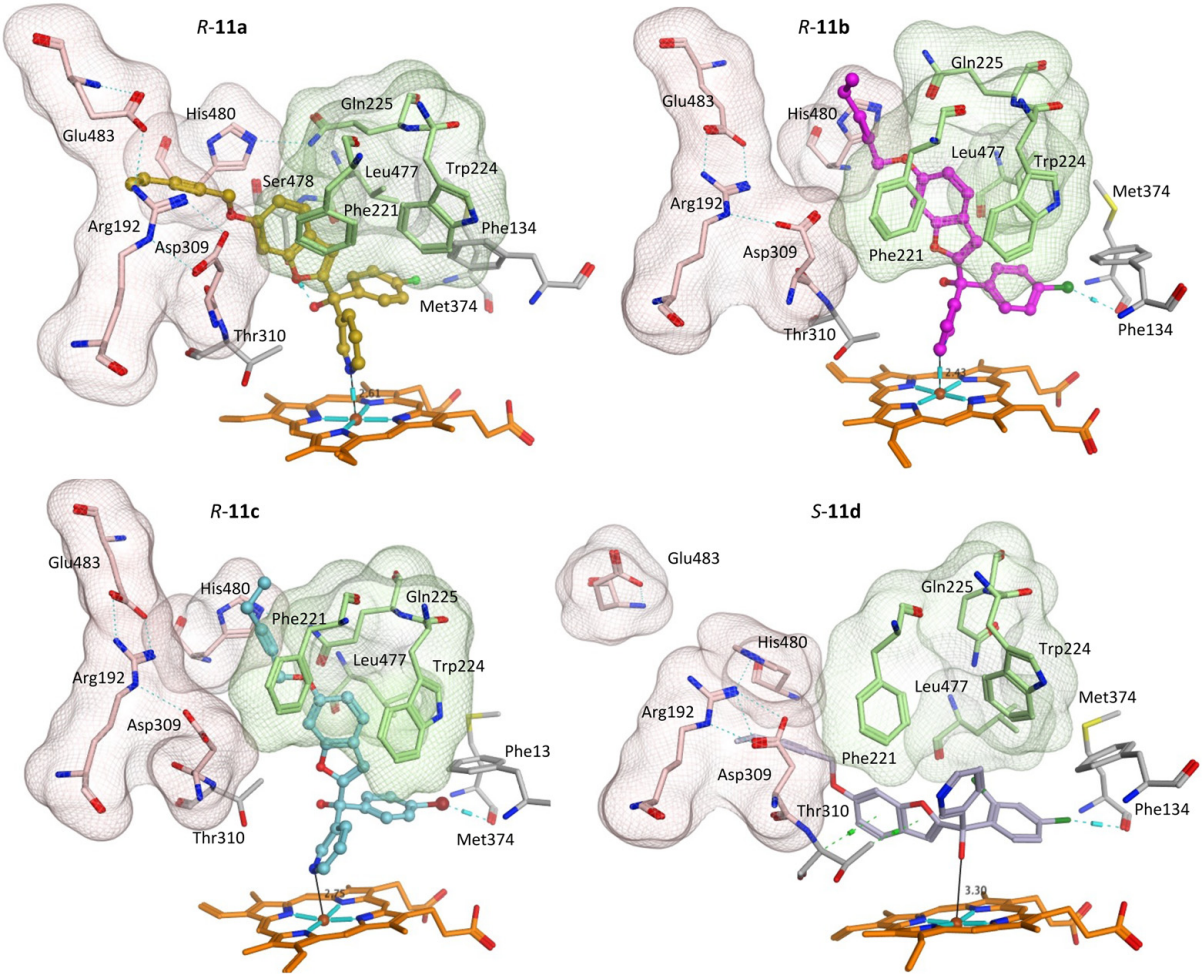
Positioning of the enantiomers of exemplar pent-2-ynyloxy derivatives (11) in the haem active site of CYP19A1 and access channels. Front door access channel lined by Arg192, Asp309, Pro481, His480 and Glu483 (light pink) while an alternative access channel is lined by Phe221, Trp224, Gln225 and Leu477 (green).

The greater flexibility of pent-2-ynyloxy group, with the additional sp^3^ CH_2_, allowed better fit within the binding sites. With the exception of the *S*-enantiomer of 11d, all the pent-2-ynyloxy derivatives (11) bind with the haem through the N of pyridine (Fig. S3†), although *S*-11d does bind with the Fe of the haem through the hydroxy group (Fig. 7) but does not extend into the access channel.

With the exception of *R*-11b and *R*-11c, the pent-2-ynyloxy group is positioned in the front door access channel lined by Arg192, Asp309, His480 and Glu483 (*e.g. R*-11a, Fig. 7), while for *R*-11b the pent-2-ynyloxy group is positioned in the alternative access channel lined by Phe221, Trp224, Gln225 and Leu477 and for *R*-11c the pent-2-ynyloxy group sits between the two access channels (Fig. 7). The chloro derivative (11b) is most closely positioned to the haem (N–Fe^3+^ distance 2.43 Å, Table 5) and full extension along the access channels, which may account for the optimal CYP19A1 inhibition observed for this alkynyloxy group. The *S*-enantiomer of the fluoro derivative (*S*-11a) does not extend fully into the access channel and has the largest N–Fe^3+^ binding (2.97 Å) (Table 5).

## Conclusions

**Fig. 8:**
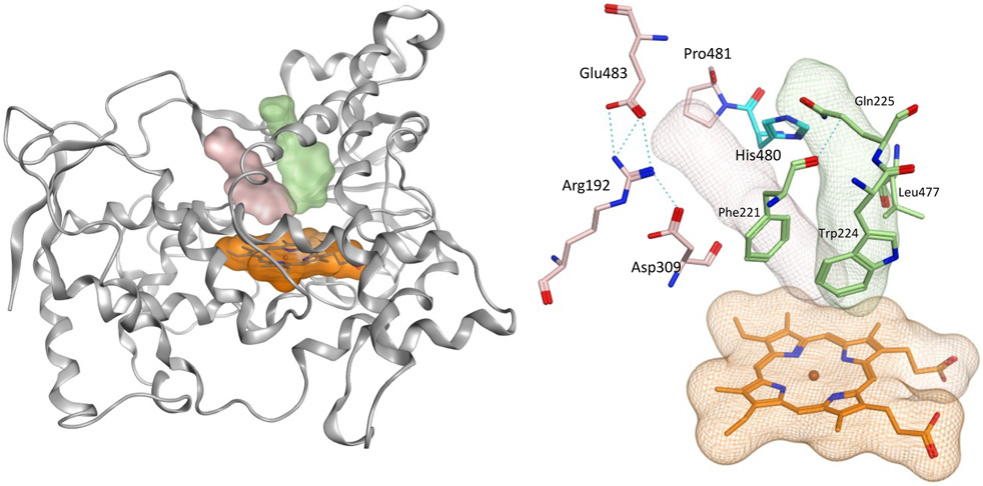
Positioning of the haem active site (orange), front door access channel (light pink) lined by Arg192, Asp309, Pro481 and Glu483 and an alternative access channel (green) lined by Phe221, Trp224, Gln225 and Leu477 within the CYP19A1 protein (grey ribbon). His480 (cyan) is on the border of both access channels.

The methoxy derivatives (4) were all positioned within the haem binding site with the dichloro derivative (4d) least optimal with respect to binding and CYP19A1 inhibitory activity and this trend was also observed for the but-2-ynyloxy (10d, IC_50_ > 10 nM) and pent-2-ynyloxy (11d, IC_50_ > 10 nM) derivatives. The but-2-ynyloxy derivatives (10) were the most sterically restricted, in particular the *S*-enantiomers (Table 5, Fig. 6 and S2†). The but-2-ynyloxy group was found to occupy either the front door access channel (Fig. 8, pink) or an alternative access channel (Fig. 8, green), with both enantiomers of the most active bromo derivative (10c, IC_50_ 0.83 nM) found to occupy the alternative access channel lined by Phe221, Trp224, Gln225 and Leu477 (Fig. 6) with multiple binding interactions observed (Table 5).

In contrast the pent-2-ynyloxy derivatives (11), with the addition of a CH_2_ group in the alkynyloxy chain had more flexibility compared with the but-2-ynyloxy derivatives, with more of the derivatives binding in the front door access channel (Fig. 5 and S3†) as previously observed for the triazole derivatives (Fig. 1).^10^ The most active chloro derivative (11b, IC_50_ 0.92 nM) was found to bind in both access channels (*R*-11b, alternative access channel; *S*-11b, front door access channel) and had the optimal N–Fe^3+^ binding distance (*R*/*S*-11b, 2.43 Å, Table 5). The computational identification of an alternative access channel provides further insight into the potential binding mode and interactions of the non-steroidal AIs.

The pyridine AIs with IC_50_ < 10 nM were found to be non-toxic against MDA-MB-231, MCF-7 and MCF-10A cells (Fig. 4). The current 3rd generation AIs have excellent CYP19A1 selectivity but letrozole shows significant competitive inhibition of CYP2A6 and modest inhibition of CYP2C19,^18,19^ while anastrozole was reported with some inhibition of CYP1A2, CYP2C9 and CYP3A4.^19,20^ Selectivity is a very important criterion for AIs and, although the final lead compound (10c) displayed excellent selectivity against a human CYP panel (Table 3), improvement in selectivity against 2C9, 2C19 and 3A4 is desirable. The extended derivatives 10c (IC_50_ 0.83 nM) and 11b (IC_50_ 0.92 nM) were also found to have a profile similar to the standard AI letrozole, potently inhibiting CYP19A1 (IC_50_ 0.70 nM). Further research, including resolution of enantiomers to investigate individual *R*- and *S*-enantiomers with respect to inhibitory activity, cytotoxicity and selectivity, as well as establishing whether these extended pyridine dual binding site AIs are effective against AI resistant breast cancer cell lines are therefore warranted.

## Experimental

### General

All chemicals, reagents and solvents were purchased from Sigma-Aldrich, Alfa Aesar, VWR, Acros and Fluka. Solvents were dried prior to use over molecular sieves (4 Å). For column chromatography, a glass column was slurry packed in the appropriate eluent with silica gel (Fluka Kieselgel 60). TLC was performed on pre-coated silica plates (dimension 20 × 20 cm) (ALUGRAM® SIL G/UV_254_) with visualisation *via* UV light (254 nm). Melting points were determined on an electrothermal instrument (Gallenkamp) and were uncorrected. ^1^H, ^13^C, and ^19^F NMR spectra were recorded on a Bruker Advance DP500 spectrometer operating at 500, 125 and 470 MHz, respectively. Chemical shifts are given in parts per million (ppm) relative to the internal standard tetramethylsilane (Me_4_Si). Elemental analysis was performed by MEDAC Ltd (Chobham, Surrey, UK) and HPLC-HRMS was performed at the Department of Pharmacy & Pharmacology, University of Bath, Bath, UK. on a Zorbax Eclipse Plus C18 Rapid Resolution 2.1 × 50 mm, 1.8 μm particle size using a 7.5 minute gradient method 5 : 95 v/v water : methanol with 0.1% formic acid as additive. Experimental details for intermediates 3 and 6–9 are provided in the ESI.†

### Chemistry

#### General method for the synthesis of (6-methoxybenzofuran-2-yl)(phenyl)(pyridin-3-yl)methanol derivatives (4, 10 and 11)

To a solution of (6-methoxybenzofuran-2-yl)(phenyl)methanone derivatives (3, 8 or 9) (1 m.eq.) in THF (10 mL mmol^−1^) was added pyridin-3-yl magnesium bromide (7 m.eq.) and the reaction mixture was heated at 70 °C overnight. The reaction mixture was concentrated under reduced pressure and the resulting residue extracted between H_2_O (50 mL) and EtOAc (2 × 100 mL). The combined organic layers were combined, dried (MgSO_4_) and concentrated under vacuum. The product was purified by gradient column chromatography to give (6-*O*-alkyl/alkyne-benzofuran-2-yl)(phenyl)(pyridin-3-yl)methanol derivatives (4, 10 or 11) at 60% EtOAc in petroleum ether (v/v) as a colourless oil.

#### (4-Fluorophenyl)(6-methoxybenzofuran-2-yl)(pyridin-3-yl)methanol (4a: R = 4-F)^11^

Prepared from (4-fluorophenyl)(6-methoxybenzofuran-2-yl)methanone (3a) (0.19 g, 0.70 mmol).Yield: 0.2 g (83%); *R*_f_ = 0.42 (petroleum ether–EtOAc 1 : 1 v/v). ^1^H NMR (DMSO-d_6_): *δ* 8.52 (m, 2H, Ar), 7.71 (m, 1H, Ar), 7.49 (d, *J* = 8.6 Hz, 1H, Ar), 7.41 (m, 3H, Ar), 7.21 (m, 3H, Ar), 7.16 (bs, 1H, OH), 6.88 (dd, *J* = 2.3, 8.6 Hz, 1H, Ar), 6.47 (d, *J* = 0.9 Hz, 1H, Ar), 3.77 (s, 3H, OCH_3_). ^13^C NMR (DMSO-d_6_): *δ* 162.86 (d, ^1^*J*_C,F_ = 243.75 Hz, C), 156.63 (C), 158.23 (C), 156.17 (C), 148.93 (CH), 148.61 (CH), 141.22 (d, ^4^*J*_C,F_ = 2.5 Hz, C), 141.00 (C), 135.00 (CH), 129.52 (d, ^3^*J*_C,F_ = 8.75 Hz, 2× CH), 123.56 (CH), 122.05 (CH), 120.96 (C), 115.36 (d,^2^*J*_C,F_ = 21.25 Hz, 2× CH), 112.50 (CH), 106.10 (CH), 96.44 (CH), 75.86 (C), 56.02 (CH_3_). HPLC: 100% at R.T. = 4.61 min. HRMS (ESI) calculated 350.1187 [M + H]^+^, found 350.1189 [M + H]^+^.

#### (4-Chlorophenyl)(6-methoxybenzofuran-2-yl)(pyridin-3-yl)methanol (4b: R = 4-Cl)^11^

Prepared from (4-chlorophenyl)(6-methoxybenzofuran-2-yl)methanone (3b) (0.45 g, 1.5 mmol).Yield: 0.5 g (92%); *R*_f_ = 0.4 (petroleum ether–EtOAc 1 : 1 v/v). ^1^H NMR (CDCl_3_): *δ* 8.57 (d, *J* = 1.7 Hz, 1H, Ar), 8.52 (d, *J* = 4.7 Hz, 1H, Ar), 7.78 (dt, *J* = 2.15, 8.1 Hz, 1H, Ar), 7.38 (d, *J* = 8.6 Hz, 1H, Ar), 7.34 (m, 5H, Ar), 6.96 (d, *J* = 2.0 Hz, 1H, Ar), 6.90 (dd, *J* = 2.3, 8.6 Hz, 1H, Ar), 6.27 (d, *J* = 0.8 Hz, 1H, Ar), 3.84 (s, 3H, OCH_3_), 2.37 (bs, 1H, OH). ^13^C NMR (CDCl_3_): *δ* 158.42 (C), 157.61 (C), 156.28 (C), 148.32 (CH), 148.11 (CH), 141.89 (C), 139.97 (C), 135.55 (CH), 134.22 (C), 128.67 (2× CH), 128.51 (2× CH), 123.21 (CH), 121.58 (CH), 120.69 (C), 112.37 (CH), 106.82 (CH), 96.04 (CH), 76.51 (C), 55.72 (CH_3_). HPLC: 100% at R.T. = 4.73 min. HRMS (ESI) calculated (^35^Cl) 366.0896 [M + H]^+^, found 366.0891 [M + H]^+^; calculated (^37^Cl) 368.0868 [M + H]^+^, found 368.0872 [M + H]^+^.

#### (4-Bromophenyl)(6-methoxybenzofuran-2-yl)(pyridin-3-yl)methanol (4c: R = 4-Br)

Prepared from (4-bromophenyl)(6-methoxybenzofuran-2-yl)methanone (3c) (0.43 g, 1.29 mmol).Yield: 0.13 g (25%); *R*_f_ = 0.47 (petroleum ether–EtOAc 1 : 1 v/v). ^1^H NMR (DMSO-d_6_): *δ* 8.52 (m, 2H, Ar), 7.71 (m, 1H, Ar), 7.58 (d, *J* = 8.7 Hz, 2H, Ar), 7.49 (d, *J* = 8.5 Hz, 1H, Ar), 7.41 (m, 1H, Ar), 7.30 (d, *J* = 8.7 Hz, 2H, Ar), 7.16 (m, 2H, Ar + OH), 6.88 (dd, *J* = 2.3, 8.5 Hz, 1H, Ar), 6.48 (d, *J* = 0.9 Hz, 1H, Ar), 3.77 (s, 3H, OCH_3_). ^13^C NMR (DMSO-d_6_): *δ* 159.26 (C), 158.26 (C), 156.18 (C), 149.01 (CH), 148.58 (CH), 144.38 (C), 140.71 (C), 135.01 (CH), 131.46 (2× CH), 129.63 (2× CH), 123.60 (CH), 122.08 (CH), 121.36 (C), 120.94 (C), 112.53 (CH), 106.25 (CH), 96.44 (CH), 75.92 (C), 56.03 (CH_3_). HPLC: 98.9% at R.T. = 4.76 min. HRMS (ESI) calculated (^79^Br) 410.0391 [M + H]^+^, found 410.0387 [M + H]^+^; calculated (^81^Br) 412.0372 [M + H]^+^, found 412.0369 [M + H]^+^.

#### (2′,4′-Dichlorophenyl)(6-methoxybenzofuran-2-yl)(pyridin-3-yl)methanol (4d: R = 2,4-diCl)

Prepared from (2′,4′-dichlorophenyl)(6-methoxybenzofuran-2-yl)methanone (3d) (0.45 g, 1.4 mmol). Yield: 0.18 g (32%); *R*_f_ = 0.5 (petroleum ether–EtOAc 1 : 1 v/v). ^1^H NMR (CDCl_3_): *δ* 8.49 (m, 2H, Ar), 7.69 (m, 1H, Ar), 7.34 (d, *J* = 2.1 Hz, 1H, Ar), 7.30 (d, *J* = 8.6 Hz, 1H, Ar), 7.23 (m, 1H, Ar), 7.14 (dd, *J* = 2.1, 8.5 Hz, 1H, Ar), 7.01 (d, *J* = 8.5 Hz, 1H, Ar), 6.91 (d, *J* = 2.1 Hz, 1H, Ar), 6.81 (dd, *J* = 2.3, 8.6 Hz, 1H, Ar), 6.22 (d, *J* = 0.9 Hz, 1H, Ar), 4.39 (bs, 1H, OH), 3.75 (s, 3H, OCH_3_). ^13^C NMR (CDCl_3_): *δ* 158.42 (C), 156.35 (C), 156.19 (C), 148.99 (CH), 148.56 (CH), 139.06 (C), 138.38 (C), 135.21 (C), 134.82 (CH), 133.89 (C), 131.43 (CH), 131.09 (CH), 127.02 (CH), 123.02 (CH), 121.63 (CH), 120.76 (C), 112.48 (CH), 106.99 (CH), 96.09 (CH), 77.18 (C), 55.72 (CH_3_). HPLC: 100% at R.T. = 4.78 min. HRMS (ESI) calculated (^35^Cl) 400.0508 [M + H]^+^, found 400.0532 [M + H]^+^; calculated (^37^Cl) 402.0478 [M + H]^+^, found 402.0494 [M + H]^+^.

#### (6-(But-2-yn-1-yloxy)benzofuran-2-yl)(4-fluorophenyl)(pyridin-3-yl)methanol (10a: R = 4-F)

Prepared from (6-(but-2-yn-1-yloxy)benzofuran-2-yl)(4-fluorophenyl)methanone (8a) (0.1 g, 0.32 mmol). Yield: 0.1 g (80%); *R*_f_ = 0.37 (petroleum ether–EtOAc 1 : 1 v/v). ^1^H NMR (CDCl_3_): *δ* 8.48 (m, 2H, Ar), 7.65 (m, 1H, Ar), 7.30 (d, *J* = 8.6 Hz, 1H, Ar), 7.26 (dd, *J* = 5.3, 8.9 Hz, 2H, Ar), 7.20 (m, 1H, Ar), 6.98 (m, 3H, Ar), 6.85 (dd, *J* = 2.3, 8.6 Hz, 1H, Ar), 6.15 (d, *J* = 0.8 Hz, 1H, Ar), 4.59 (q, *J* = 2.3 Hz, 2H, CH_2_), 3.90 (bs, 1H, OH), 1.78 (t, *J* = 2.3 Hz, 3H, CH_3_). ^13^C NMR (CDCl_3_): *δ* 163.46 (d, ^1^*J*_C,F_ = 246.25 Hz, C), 158.23 (C), 156.52 (C), 156.03 (C), 148.92 (CH), 148.73 (CH), 139.68 (C), 139.24 (d, ^4^*J*_C,F_ = 2.5 Hz, C), 134.98 (CH), 129.16 (d, ^3^*J*_C,F_ = 8.75 Hz, 2× CH), 123.02 (CH), 121.55 (CH), 121.29 (C), 115.29 (d, ^2^*J*_C,F_ = 21.25 Hz, 2× CH), 112.96 (CH), 106.72 (CH), 97.41 (CH), 84.06 (C), 76.60 (C), 73.83 (C), 57.02 (CH_2_), 3.74 (CH_3_). HPLC: 100% at R.T. = 4.70 min. HRMS (ESI) calculated 388.1348 [M + H]^+^, found 388.1351 [M + H]^+^.

#### (6-(But-2-yn-1-yloxy)benzofuran-2-yl)(4-chlorophenyl)(pyridin-3-yl)methanol (10b: R = 4-Cl)

Prepared from (6-(but-2-yn-1-yloxy)benzofuran-2-yl)(4-chlorophenyl)methanone (8b) (0.14 g, 0.42 mmol). Yield: 0.16 g (94%); *R*_f_ = 0.4 (petroleum ether–EtOAc 1 : 1 v/v). ^1^H NMR (CDCl_3_): *δ* 8.60 (m, 2H, Ar), 7.74 (dt, *J* = 1.6, 8.0 Hz, 1H, Ar), 7.40 (d, *J* = 8.6 Hz, 1H, Ar), 7.36 (m, 5H, Ar), 7.08 (s, 1H, Ar), 6.95 (dd, *J* = 2.2, 8.6 Hz, 1H, Ar), 6.26 (s, 1H, Ar), 4.69 (q, *J* = 2.2 Hz, 2H, CH_2_), 3.74 (bs, 1H, OH), 1.88 (t, *J* = 2.2 Hz, 3H, CH_3_). ^13^C NMR (CDCl_3_): *δ* 157.84 (C), 156.55 (C), 156.05 (C), 149.12 (CH), 148.76 (CH), 141.81 (C), 139.40 (C), 134.92 (CH), 134.27 (C), 128.68 (2× CH), 128.52 (2× CH), 123.05 (CH), 121.59 (CH), 121.24 (C), 113.04 (CH), 106.89 (CH), 97.40 (CH), 84.08 (C), 76.65 (C), 73.80 (C), 57.03 (CH_2_), 3.74 (CH_3_). HPLC: 100% at R.T. = 4.80 min. HRMS (ESI) calculated (^35^Cl) 404.1009 [M + H]^+^, found 404.1053 [M + H]^+^; calculated (^37^Cl) 406.0979 [M + H]^+^, found 406.1030 [M + H]^+^.

#### (4-Bromophenyl)(6-(but-2-yn-1-yloxy)benzofuran-2-yl)(pyridin-3-yl)methanol (10c: R = 4-Br)

Prepared from (6-(but-2-yn-1-yloxy)benzofuran-2-yl)(4-bromophenyl)methanone (8c) (0.09 g, 0.24 mmol). Yield: 0.07 g (70%); *R*_f_ = 0.42 (petroleum ether–EtOAc 1 : 1 v/v). ^1^H NMR (CDCl_3_): *δ* 8.47 (m, 2H, Ar), 7.64 (dt, *J* = 2.1, 8.1 Hz, 1H, Ar), 7.40 (d, *J* = 8.7 Hz, 2H, Ar), 7.30 (d, *J* = 8.6 Hz, 1H, Ar), 7.20 (m, 3H, Ar), 6.97 (d, *J* = 2.1 Hz, 1H, Ar), 6.85 (dd, *J* = 2.3, 8.6 Hz, 1H, Ar), 6.16 (d, *J* = 0.9 Hz, 1H, Ar),), 4.59 (q, *J* = 2.3 Hz, 2H, CH_2_), 3.97 (bs, 1H, OH), 1.78 (t, *J* = 2.3 Hz, 3H, CH_3_). ^13^C NMR (CDCl_3_): *δ* 157.87 (C), 156.56 (C), 156.04 (C), 148.98 (CH), 148.66 (CH), 142.43 (C), 139.36 (C), 134.98 (CH), 131.46 (2× CH), 129.00 (2× CH), 123.05 (CH), 122.44 (C), 121.58 (CH), 121.24 (C), 113.01 (CH), 106.85 (CH), 97.39 (CH), 84.07 (C), 76.64 (C), 73.82 (C), 57.02 (CH_2_), 3.75 (CH_3_). HPLC: 100% at R.T. = 4.83 min. HRMS (ESI) calculated (^79^Br) 448.0504 [M + H]^+^, found 448.0543 [M + H]^+^; calculated (^81^Br) 450.0483 [M + H]^+^, found 450.0527 [M + H]^+^.

#### (6-(But-2-yn-1-yloxy)benzofuran-2-yl)(2,4-dichlorophenyl)(pyridin-3-yl)methanol (10d: R = 2,4-diCl)

Prepared from (6-(but-2-yn-1-yloxy)benzofuran-2-yl)(2,4-dichlorophenyl) methanone (8d) (0.19 g, 0.52 mmol). Yield: 0.08 g (34%); *R*_f_ = 0.4 (petroleum ether–EtOAc 1 : 1 v/v). ^1^H NMR (CDCl_3_): *δ* 8.52 (m, 2H, Ar), 7.69 (dt, *J* = 1.8, 8.2 Hz, 1H, Ar), 7.36 (d, *J* = 2.2 Hz, 1H, Ar), 7.32 (d, *J* = 8.6 Hz, 1H, Ar), 7.25 (m, 1H, Ar), 7.15 (dd, *J* = 2.2, 8.6 Hz, 1H, Ar), 7.03 (d, *J* = 2.1 Hz, 1H, Ar), 7.00 (d, *J* = 8.5 Hz, 1H, Ar), 6.87 (dd, *J* = 2.3, 8.6 Hz, 1H, Ar), 6.23 (d, *J* = 0.9 Hz, 1H, Ar), 4.61 (q, *J* = 2.3 Hz, 2H, CH_2_), 4.06 (bs, 1H, OH), 1.80 (t, *J* = 2.3 Hz, 3H, CH_3_). ^13^C NMR (CDCl_3_): *δ* 156.60 (C), 156.47 (C), 155.97 (C), 149.16 (CH), 148.63 (CH), 138.92 (C), 138.24 (C), 135.29 (C), 134.78 (CH), 133.84 (C), 131.45 (CH), 131.13 (CH), 127.07 (CH), 123.06 (CH), 121.65 (CH), 121.28 (C), 113.13 (CH), 107.09 (CH), 97.47 (CH), 84.09 (C), 76.77 (C, obscured by CDCl3), 73.80 (C), 57.04 (CH_2_), 3.75 (CH_3_). HPLC: 100% at R.T. = 4.84 min. HRMS (ESI) calculated (^35^Cl) 438.0619 [M + H]^+^, found 438.0659 [M + H]^+^; calculated (^37^Cl) 440.0590 [M + H]^+^, found 440.06354 [M + H]^+^.

#### (4-Fluorophenyl)(6-(pent-2-yn-1-yloxy)benzofuran-2-yl)(pyridin-3-yl)methanol (11a: R = 4-F)

Prepared from (4-fluorophenyl)(6-(pent-2-yn-1-yloxy)benzofuran-2-yl)methanone (9a) (0.17 g, 0.527 mmol). Yield: 0.04 g (20%); *R*_f_ = 0.45 (petroleum ether–EtOAc 1 : 1 v/v). ^1^H NMR (CDCl_3_): *δ* 8.53 (m, 2H, Ar), 7.66 (d, *J* = 7.9 Hz, 1H, Ar), 7.31 (d, *J* = 8.6 Hz, 1H, Ar), 7.28 (dd, *J* = 5.3, 8.9 Hz, 2H, Ar), 7.19 (obscured by CDCl3, 1H, Ar), 7.00 (d, *J* = 2.1 Hz, 1H, Ar), 6.99 (t, *J* = 8.7 Hz, 2H, Ar), 6.86 (dd, *J* = 2.3, 8.6 Hz, 1H, Ar), 6.16 (d, *J =* 0.8 Hz, 1H, Ar), 4.62 (t, *J* = 2.1 Hz, 2H, CH_2_), 3.32 (bs, 1H, OH), 2.19 (qt, *J* = 2.1, 7.5 Hz, 2H, CH_2_), 1.08 (t, *J* = 7.5 Hz, 3H, CH_3_). ^13^C NMR (CDCl_3_): *δ* 176.59 (C), 163.49 (d, ^1^*J*_C,F_ = 247.5 Hz, C), 158.11 (C), 156.58 (C), 156.00 (C), 139.15 (d, ^4^*J*_C,F_ = 3.75 Hz, C), 134.83 (CH), 129.17 (d, ^3^*J*_C,F_ = 8.75 Hz, 2× CH), 121.54 (CH), 121.26 (C), 115.33 (d, ^2^*J*_C,F_ = 22.5 Hz, 2× CH), 113.05 (CH), 106.83 (CH), 97.48 (CH), 89.83 (C), 76.76 (C, obscured by CDCl_3_), 73.94 (C), 57.13 (CH_2_), 13.58 (CH_3_), 12.51 (CH_2_). *three CH pyridine peaks too small to be detected. HPLC: 100% at R.T. = 4.78 min. HRMS (ESI) calculated 402.1505 [M + H]^+^, found 402.1501 [M + H]^+^.

#### (4-Chlorophenyl)(6-(pent-2-yn-1-yloxy)benzofuran-2-yl)(pyridin-3-yl)methanol (11b: R = 4-Cl)

Prepared from (4-chlorophenyl)(6-(pent-2-yn-1-yloxy)benzofuran-2-yl)methanone (9b) (0.16 g, 0.47 mmol). Yield: 0.18 g (91%); *R*_f_ = 0.32 (petroleum ether–EtOAc 1 : 1 v/v). ^1^H NMR (CDCl_3_): *δ* 8.49 (m, 2H, Ar), 7.64 (m, 1H, Ar), 7.30 (d, *J* = 8.6 Hz, 1H, Ar), 7.26 (m, 5H, Ar), 6.98 (d, *J* = 2.0 Hz, 1H, Ar), 6.86 (dd, *J* = 2.3, 8.6 Hz, 1H, Ar), 6.16 (d, *J* = 0.9 Hz, 1H, Ar), 4.61 (t, *J* = 2.1 Hz, 2H, CH2), 3.79 (bs, 1H, OH), 2.18 (qt, *J* = 2.1, 7.5 Hz, 2H, CH_2_), 1.07 (t, *J* = 7.5 Hz, 3H, CH_3_). ^13^C NMR (CDCl_3_): *δ* 157.90 (C), 156.61 (C), 156.04 (C), 149.02 (CH), 148.70 (CH), 141.87 (C), 139.46 (C), 134.96 (CH), 134.23 (C), 128.68 (2× CH), 128.51 (2× CH), 123.05 (CH), 121.55 (CH), 121.24 (C), 113.05 (CH), 106.86 (CH), 97.48 (CH), 89.87 (C), 76.61 (C), 73.97 (C), 57.13 (CH_2_), 13.58 (CH_3_), 12.51 (CH_2_). HPLC: 100% at R.T. = 4.87 min. HRMS (ESI) calculated (^35^Cl) 418.1165 [M + H]^+^, found 418.1204 [M + H]^+^; calculated (^37^Cl) 420.1136 [M + H]^+^, found 420.1187 [M + H]^+^.

#### (4-Bromophenyl)(6-(pent-2-yn-1-yloxy)benzofuran-2-yl)(pyridin-3-yl)methanol (11c: R = 4-Br)

Prepared from (4-bromophenyl)(6-(pent-2-yn-1-yloxy)benzofuran-2-yl)methanone (9c) (0.09 g, 0.23 mmol). Yield: 0.05 g (50%); *R*_f_ = 0.50 (petroleum ether–EtOAc 1 : 1 v/v). ^1^H NMR (CDCl_3_): *δ* 8.48 (m, 2H, Ar), 7.64 (d, *J* = 8.0 Hz, 1H, Ar), 7.41 (d, *J* = 8.6 Hz, 2H, Ar), 7.30 (d, *J* = 8.6 Hz, 1H, Ar), 7.20 (m, 3H, Ar), 6.98 (d, *J* = 2.1 Hz, 1H, Ar), 6.86 (dd, *J* = 2.3, 8.6 Hz, 1H, Ar), 6.16 (d, *J* = 0.9 Hz, 1H, Ar), 4.61 (t, *J* = 2.1 Hz, 2H, CH_2_), 3.68 (bs, 1H, OH), 2.19 (qt, *J* = 2.1, 7.5 Hz, 2H, CH_2_), 1.07 (t, *J* = 7.5 Hz, 3H, CH_3_). ^13^C NMR (CDCl_3_): *δ* 157.79 (C), 156.62 (C), 156.04 (C), 148.97 (CH), 148.63 (CH), 142.39 (C), 139.49 (C), 135.00 (CH), 131.48 (2× CH), 129.00 (2× CH), 123.02 (CH), 122.47 (C), 121.55 (CH), 121.22 (C), 113.06 (CH), 106.90 (CH), 97.47 (CH), 89.87 (C), 76.67 (C), 73.97 (C), 57.13 (CH_2_), 13.58 (CH_3_), 12.51 (CH_2_). HPLC: 100% at R.T. = 4.90 min. HRMS (ESI) calculated (^79^Br) 462.0660 [M + H]^+^, found 462.0703 [M + H]^+^; calculated (^81^Br) 464.0640 [M + H]^+^, found 464.0686 [M + H]^+^.

#### (2,4-Dichlorophenyl)(6-(pent-2-yn-1-yloxy)benzofuran-2-yl)(pyridin-3-yl)methanol (11d: R = 2,4-diCl)

Prepared from (2,4-dichlorophenyl)(6-(pent-2-yn-1-yloxy)benzofuran-2-yl)methanone (9d) (0.17 g, 0.455 mmol). Yield: 0.03 g (15%); *R*_f_ = 0.37 (petroleum ether–EtOAc 1 : 1 v/v). ^1^H NMR (CDCl_3_): *δ* 8.54 (m, 2H, Ar), 7.69 (d, *J* = 8.0 Hz, 1H, Ar), 7.36 (d, *J* = 2.2 Hz, 1H, Ar), 7.32 (d, *J* = 8.6 Hz, 1H, Ar), 7.26 (m, 1H, Ar), 7.15 (dd, *J* = 2.2, 8.5 Hz, 1H, Ar), 7.03 (d, *J* = 2.1 Hz, 1H, Ar), 7.00 (d, *J* = 8.5 Hz, 1H, Ar), 6.87 (dd, *J* = 2.3, 8.6 Hz, 1H, Ar), 6.22 (d, *J* = 0.8 Hz, 1H, Ar), 4.62 (t, *J* = 2.1 Hz, 2H, CH_2_), 4.06 (bs, 1H, OH), 2.19 (qt, *J* = 2.1, 7.5 Hz, 2H, CH_2_), 1.08 (t, *J* = 7.5 Hz, 3H, CH_3_). ^13^C NMR (CDCl_3_): *δ* 156.63 (C), 156.48 (C), 155.96 (C), 149.14 (CH), 148.61 (CH), 138.94 (C), 137.56 (C), 135.29 (C), 134.71 (CH), 133.84 (C), 131.45 (CH), 131.13 (CH), 130.68 (CH), 127.06 (CH), 121.62 (CH), 121.27 (C), 113.17 (CH), 107.10 (CH), 97.55 (CH), 89.89 (C), 76.77 (C, obscured by CDCl3), 73.96 (C), 57.14 (CH_2_), 13.58 (CH_3_), 12.52 (CH_2_). HPLC: 100% at R.T. = 4.92 min. HRMS (ESI) calculated (^35^Cl) 452.0776 [M + H]^+^, found 452.0816 [M + H]^+^; calculated (^37^Cl) 454.0746 [M + H]^+^, found 454.0791 [M + H]^+^.

### Cell culture

JEG-3 cells were purchased from ATCC and grown in Eagle's minimal essential medium (EMEM) supplemented with 10% fetal calf serum (FCS). MCF-10A cells were a gift from Prof. Christopher McCabe (University of Birmingham) and were grown in Dulbecco's modified Eagle medium (DMEM) supplemented with 20 ng mL^−1^ epidermal growth factor (EGF), 100 ng mL^−1^ cholera toxin, 0.01 mg mL^−1^ insulin, 500 ng mL^−1^ hydrocortisone, and 5% horse serum (Sigma). MDA-MB-231 cells were purchased from ATCC and grown in Roswell Park Memorial Institute medium (RPMI1690) supplemented with 10% FCS. All cells were cultured at 37 °C under 5% CO_2_ in a humidified incubator.

### Aromatase inhibition activity

Aromatase activity was assayed using a modified tritiated water assay as previously reported.^12^ JEG-3 cells were grown in 1 mL EMEM to approximately 80% confluence in six-well cell culture plates. Androst-4-ene-3,17-dione[1β-^3^H] was dissolved in serum-free cell culture medium and added into each well. Aromatase activity was measured in the absence and presence of inhibitor (0.001–10 nM). After a 1 h incubation at 37 °C followed by a 5 min incubation on ice, 500 μL of culture medium was taken from each well. Medium was vortexed with 2% dextran-treated charcoal (Sigma-Aldrich) in PBS and centrifuged at 4000 rpm. The supernatant containing the product, [^3^H] H_2_O, was quantified by scintillation counting. Cell protein concentrations were determined using Pierce BCA assay kit (Thermo Fisher Scientific). Aromatase activity results were determined as a concentration of product formed per mg of protein per hour (pmol mg^−1^ h^−1^). Results were shown as a % change in activity compared to control. Each data point was measured in triplicates and the error in the IC_50_ calculations represented as 95% confidence interval.

### BrdU-based cell proliferation assay to assess drug cytotoxicity

MCF-10A and MDA-MB-231 cells were plated onto 96-well microtiter tissue culture plates in RPMI1690 medium at a density of 8 × 10^3^ cells per well (for MCF-10A) or 5 × 10^3^ cells per well (for MDA-MB-231. Groups were treated with either DMSO alone (at no greater than 0.01%) as a vehicle control, or at a dose of 1 μM of inhibitor or doxorubicin control, for 48 h. Effects of drug treatment on cell growth were detected using the BrdU cell proliferation assay (Roche) according to the manufacturer's recommendations. The BrdU colorimetric immunoassay is a quantitative cell proliferation assay based on the measurement of BrdU incorporation during DNA synthesis. After treatments 20 μL per well of BrdU were added to each well, followed by an incubation of 2 h at 37 °C. The cells were subsequently fixed, and the DNA denatured. Anti-BrdU–peroxidase immune complexes were detected by substrate reaction and quantified in an ELISA reader at 370 nm.

### Computational studies

The crystal structure of human placental aromatase (CYP19A1) refined at 2.75 Å (PDB 3S79),^14^ was downloaded from the protein data bank (https://www.rcsb.org). Missing hydrogens were added, and the charge and geometry of the iron atom were adjusted as previously described.^10^ Using the site finder tool in molecular operating environment (MOE) 2015-10 software,^15^ the active site was chosen to contain the main amino acid residues and the haem molecule. The amino acids constituting the wall of the active site contained Arg115, Ile133, Phe134, Phe221, Trp224, Ile305, Ala306, Asp309, Thr310, Val370, Leu372, Val373, Met374, Leu477, Ser478. The 3D structures of the ligands (*R*- and *S*-enantiomers) were generated using MOE builder, energy minimised and saved in a dataset ready for docking studies. The complexes for molecular dynamics (MD) studies were prepared by docking the compounds using MOE.

Molecular dynamics simulations were performed using Schrödinger 2020-1 Desmond programme^16,17^ as previously described.^10^ Briefly, using the pdb files containing the selected docking poses, the structures were optimised with protein preparation wizard. The volume of space in which the simulation takes place, the global cell, is built up by regular 3D simulation boxes. The orthorhombic water box allowed for a 10 Å buffer region between protein atoms and box sides. Overlapping water molecules were deleted, and the systems were neutralised with Na^+^ ions and salt concentration 0.15 M. Molecular dynamics (200 ns simulations) were performed using OPLS_2005 forcefield at 300 K and constant pressure (1 bar).

## Author contributions

Research concept designed by CS, PAF and JG. AGE performed the chemistry and analysis of all compounds supervised by CS. PAF and LEP, supervised by PAF, performed the cell proliferation and aromatase inhibition assays. Computational studies and visualisation performed by CS. Manuscript reviewed by all authors.

## Conflicts of interest

There are no conflicts to declare.

## Supplementary Material

MD-014-D2MD00352J-s001
