# Supplementary material for: Pyridine based dual binding site aromatase (CYP19A1) inhibitors
Source: RSC Med Chem. 2023 Jan 3;14(2):356–66. doi: 10.1039/d2md00352j (PMC9945863; doi:10.1039/d2md00352j)
Supplement: MD-014-D2MD00352J-s001 [file MD-014-D2MD00352J-s001.pdf]

Electronic Supplementary Material (ESI) for RSC Medicinal Chemistry.
This journal is © The Royal Society of Chemistry 2022

SUPPORTING INFORMATION

Pyridine based dual binding site aromatase (CYP19A1) inhibitors

Ahmed G. Eissa,[a,†] Lauren E. Powell,[b] Julia Gee,[a] Paul A. Foster[b,c] and Claire Simons *[a]

Contents

Chemical synthesis and analysis of intermediates **3**, **7**-**9**	**S2-S4**
HPLC for compounds **4**, **10** and **11**	**S5-S8**
^{1}H and ^{13}C NMR for compounds **4**, **10** and **11**	**S9-S21**
**Figure S1**. Positioning of the enantiomers of methoxy derivatives (**4**) in the haem active site of CYP19A1 and 2D ligand interaction image of final frame after MD simulation.	**S22-S23**
**Figure S2**. Positioning of the enantiomers of exemplar but-2-ynyloxy derivatives (**10**) in the haem active site of CYP19A1 and access channels and 2D ligand interaction image of final frame after MD simulation. Front door access channel lined by Arg192, Asp309, Pro481, His480 and Glu483 while an alternative access channel is lined by Phe221, Trp224, Gln225 and Leu477.	**S24-S25**
**Figure S3**. Positioning of the enantiomers of exemplar pent-2-ynyloxy derivatives (**11**) in the haem active site of CYP19A1 and access channels and 2D ligand interaction image of final frame after MD simulation. Front door access channel lined by Arg192, Asp309, Pro481, His480 and Glu483 while an alternative access channel is lined by Phe221, Trp224, Gln225 and Leu477.	**S26-S27**

Experimental

General

All chemicals, reagents and solvents were purchased from Sigma-Aldrich, Alfa Aesar, VWR, Acros and Fluka. Solvents were dried prior to use over molecular sieves (4 Å). For column chromatography, a glass column was slurry packed in the appropriate eluent with silica gel (Fluka Kieselgel 60). TLC was performed on pre-coated silica plates (dimension 20 x 20 cm) (ALUGRAM® SIL G/UV_{254}) with visualisation via UV light (254 nm). Melting points were determined on an electrothermal instrument (Gallenkamp) and were uncorrected. ^{1}H, ^{13}C, and ^{19}F NMR spectra were recorded on a Bruker Advance DP500 spectrometer operating at 500, 125 and 470 MHz, respectively. Chemical shifts are given in parts per million (ppm) relative to the internal standard tetramethylsilane (Me_4Si). Elemental analysis was performed by MEDAC Ltd (Chobham, Surrey, UK) and HPLC-HRMS was performed at the Department of Pharmacy & Pharmacology, University of Bath, Bath, UK. on a Zorbax Eclipse Plus C18 Rapid Resolution 2.1 x 50 mm, 1.8 µm particle size using a 7.5-minute gradient method 5:95 v/v water: methanol with 0.1% formic acid as additive. Ketones **3a**-**c**, **6a**-**b** and **7b**-**7c** were prepared as previously described.[1,2]

General Method for the Preparation of the Ketones 3 and 6. To a solution of salicylaldehyde derivatives (**1** or **5**) (1 m.eq.) in dry CH_3CN (3 mL/mmol of salicylaldehyde) was added K_2CO_3 (2.2 m.eq.) and 2-bromo acetophenone derivatives (**2**) (1 m.eq.). The reaction mixture was stirred at 70 °C for 3 h. The solvent was then evaporated under reduced pressure and the residue dissolved in EtOAc (100 mL) and washed with H_2O (3 x 50 mL). The organic layer was dried ($MgSO_4$) and concentrated under reduced pressure to afford the required ketone **3** or **6**.

(2,4-Dichlorophenyl)(6-methoxybenzofuran-2-yl)methanone (**3d**: R = 2,4-diCl). Prepared using 2-hydroxy-4-methoxybenzaldehyde (**1**) (0.5 g, 3.28 mmol) and (2-bromo-2′,4′-dichloroacetophenone (**2d**) (0.87 g, 3.28 mmol) to afford the product (**3d**) as a light brown solid. Yield: 0.93 g (88%); m.p. 156-158 °C; R_f = 0.6 (petroleum ether - EtOAc 3:1 v/v). ^{1}H NMR ($CDCl_3$): δ 7.57 (d, *J* = 8.7 Hz, 1H, Ar), 7.54 (d, *J* = 1.9 Hz, 1H, Ar), 7.50 (d, *J* = 8.2 Hz, 1H, Ar), 7.40 (dd, *J* = 1.9, 8.2 Hz, 1H, Ar), 7.30 (d, *J* = 0.9 Hz, 1H, Ar), 7.08 (d, *J* = 1.8 Hz, 1H, Ar), 6.99 (dd, *J* = 2.4, 8.7 Hz, 1H, Ar), 3.90 (s, 3H, OCH_3). ^{13}C NMR ($CDCl_3$): δ 182.00 (C), 161.85 (C), 158.24 (C), 151.37 (C), 137.13 (C), 136.00 (C), 132.90 (C), 130.28 (2 x CH), 127.02 (CH), 123.95 (CH), 120.30 (C), 118.52 (CH), 115.04 (CH), 95.55 (CH), 55.80 (CH_3). HRMS (ESI) m/z: Calculated (^{35}Cl) 321.0086 $[M+H]^+$, Found 321.0079 $[M+H]^+$; Calculated (^{37}Cl) 321.0056 $[M+H]^+$, Found 323.0054 $[M+H]^+$;

(4-Bromophenyl)(6-((tetrahydro-2*H*-pyran-2-yl)oxy)benzofuran-2-yl)methanone (**6c**: R = 4-Br). Prepared using 2-hydroxy-4-((tetrahydro-2*H*-pyran-2-yl)oxy)benzaldehyde (**5**) (0.64 g, 2.88 mmol) and 2,4′-dibromoacetophenone (**2c**) (0.8 g, 2.88 mmol) to afford the product (**6c**) after recrystallisation from EtOH as a white solid. Yield: 1.0 g (86%); m.p. 180-184 °C; R_f = 0.67 (petroleum ether - EtOAc 3:1 v/v). ^{1}H NMR ($CDCl_3$): δ 7.85 (d, *J* = 8.5 Hz, 2H, Ar), 7.60 (d, *J* = 8.5 Hz, 2H, Ar), 7.52 (d, *J* = 8.6 Hz, 1H, Ar), 7.41 (d, *J* = 0.9 Hz, 1H, Ar), 7.26 (s, 1H, Ar), 7.01 (dd, *J* = 2.1, 8.6 Hz, 1H, Ar), 5.42 (t, *J* = 3.2 Hz, 1H, CH-pyran), 3.85 (m, 1H, CH_2-pyran), 3.58 (m, 1H, CH_2-pyran), 1.95 (m, 3H, CH_2-pyran), 1.66 (m, 3H, CH_2-pyran). ^{13}C NMR ($CDCl_3$): δ 182.71 (C), 158.62 (C), 157.47 (C), 151.91 (C), 136.10 (C), 131.82 (2 x CH), 130.96 (2 x CH), 127.80 (C), 123.57 (CH), 121.03 (C), 117.16 (CH), 115.72 (CH), 99.34 (CH), 96.95 (CH), 62.22 (CH_2), 30.24 (CH_2), 25.08 (CH_2), 18.71 (CH_2). HRMS (ESI) Calculated (^{79}Br) 401.0389 $[M+H]^+$, Found 401.0382 $[M+H]^+$; Calculated (^{81}Br) 403.0369 $[M+H]^+$, Found 403.0365, $[M+H]^+$; 425.0188 $[M+Na]^+$, Found 425.0184 $[M+Na]^+$;

(2′,4′-Dichlorophenyl)(6-((tetrahydro-2*H*-pyran-2-yl)oxy)benzofuran-2-yl)methanone (**6d**: R = 2,4-diCl). Prepared using 2-hydroxy-4-((tetrahydro-2*H*-pyran-2-yl)oxy)benzaldehyde (**5**) (0.64 g, 2.88 mmol) and 2-bromo-2′,4′-dichloroacetophenone (**2d**) (0.77 g, 2.88 mmol) to afford the product (**6d**) after recrystallisation from EtOH as a yellow solid. Yield: 0.95 g (85%); m.p. 118-122 °C; R_f = 0.7 (petroleum ether - EtOAc 3:1 v/v). ^{1}H NMR ($CDCl_3$): δ 7.58 (d, *J* = 8.7 Hz, 1H, Ar), 7.54 (d, *J* = 1.9 Hz, 1H, Ar), 7.49 (d, *J* = 8.2 Hz, 1H, Ar), 7.40 (dd, *J* = 1.9, 8.2 Hz, 1H, Ar), 7.33 (d, *J* = 1.9 Hz, 1H, Ar), 7.30 (d, *J* = 0.9 Hz, 1H, Ar), 7.09 (dd, *J* = 2.1, 8.7 Hz, 1H, Ar), 5.51 (t, *J* = 3.2 Hz, 1H, CH-pyran), 3.93 (m, 1H, CH_2-pyran), 3.66 (m, 1H, CH_2-pyran), 2.04 (m, 1H, CH_2-pyran), 1.93 (m, 2H, CH_2- pyran), 1.75 (m, 3H, CH_2-pyran). ^{13}C NMR ($CDCl_3$): δ 182.14 (C), 159.03 (C), 157.95 (C), 151.56 (C), 137.15 (C), 135.98 (C), 132.92 (C), 130.29 (CH), 127.01 (CH), 123.80 (CH), 121.03 (C), 118.34 (CH), 115.88 (CH), 99.35 (CH), 96.91 (CH), 62.19 (CH_2), 30.19 (CH_2), 25.05 (CH2), 18.67 (CH_2).

General Method for the Preparation of the 6-Hydroxybenzofuran-2-yl derivatives 7. To a solution of (6-((tetrahydro-2*H*-pyran-2-yl)oxy)benzofuran-2-yl)(phenyl)methanone (**6**) in dioxane (4 mL), conc. HCl (1 mL) was added and the reaction stirred for 1 h at room temperature. The reaction mixture was then concentrated under reduced pressure and the residue washed with CH_2Cl_2 to afford (6-hydroxybenzofura-2-yl)(phenyl)methanone derivatives (**7**).

(4-Fluorophenyl)(6-hydroxybenzofuran-2-yl)methanone (**7a**: R = 4-F). Prepared using (4′-fluorophenyl)(6-((tetrahydro-2*H*-pyran-2-yl)oxy)benzofuran-2-yl)methanone (**6a**) (0.785 g, 2.31 mmol) to afford the product (**7a**) as a white solid. Yield: 0.39 g (66%); m.p. 196-198 °C; R_f = 0.3 (petroleum ether - EtOAc 3:1 v/v). ^{1}H NMR (DMSO-d_6): δ 10.23 (bs, 1H, OH), 8.06 (dd, *J* = 5.5, 8.8 Hz, 2H, Ar), 7.69 (d, *J* = 0.8 Hz, 1H, Ar), 7.65 (d, *J* = 8.6 Hz, 1H, Ar), 7.43 (t, *J* = 8.8 Hz, 2H, Ar), 7.03 (s, 1H, Ar), 6.91 (dd,

J = 2.1, 8.6 Hz, 1H, Ar). ^{13}C NMR (DMSO-d$_6$): δ 181.78 (C), 166.16 (d, $^1J_{C,F}$ = 250 Hz, C), 159.91 (C), 157.70 (C), 150.84 (C), 134.23 (d, $^4J_{C,F}$ = 3.75 Hz, C), 132.37 (d, $^3J_{C,F}$ = 10 Hz, 2 x CH), 124.89 (CH), 119.49 (C), 118.70 (CH), 116.29 (d, $^2J_{C,F}$ = 21.25 Hz, 2 x CH), 115.17 (CH), 97.95 (CH). HRMS (ESI) Calculated 257.0569 [M+H]$^+$, Found 257.0609 [M+H]$^+$.

(2,4-Dichlorophenyl)(6-hydroxybenzofuran-2-yl)methanone (**7d**: R = 2,4-diCl). Prepared using (2′,4′-dichlorophenyl)(6-((tetrahydro-2*H*-pyran-2-yl)oxy)benzofuran-2-yl)methanone (**6d**) (0.95 g, 2.43 mmol) to afford the product (**7d**) as a yellow solid. Yield: 0.49 g (66%); m.p. 192-194 °C; R_f = 0.35 (petroleum ether - EtOAc 3:1 v/v). ^{1}H NMR (DMSO-d$_6$): δ 10.35 (bs, 1H, OH), 7.83 (d, J = 1.9 Hz, 1H, Ar), 7.72 (d, J = 8.2 Hz, 1H, Ar), 7.62 (m, 2H), 7.51 (d, J = 1.0 Hz, 1H, Ar), 7.02 (m, 1H, Ar), 6.90 (dd, J = 2.0, 8.6 Hz, 1H, Ar). ^{13}C NMR (DMSO-d$_6$): δ 181.40 (C), 160.55 (C), 158.32 (C), 150.67 (C), 136.60 (C), 136.32 (C), 131.95 (C), 131.30 (CH), 130.09 (CH), 128.01 (CH), 125.25 (CH), 120.65 (CH), 119.47 (C), 115.52 (CH), 97.95 (CH). HRMS (ESI) Calculated (^{35}Cl) 306.9929 [M+H]$^+$, Found 306.9924 [M+H]$^+$, Calculated (^{37}Cl) 308.9900 [M+H]$^+$, Found 308.9897 [M+H]$^+$.

General Method for the Preparation of the 6-*O*-but-2-ynyl (8) and 6-*O*-pent-2-ynyl (9) derivatives. To a solution of (6-hydroxybenzofura-2-yl)(phenyl)methanone derivatives (**7**) (1 m.eq.) in dry CH_3CN (10 mL/mmol), K_2CO_3 (2.2 m.eq.) was added and the mixture stirred for 1 h at 40 °C then 1-bromoalk-2-yne (2 m.eq.) was added and the reaction mixture stirred at room temperature for 16 h. The reaction mixture was concentrated under reduced pressure and the residue dissolved in EtOAc (100 mL). The organic layer was washed with H_2O (3 x 50 mL), dried ($MgSO_4$) and concentrated under reduced pressure. Purification by gradient column chromatography afforded (6-(but-2-yn-1-yloxy)benzofuran-2-yl)(phenyl)methanone derivatives (**8**) and (6-(pent-2-yn-1-yloxy)benzofuran-2-yl)(phenyl)methanone derivatives (**9**) at 20% EtOAc in petroleum ether (v/v).

(6-(But-2-yn-1-yloxy)benzofuran-2-yl)(4-fluorophenyl)methanone (**8a**: R = 4-F). Prepared using (4-fluorophenyl)(6-hydroxybenzofuran-2-yl)methanone (**7a**) (0.15 g, 0.58 mmol) and 1-bromobut-2-yne (0.076 mL, 0.87 mmol) to afford the product (**8a**) as a white solid. Yield: 0.11 g (61%); m.p. 162-164 °C; R_f = 0.6 (petroleum ether - EtOAc 3:1 v/v). ^{1}H NMR ($CDCl_3$): δ 8.12 (m, 2H, Ar), 7.62 (d, J = 8.7 Hz, 1H, Ar), 7.50 (d, J = 0.9 Hz, 1H, Ar), 7.28 (m, 3H, Ar), 7.04 (dd, J = 2.2, 8.7 Hz, 1H, Ar), 4.76 (q, J = 2.3 Hz, 2H, CH_2), 1.90 (t, J = 2.3 Hz, 3H, CH_3). ^{13}C NMR ($CDCl_3$): δ 182.28 (C), 166.56 (d, $^1J_{C,F}$ = 252.5 Hz, C), 159.35 (C), 157.38 (C), 151.90 (C), 133.68 (d, $^4J_{C,F}$ = 3.75 Hz, C), 131.99 (d, $^3J_{C,F}$ = 8.75 Hz, 2 x CH), 123.67 (CH), 120.73 (C), 117.01 (CH), 115.79 (d, $^2J_{C,F}$ = 21.25 Hz, 2 x CH), 115.12 (CH), 97.06 (CH), 84.60 (C), 73.34 (C), 57.98 (CH_2), 3.72 (CH_3). HRMS (ESI) Calculated 309.0926 [M+H]$^+$, Found 309.0919 [M+H]$^+$.

(6-(But-2-yn-1-yloxy)benzofuran-2-yl)(4-chlorophenyl)methanone (**8b**: R = 4-Cl). Prepared using (4-chlorophenyl)(6-hydroxybenzofuran-2-yl)methanone (**7b**) (0.39 g, 1.43 mmol) and 1-bromobut-2-yne (0.187 mL, 2.15 mmol) to afford the product (**8b**) as a white solid. Yield: 0.16 g (34%); m.p. 132-134 °C; R_f = 0.7 (petroleum ether - EtOAc 3:1 v/v). ^{1}H NMR ($CDCl_3$): δ 7.90 (d, J = 8.6 Hz, 2H, Ar), 7.52 (d, J = 8.7 Hz, 1H, Ar), 7.43 (d, J = 8.6 Hz, 2H, Ar), 7.39 (d, J = 0.9 Hz, 1H, Ar), 7.14 (d, J = 1.8 Hz, 1H, Ar), 6.94 (dd, J = 2.2, 8.7 Hz, 1H, Ar), 4.66 (q, J = 2.3 Hz, 2H, CH_2), 1.79 (t, J = 2.3 Hz, 3H, CH_3). ^{13}C NMR ($CDCl_3$): δ 182.47 (C), 159.44 (C), 157.43 (C), 151.78 (C), 139.13 (C), 135.71 (C), 130.76 (2 x CH), 128.85 (2 x CH), 123.72 (CH), 120.70 (C), 117.20 (CH), 115.18 (CH), 97.03 (CH), 84.62 (C), 73.33 (C), 56.98 (CH_2), 3.72 (CH_3). HRMS (ESI) Calculated (^{35}Cl) 325.0631 [M+H]$^+$, Found 325.0623 [M+H]$^+$; Calculated (^{37}Cl) 327.0603 [M+H]$^+$, Found 327.0600 [M+H]$^+$.

(6-(But-2-yn-1-yloxy)benzofuran-2-yl)(4-bromophenyl)methanone (**8c**: R = 4-Br). Prepared using (4-bromophenyl)(6-hydroxybenzofuran-2-yl)methanone (**7c**) (0.20 g, 0.63 mmol) and 1-bromobut-2-yne (0.08 mL, 0.94 mmol) to afford the product (**8c**) as a white solid. Yield: 0.10 g (43%); m.p. 143-145 °C; R_f = 0.37 (petroleum ether - EtOAc 3:1 v/v). ^{1}H NMR ($CDCl_3$): δ 7.84 (d, J = 8.6 Hz, 2H, Ar), 7.61 (d, J = 8.6 Hz, 2H, Ar), 7.53 (d, J = 8.7 Hz, 1H, Ar), 7.40 (d, J = 0.9 Hz, 1H, Ar), 7.16 (d, J = 1.8 Hz, 1H, Ar), 6.95 (dd, J = 2.2, 8.7 Hz, 1H, Ar), 4.67 (q, J = 2.3 Hz, 2H, CH_2), 1.81 (t, J = 2.3 Hz, 3H, CH_3). ^{13}C NMR ($CDCl_3$): δ 182.48 (C), 159.46 (C), 157.41 (C), 151.76 (C), 136.16 (C), 131.84 (2 x CH), 130.87 (2 x CH), 127.64 (C), 123.73 (CH), 120.71 (C), 117.26 (CH), 115.21 (CH), 97.05 (CH), 84.63 (C), 73.30 (C), 56.99 (CH_2), 3.72 (CH_3). HRMS (ESI) Calculated (^{79}Br) 369.0129 [M+H]$^+$, Found 369.0119 [M+H]$^+$; Calculated (^{81}Br) 371.0107 [M+H]$^+$, Found 371.0101 [M+H]$^+$.

(6-(But-2-yn-1-yloxy)benzofuran-2-yl)(2,4-dichlophenyl)methanone (**8d**: R = 2,4-diCl). Prepared using (2,4-dichlorophenyl)(6-hydroxybenzofuran-2-yl)methanone (**7d**) (0.20 g, 0.65 mmol) and 1-bromobut-2-yne (0.085 mL, 0.97 mmol) to afford the product (**8d**) as a yellow oil. Yield: 0.20 g (86%); R_f = 0.32 (petroleum ether - EtOAc 3:1 v/v). ^{1}H NMR ($CDCl_3$): δ 7.49 (d, J = 8.7 Hz, 1H, Ar), 7.45 (d, J = 1.9 Hz, 1H, Ar), 7.40 (d, J = 8.2 Hz, 1H, Ar), 7.32 (dd, J = 1.9, 8.2 Hz, 1H, Ar), 7.20 (d, J = 0.9 Hz, 1H, Ar), 7.13 (d, J = 1.9 Hz, 1H, Ar), 6.93 (dd, J = 2.2, 8.7 Hz, 1H, Ar), 4.66 (q, J = 2.3 Hz, 2H, CH_2), 1.80 (t, J = 2.3 Hz, 3H, CH_3). ^{13}C NMR ($CDCl_3$): δ 182.03 (C), 159.86 (C), 157.95 (C), 151.54 (C), 137.18 (C), 135.94 (C), 132.91 (C), 130.31 (CH), 130.28 (CH), 127.03 (CH), 123.96 (CH), 120.70 (C), 118.50 (CH), 115.47 (CH), 97.01 (CH), 84.72 (C), 73.20 (C), 56.99 (CH_2), 3.71 (CH_3). HRMS (ESI) Calculated (^{35}Cl) 359.0242 [M+H]$^+$, Found 359.0238 [M+H]$^+$; Calculated (^{37}Cl) 361.0213 [M+H]$^+$, Found 361.0210 [M+H]$^+$.

(4-Fluorophenyl)(6-(pent-2-yn-1-yloxy)benzofuran-2-yl)methanone (**9a**: R = 4-F). Prepared using (4-fluorophenyl)(6-hydroxybenzofuran-2-yl)methanone (**7a**) (0.20 g, 0.78 mmol) and 1-bromopent-2-yne (0.119 mL, 1.17 mmol) to afford the product (**9a**) as a white solid. Yield: 0.19 g (76%); m.p. 106-108 °C; R_f = 0.62 (petroleum ether - EtOAc 3:1 v/v). ^{1}H NMR

(CDCl$_3$): δ 8.01 (dd, *J* = 5.4, 8.9 Hz, 2H, Ar), 7.52 (d, *J* = 8.7 Hz, 1H, Ar), 7.40 (d, *J* = 0.9 Hz, 1H, Ar), 7.15 (m, 3H, Ar), 6.95 (dd, *J* = 2.2, 8.6 Hz, 1H, Ar), 4.68 (t, *J* = 2.1 Hz, 2H, CH$_2$), 2.20 (qt, *J* = 2.1, 7.5 Hz, 2H, CH$_2$), 1.08 (t, *J* = 7.5 Hz, 3H, CH$_3$). ^{13}C NMR (CDCl$_3$): δ 182.29 (C), 166.55 (d, $^1J_{C,F}$ = 252.5 Hz, C), 159.40 (C), 157.38 (C), 151.88 (C), 133.69 (d, $^4J_{C,F}$ = 3.75 Hz, C), 131.98 (d, $^3J_{C,F}$ = 8.75 Hz, 2 x CH), 123.64 (CH), 120.72 (C), 117.03 (CH), 115.78 (d, $^2J_{C,F}$ = 21.25 Hz, 2 x CH), 115.14 (CH), 97.07 (CH), 90.35 (C), 73.49 (C), 57.05 (CH$_2$), 13.56 (CH$_3$), 12.50 (CH$_2$). HRMS (ESI) Calculated 323.1083 [M+H]$^+$, Found 323.1077 [M+H]$^+$.

(4-Chlorophenyl)(6-(pent-2-yn-1-yloxy)benzofuran-2-yl)methanone (**9b**: R = 4-Cl). Prepared using (4-chlorophenyl)(6-hydroxybenzofuran-2-yl)methanone (**7b**) (0.39 g, 1.43 mmol) and 1-bromopent-2-yne (0.29 mL, 2.86 mmol) to afford the product (**9b**) as a white solid. Yield: 0.17 g (35%); m.p. 106-110 °C; R_f = 0.72 (petroleum ether - EtOAc 3:1 v/v). ^{1}H NMR (CDCl$_3$): δ 8.01 (d, *J* = 8.6 Hz, 2H, Ar), 7.62 (d, *J* = 8.7 Hz, 1H, Ar), 7.54 (d, *J* = 8.6 Hz, 2H, Ar), 7.49 (d, *J* = 0.9 Hz, 1H, Ar), 7.25 (d, *J* = 1.8 Hz, 1H, Ar), 7.05 (dd, *J* = 2.2, 8.7 Hz, 1H, Ar), 4.78 (t, *J* = 2.1 Hz, 2H, CH$_2$), 2.29 (qt, *J* = 2.1, 7.5 Hz, 2H, CH$_2$), 1.18 (t, *J* = 7.5 Hz, 3H, CH$_3$). ^{13}C NMR (CDCl$_3$): δ 182.52 (C), 159.50 (C), 157.45 (C), 151.78 (C), 139.14 (C), 135.74 (C), 130.77 (2 x CH), 128.86 (2 x CH), 123.70 (CH), 120.70 (C), 117.24 (CH), 115.22 (CH), 97.07 (CH), 90.39 (C), 73.47 (C), 57.06 (CH$_2$), 13.56 (CH$_3$), 12.51 (CH$_2$). HRMS (ESI) Calculated (^{35}Cl) 339.0787 [M+H]$^+$, Found 339.0784 [M+H]$^+$; Calculated (^{37}Cl) 341.0759 [M+H]$^+$, Found 341.0763[M+H]$^+$.

(4-Bromophenyl)(6-(pent-2-yn-1-yloxy)benzofuran-2-yl)methanone (**9c**: R = 4-Br). Prepared using (4-bromophenyl)(6-hydroxybenzofuran-2-yl)methanone (**7c**) (0.20 g, 0.63 mmol) and 1-bromopent-2-yne (0.096 mL, 0.94 mmol) to afford the product (**9c**) as a white solid. Yield: 0.10 g (40%); m.p. 116-118 °C; R_f = 0.28 (petroleum ether - EtOAc 3:1 v/v). ^{1}H NMR (CDCl$_3$): δ 7.84 (d, *J* = 8.6 Hz, 2H, Ar), 7.61 (d, *J* = 8.6 Hz, 2H, Ar), 7.53 (d, *J* = 8.7 Hz, 1H, Ar), 7.40 (d, *J* = 0.9 Hz, 1H, Ar), 7.16 (d, *J* = 1.8 Hz, 1H, Ar), 6.95 (dd, *J* = 2.2, 8.7 Hz, 1H, Ar), 4.69 (t, *J* = 2.1 Hz, 2H, CH$_2$), 2.20 (qt, *J* = 2.1, 7.5 Hz, 2H, CH$_2$), 1.09 (t, *J* = 7.5 Hz, 3H, CH$_3$). ^{13}C NMR (CDCl$_3$): δ 182.68 (C), 159.51 (C), 157.46 (C), 151.76 (C), 136.18 (C), 131.84 (2 x CH), 130.87 (2 x CH), 127.75 (C), 123.70 (CH), 120.70 (C), 117.29 (CH), 115.24 (CH), 97.06 (CH), 90.39 (C), 73.46 (C), 57.06 (CH$_2$), 13.56 (CH$_3$), 12.50 (CH$_2$). HRMS (ESI) Calculated (^{79}Br) 383.0282 [M+H]$^+$, Found 369.0119 [M+H]$^+$; Calculated (^{81}Br) 385.0263 [M+H]$^+$, Found 385.0259 [M+H]$^+$.

(2,4-Dichlorophenyl)(6-(pent-2-yn-1-yloxy)benzofuran-2-yl)methanone (**9d**: R = 2,4-diCl). Prepared using (2,4-dichlorophenyl)(6-hydroxybenzofuran-2-yl)methanone (**7d**) (0.20 g, 0.65 mmol) and 1-bromopent-2-yne (0.099 mL, 0.97 mmol) to afford the product (**9d**) as a yellow oil. Yield: 0.18 g (75%); R_f = 0.25 (petroleum ether - EtOAc 3:1 v/v). ^{1}H NMR (CDCl$_3$): δ 7.49 (d, *J* = 8.8 Hz, 1H, Ar), 7.45 (d, *J* = 1.9 Hz, 1H, Ar), 7.40 (d, *J* = 8.2 Hz, 1H, Ar), 7.31 (dd, *J* = 1.9, 8.2 Hz, 1H, Ar), 7.20 (d, *J* = 0.9 Hz, 1H, Ar), 7.12 (d, *J* = 1.8 Hz, 1H, Ar), 6.94 (dd, *J* = 2.2, 8.8 Hz, 1H, Ar), 4.68 (t, *J* = 2.1 Hz, 2H, CH$_2$), 2.19 (qt, *J* = 2.1, 7.5 Hz, 2H, CH$_2$), 1.08 (t, *J* = 7.5 Hz, 3H, CH$_3$). ^{13}C NMR (CDCl$_3$): δ 182.05 (C), 159.51 (C), 157.96 (C), 151.47 (C), 137.18 (C), 135.95 (C), 132.91 (C), 130.31 (CH), 130.28 (CH), 127.03 (CH), 123.94 (CH), 120.69 (C), 118.53 (CH), 115.50 (CH), 97.02 (CH), 90.47 (C), 73.35 (C), 57.06 (CH$_2$), 13.55 (CH$_3$), 12.49 (CH$_2$). HRMS (ESI) Calculated (^{35}Cl) 373.0398 [M+H]$^+$, Found 373.0394 [M+H]$^+$; Calculated (^{37}Cl) 375.0369 [M+H]$^+$, Found 375.0367 [M+H]$^+$.

References

1 M.R. Saberi, T.K. Vinh, S.W. Yee, B.J.N. Griffiths, P.J. Evans and C. Simons, *J. Med. Chem*., 2006, **49**, 1016-1022.

2 H.M. Meshram, B.C. Reddy, B.R.V. Prasad, P.R. Goud, G.S. Kumar, R.N. Kumar, *Synth. Commun*., 2012, **42**,1669–1676.

HPLC Purity Analysis for compounds **4**, **10** and **11**

4a

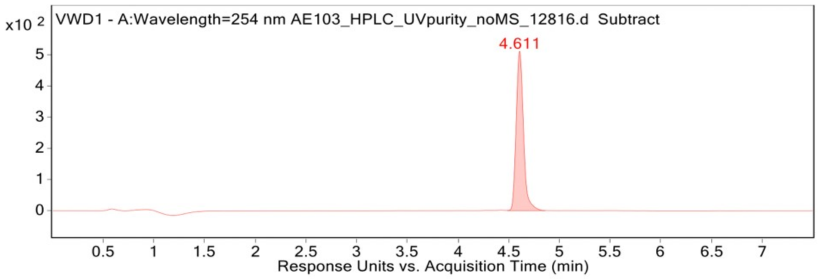

Figure: Base peak or HPLC chromatogram (indicated in left hand corner)

User Chromatogram Peak List

RT (min)	Area	Area %	Area Sum (%)	Symmetry	Width (min)
4.61	2586.59	100.00	100.00	1.13	0.367

4b

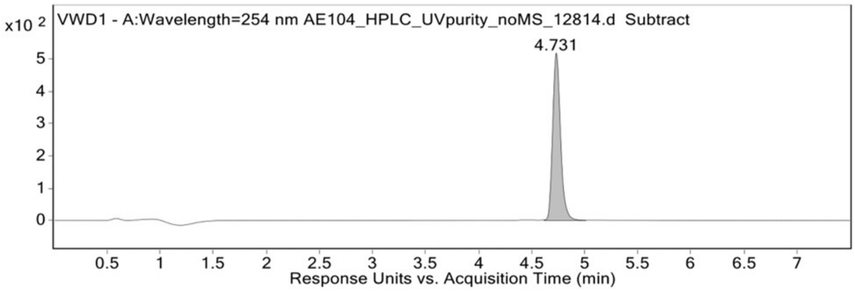

Figure: Base peak or HPLC chromatogram (indicated in left hand corner)

User Chromatogram Peak List

RT (min)	Area	Area %	Area Sum (%)	Symmetry	Width (min)
4.73	2695.78	100.00	100.00	1.38	0.393

4c

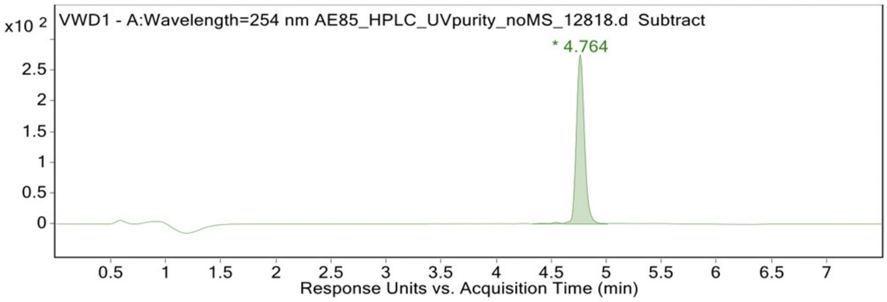

Figure: Base peak or HPLC chromatogram (indicated in left hand corner)

User Chromatogram Peak List

RT (min)	Area	Area %	Area Sum (%)	Symmetry	Width (min)
4.54	15.31	1.08	1.07	0.92	0.273
4.76	1415.62	100.00	98.93	1.21	0.393

4d

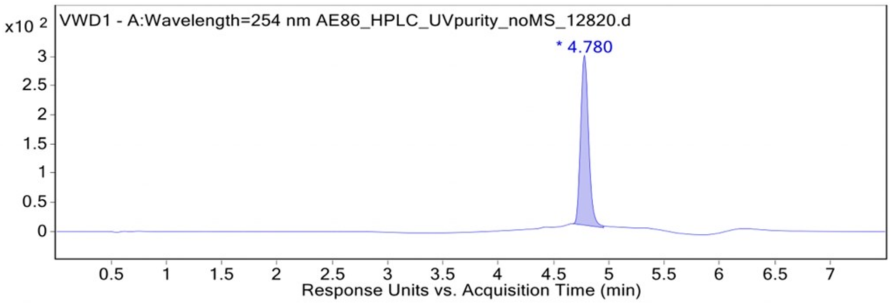

Figure: Base peak or HPLC chromatogram (indicated in left hand corner)

User Chromatogram Peak List

RT (min)	Area	Area %	Area Sum (%)	Symmetry	Width (min)
4.78	1467.49	100.00	100.00	1.21	0.280

10a

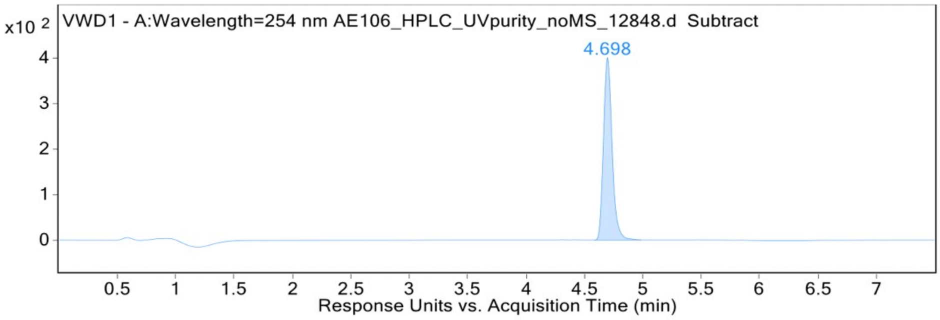

Figure: Base peak or HPLC chromatogram (indicated in left hand corner)

User Chromatogram Peak List

RT (min)	Area	Area %	Area Sum (%)	Symmetry	Width (min)
4.70	2072.18	100.00	100.00	1.33	0.400

10b

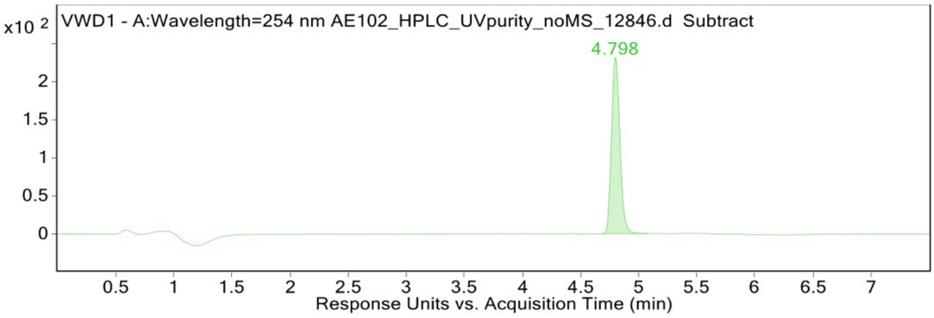

Figure: Base peak or HPLC chromatogram (indicated in left hand corner)

User Chromatogram Peak List

RT (min)	Area	Area %	Area Sum (%)	Symmetry	Width (min)
4.80	1166.36	100.00	100.00	1.33	0.400

10c

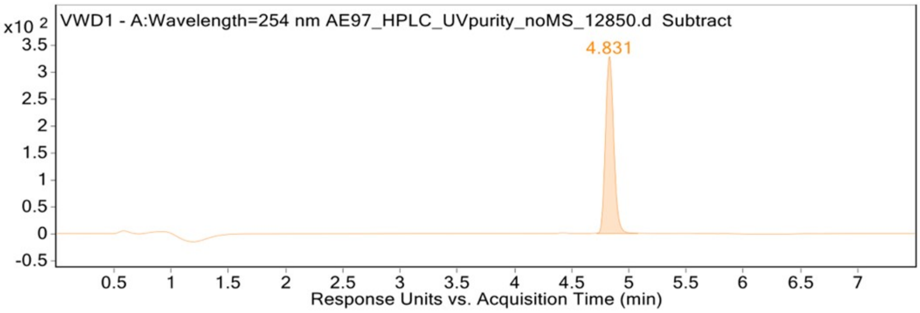

Figure: Base peak or HPLC chromatogram (indicated in left hand corner)

User Chromatogram Peak List

RT (min)	Area	Area %	Area Sum (%)	Symmetry	Width (min)
4.83	1656.23	100.00	100.00	1.17	0.360

10d

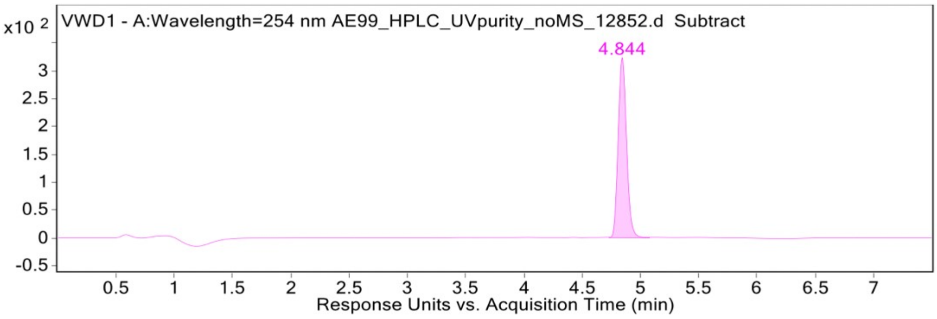

Figure: Base peak or HPLC chromatogram (indicated in left hand corner)

User Chromatogram Peak List

RT (min)	Area	Area %	Area Sum (%)	Symmetry	Width (min)
4.84	1630.82	100.00	100.00	1.17	0.347

11a

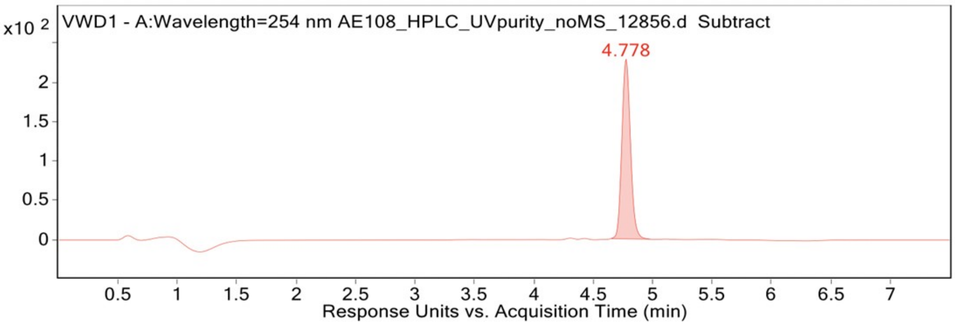

Figure: Base peak or HPLC chromatogram (indicated in left hand corner)

User Chromatogram Peak List

RT (min)	Area	Area %	Area Sum (%)	Symmetry	Width (min)
4.78	1155.05	100.00	100.00	1.13	0.320

11b

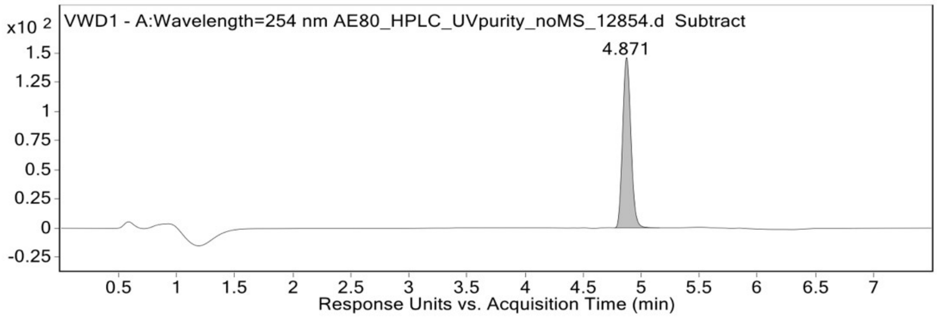

Figure: Base peak or HPLC chromatogram (indicated in left hand corner)

User Chromatogram Peak List

RT (min)	Area	Area %	Area Sum (%)	Symmetry	Width (min)
4.87	723.82	100.00	100.00	1.3	0.387

11c

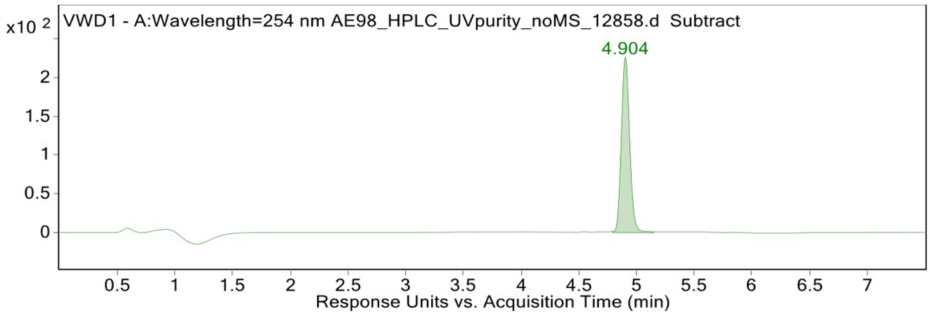

Figure: Base peak or HPLC chromatogram (indicated in left hand corner)

User Chromatogram Peak List

RT (min)	Area	Area %	Area Sum (%)	Symmetry	Width (min)
4.90	1142.6	100.00	100.00	1.14	0.360

11d

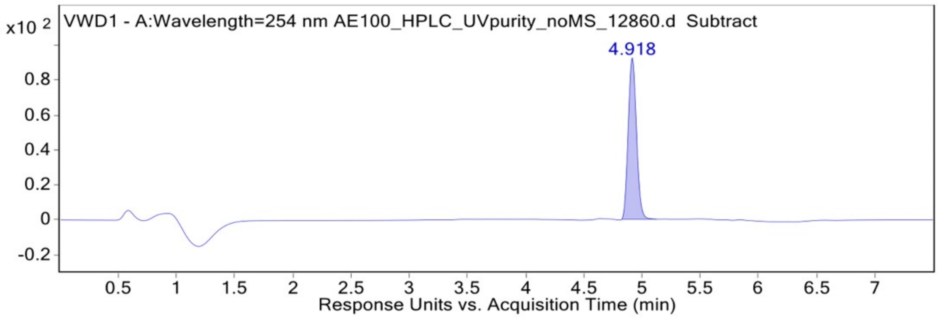

Figure: Base peak or HPLC chromatogram (indicated in left hand corner)

User Chromatogram Peak List

RT (min)	Area	Area %	Area Sum (%)	Symmetry	Width (min)
4.92	456.19	100.00	100.00	1.1	0.298

^{1}H and ^{13}C NMR for compounds **4**, **10** and **11**

4a (DMSO-d_6)

^{1}H NMR:

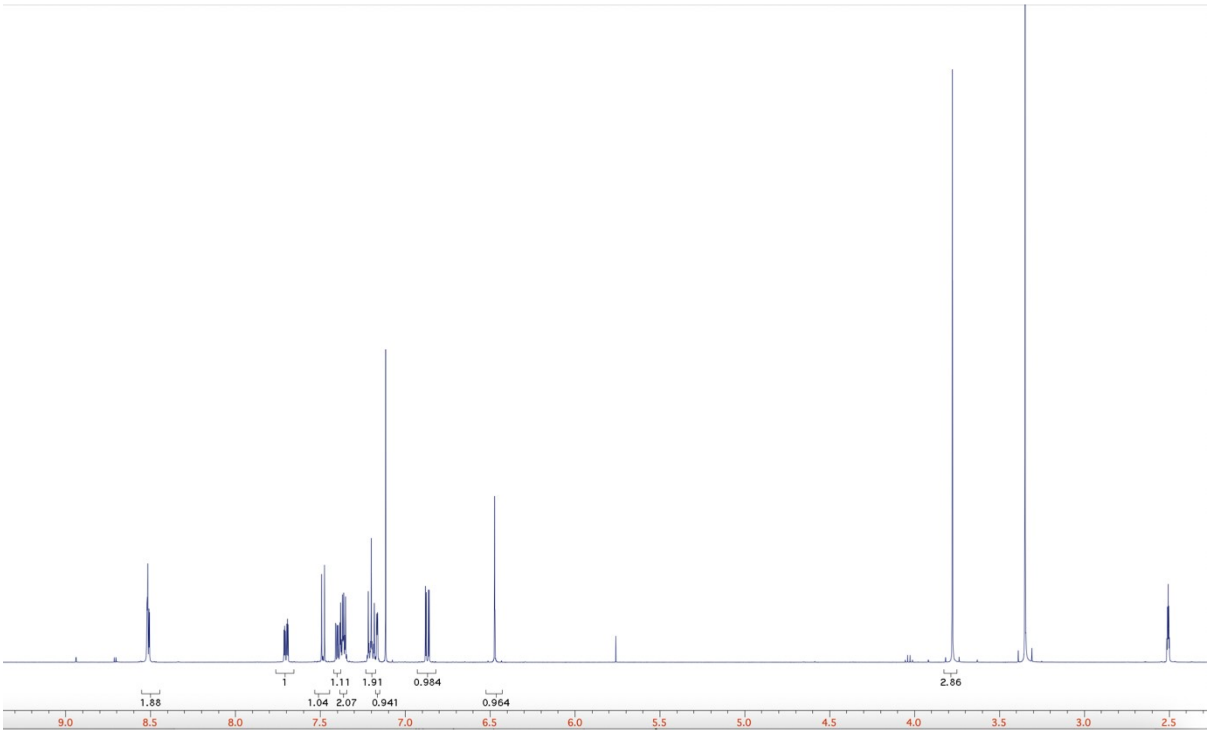

^{13}C NMR:

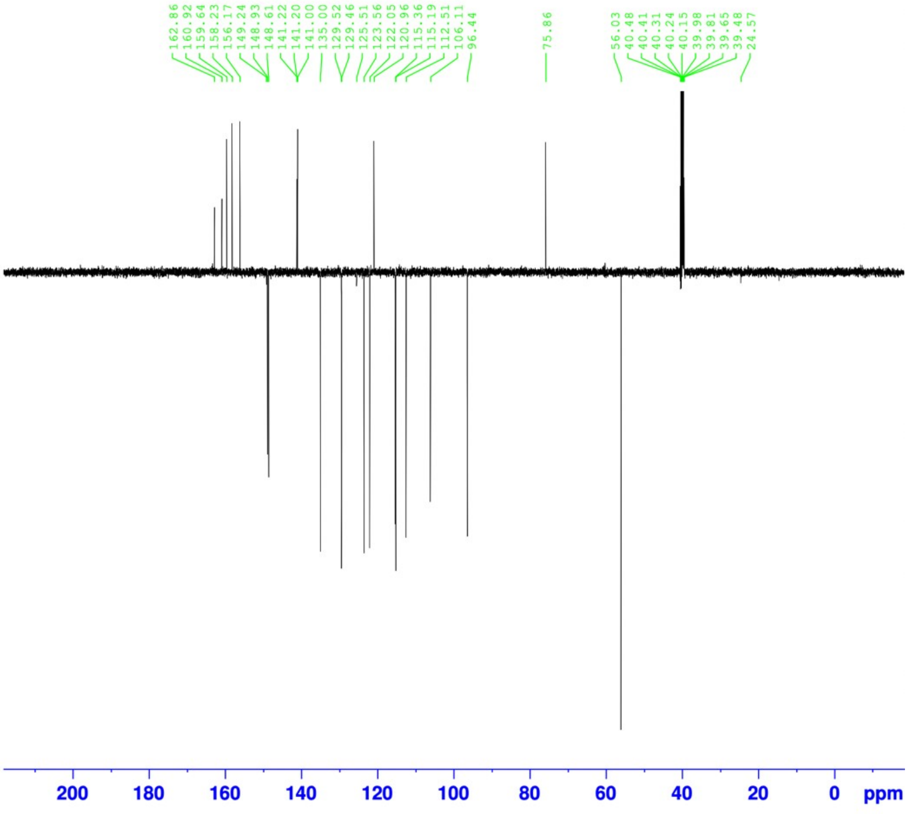

4b ($CDCl_3$)

^1H NMR:

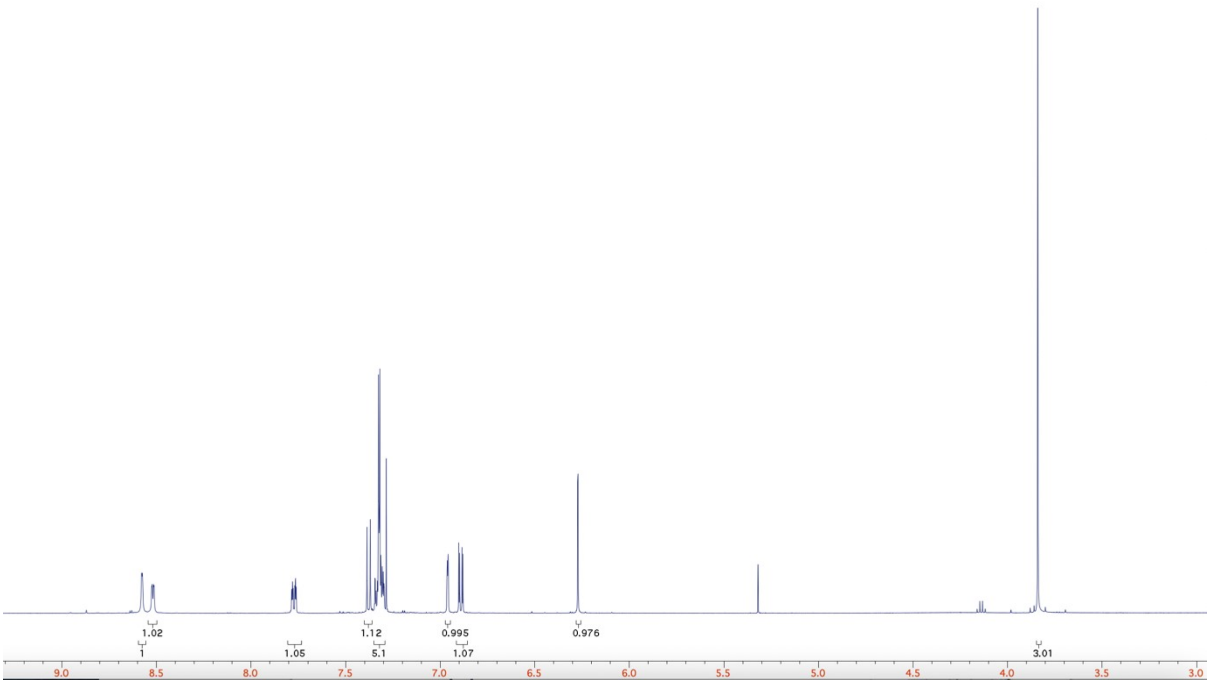

^{13}C NMR:

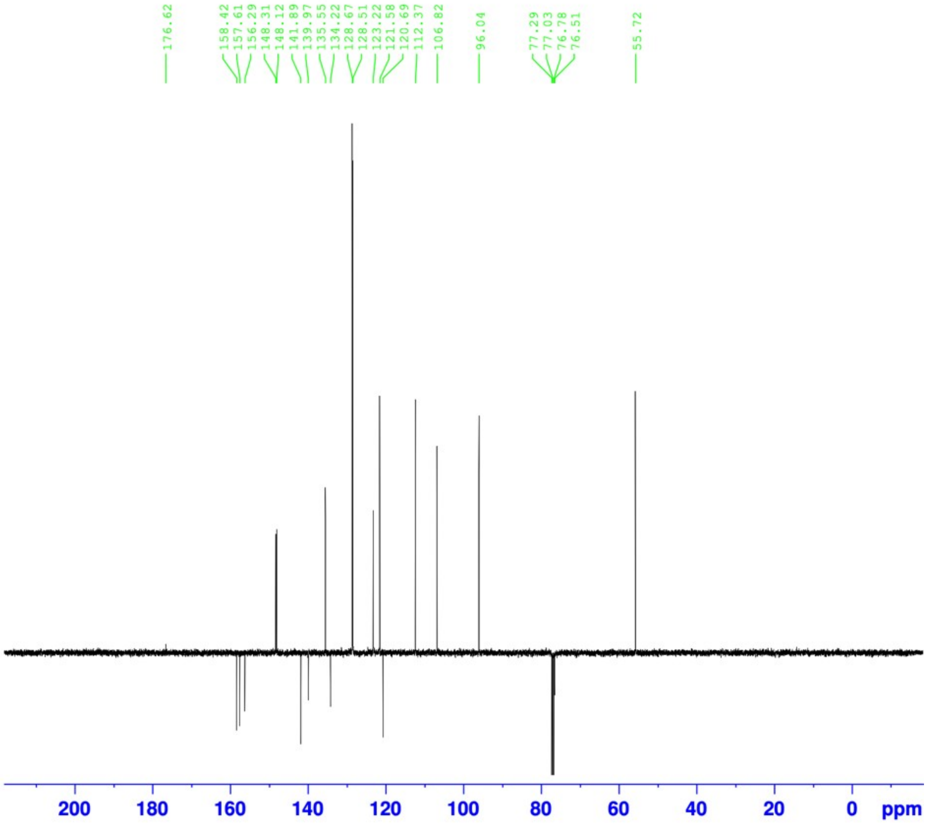

4c (DMSO-d_6)

^{1}H NMR:

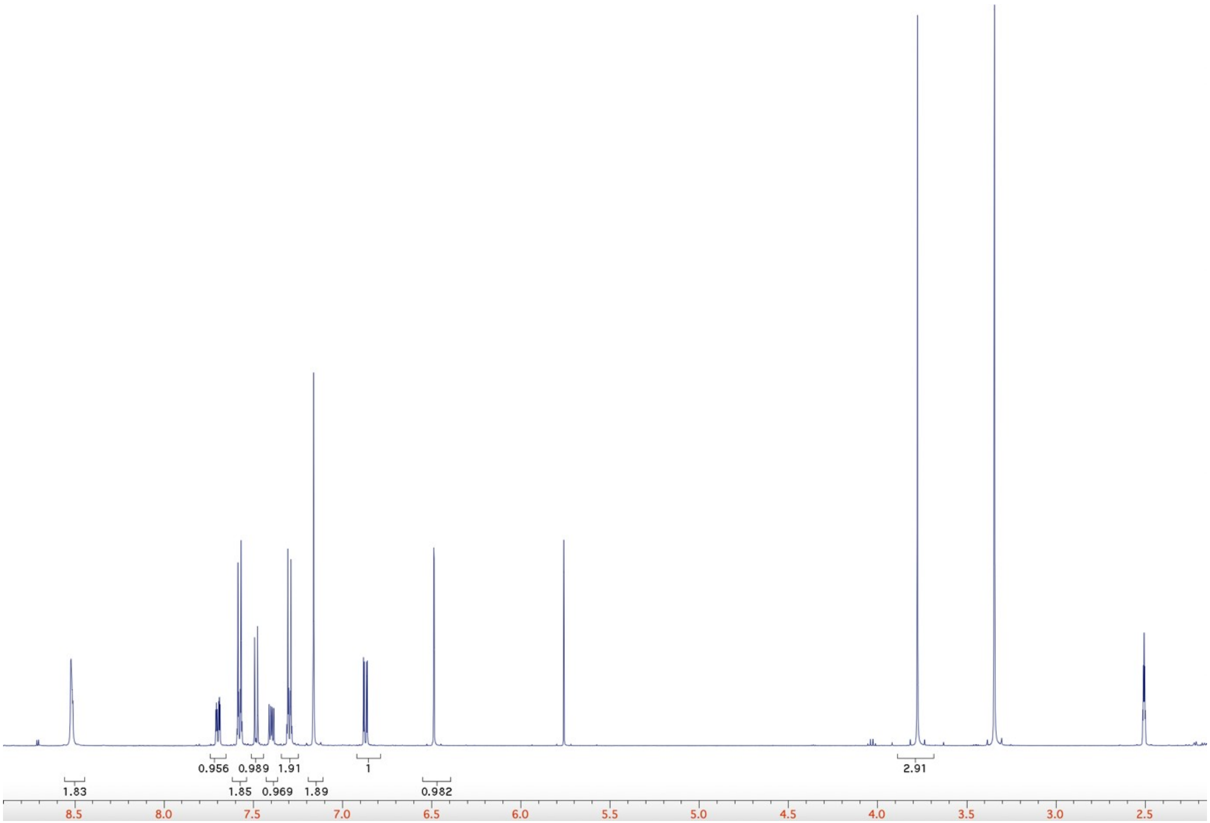

^{13}C NMR:

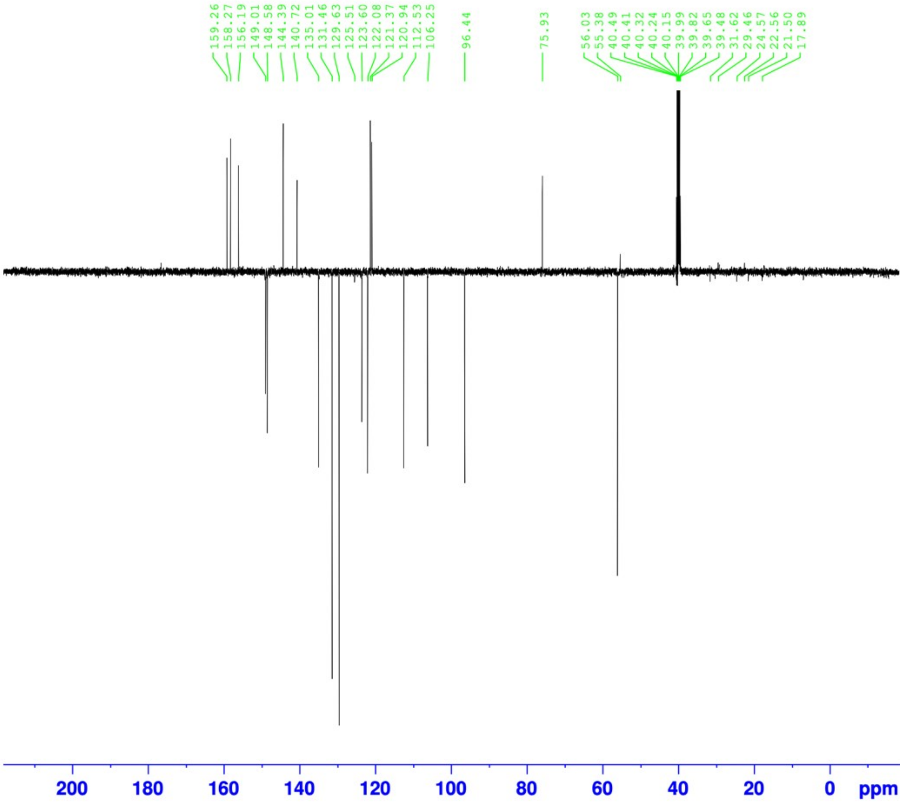

4d ($CDCl_3$)

^{1}H NMR:

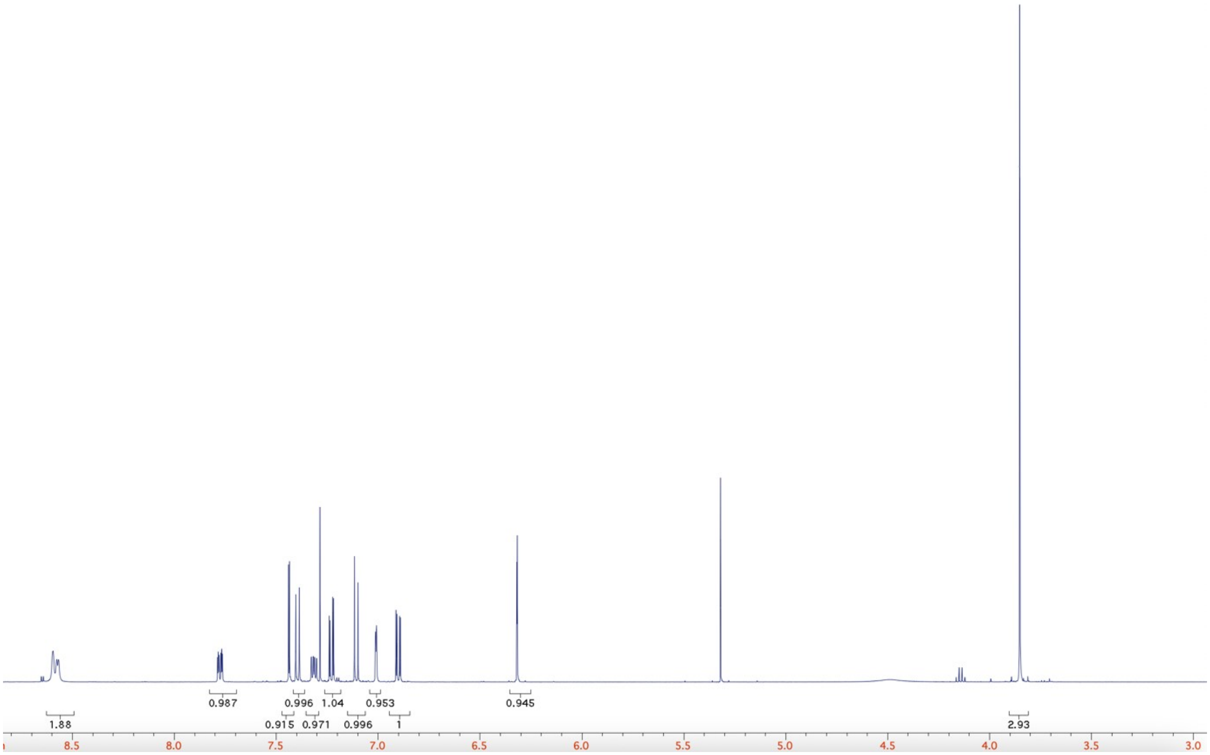

^{13}C NMR:

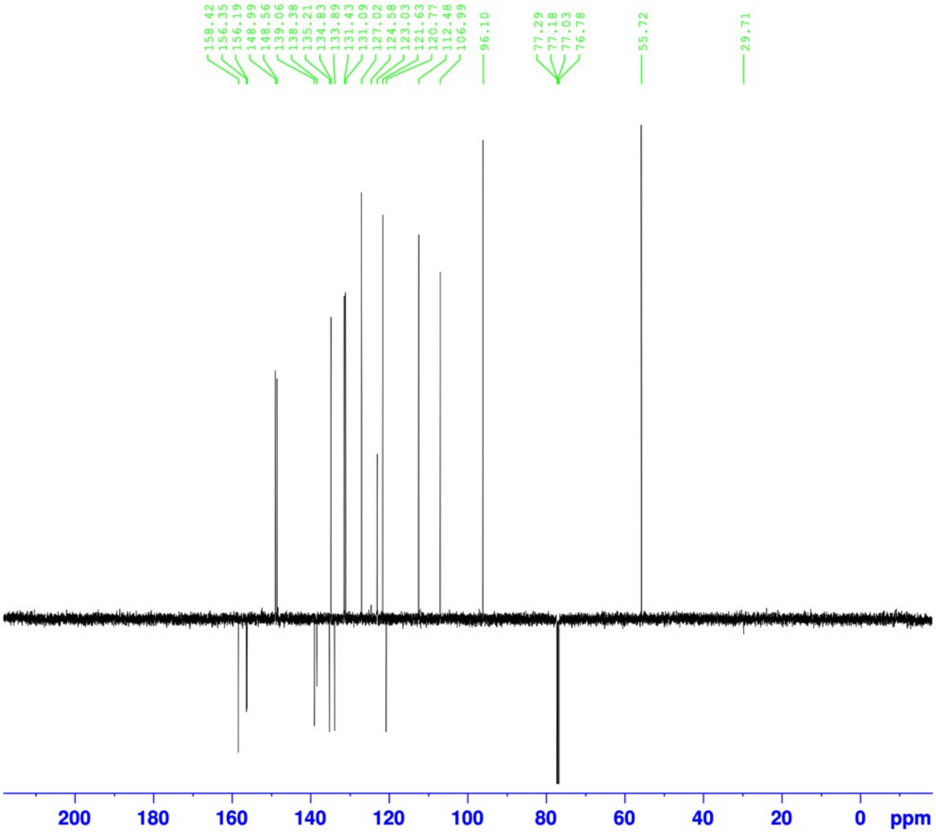

10a ($CDCl_3$)

^{1}H NMR:

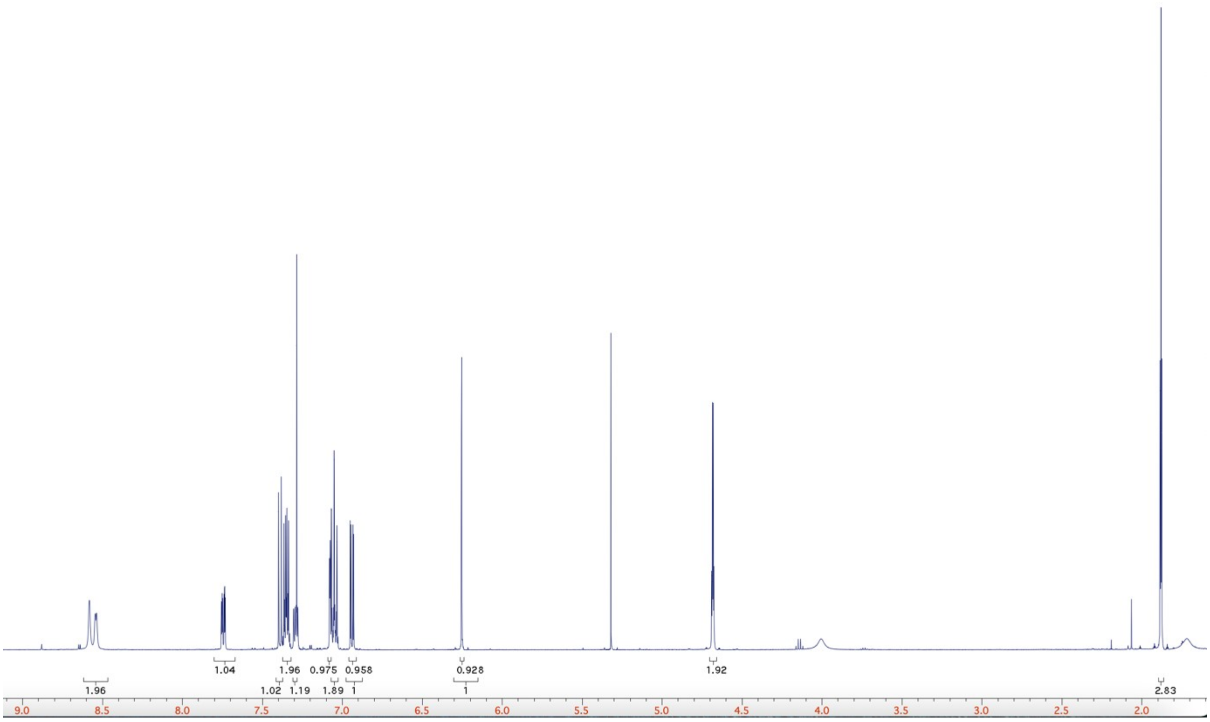

^{13}C NMR:

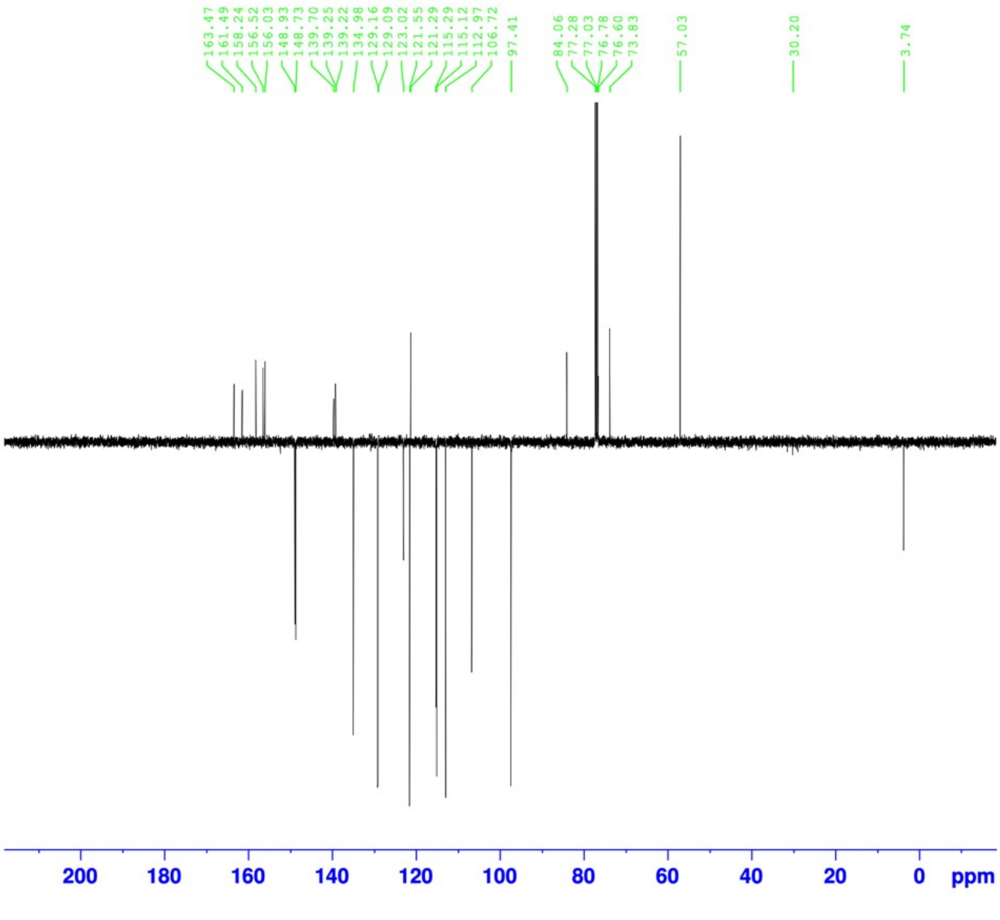

10b ($CDCl_3$)

^1H NMR:

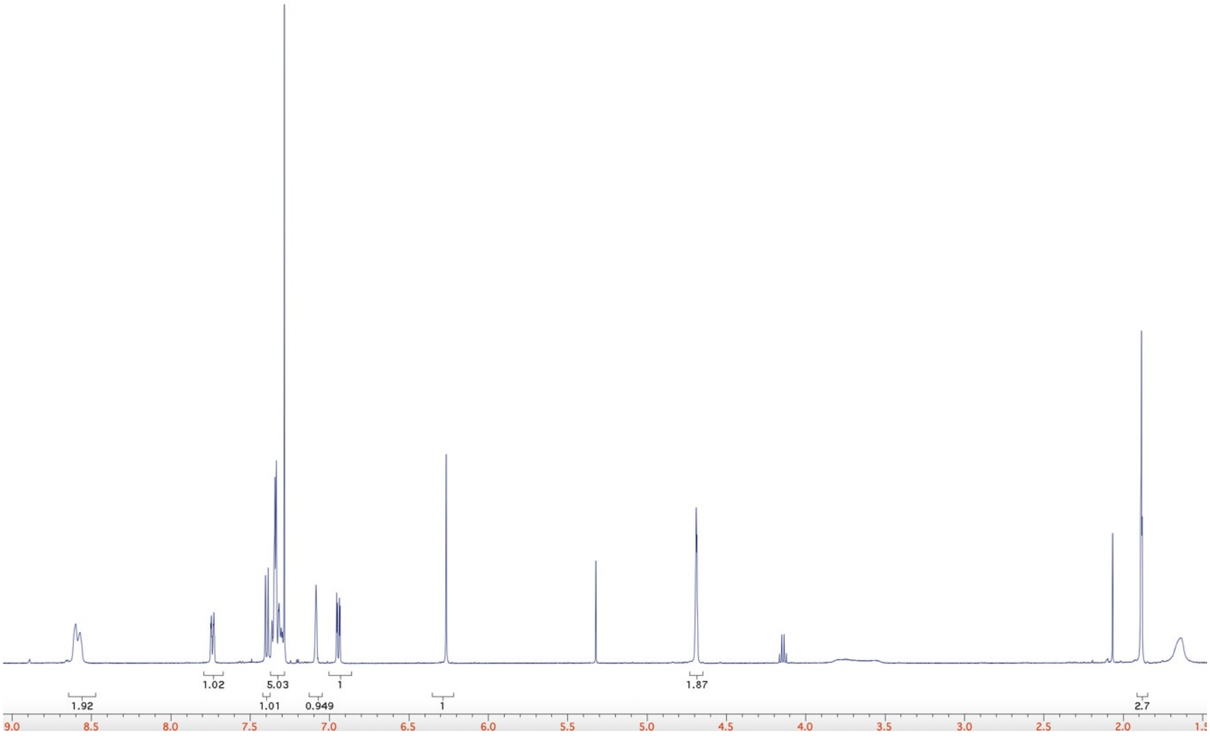

^{13}C NMR:

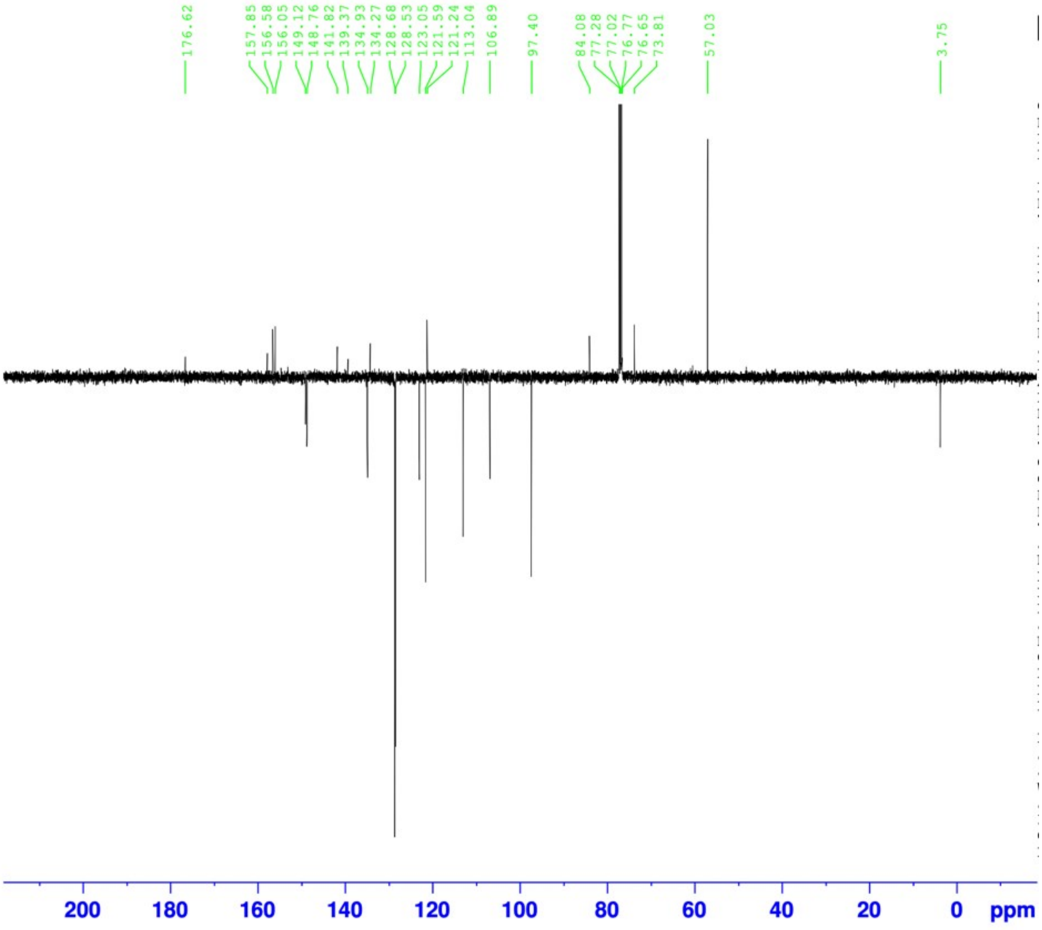

10c ($CDCl_3$)

^1H NMR:

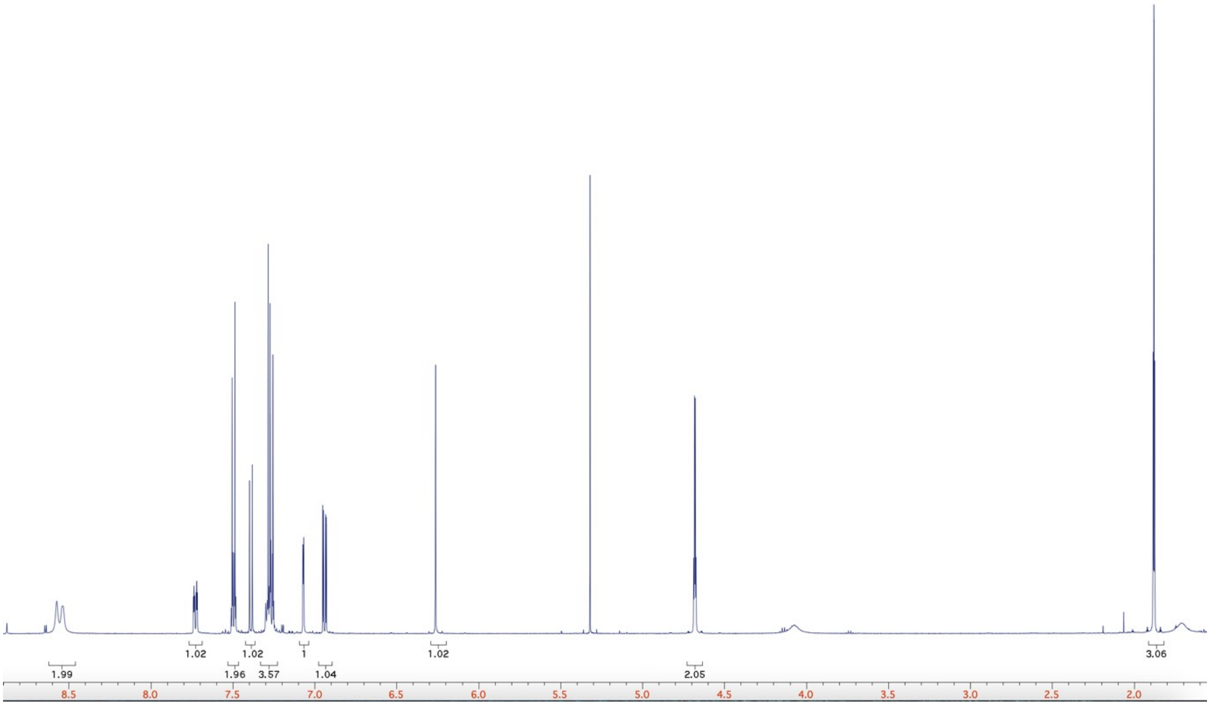

^{13}C NMR:

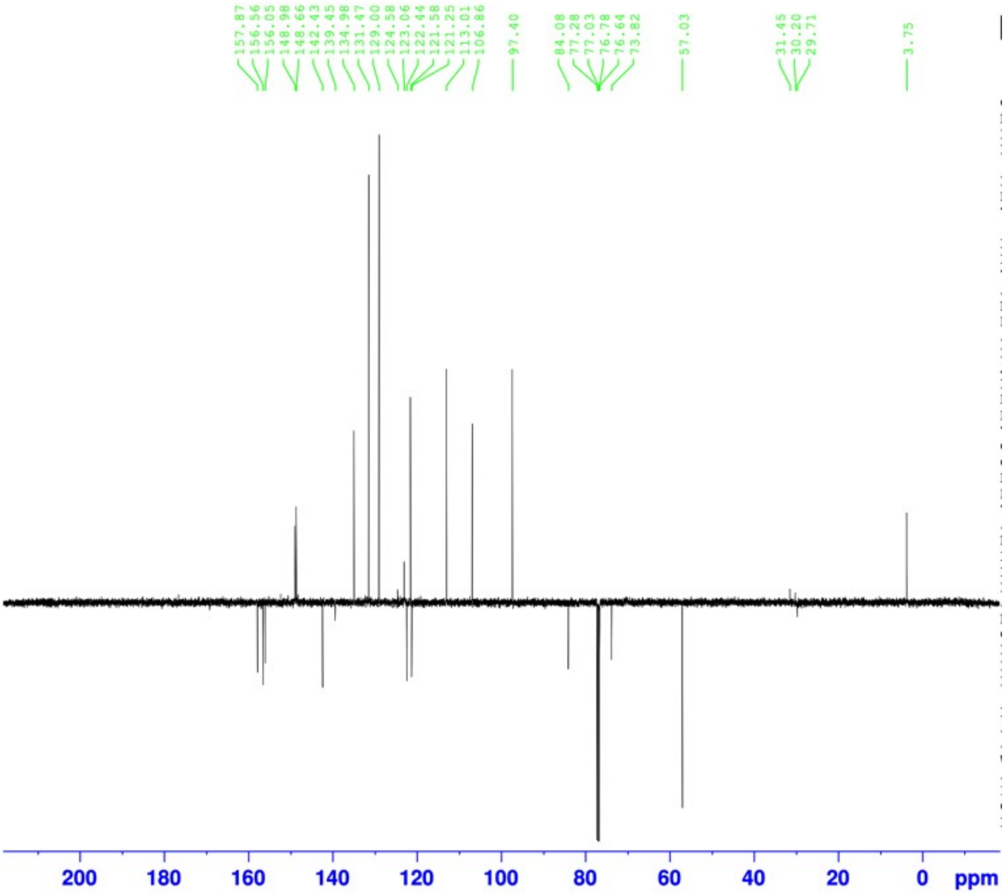

10d ($CDCl_3$)

^{1}H NMR:

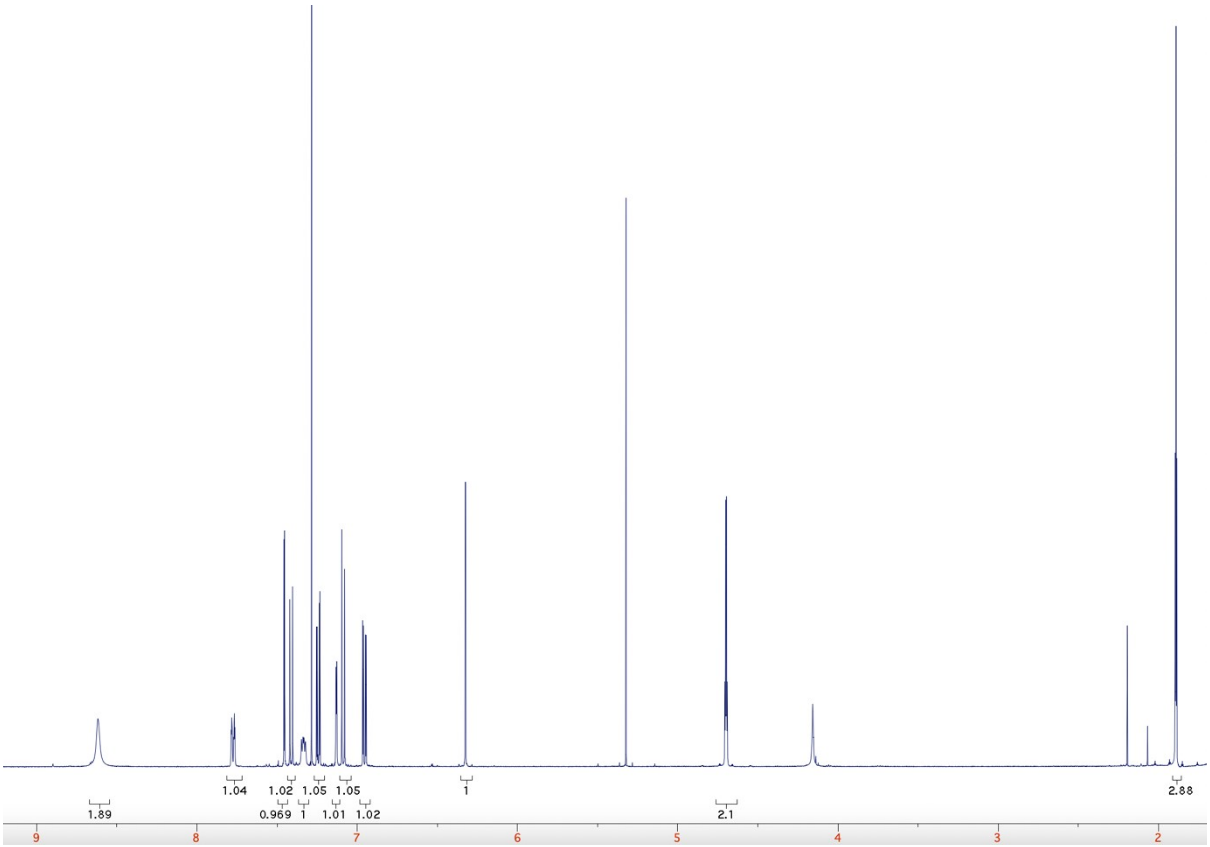

^{13}C NMR:

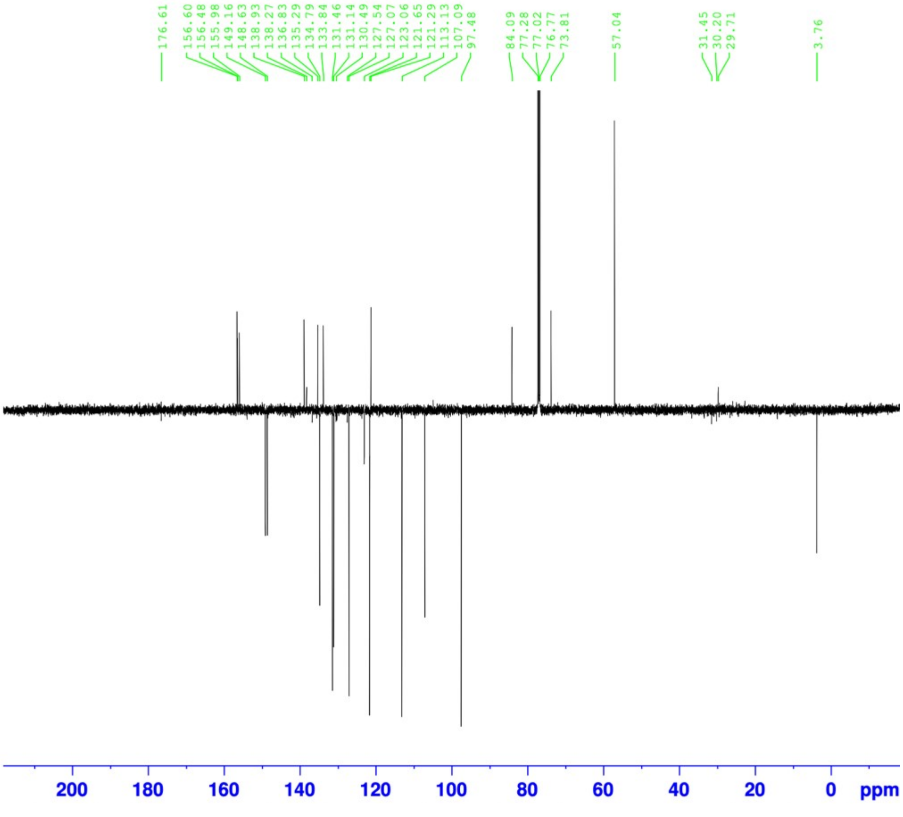

11a ($CDCl_3$)

^{1}H NMR:

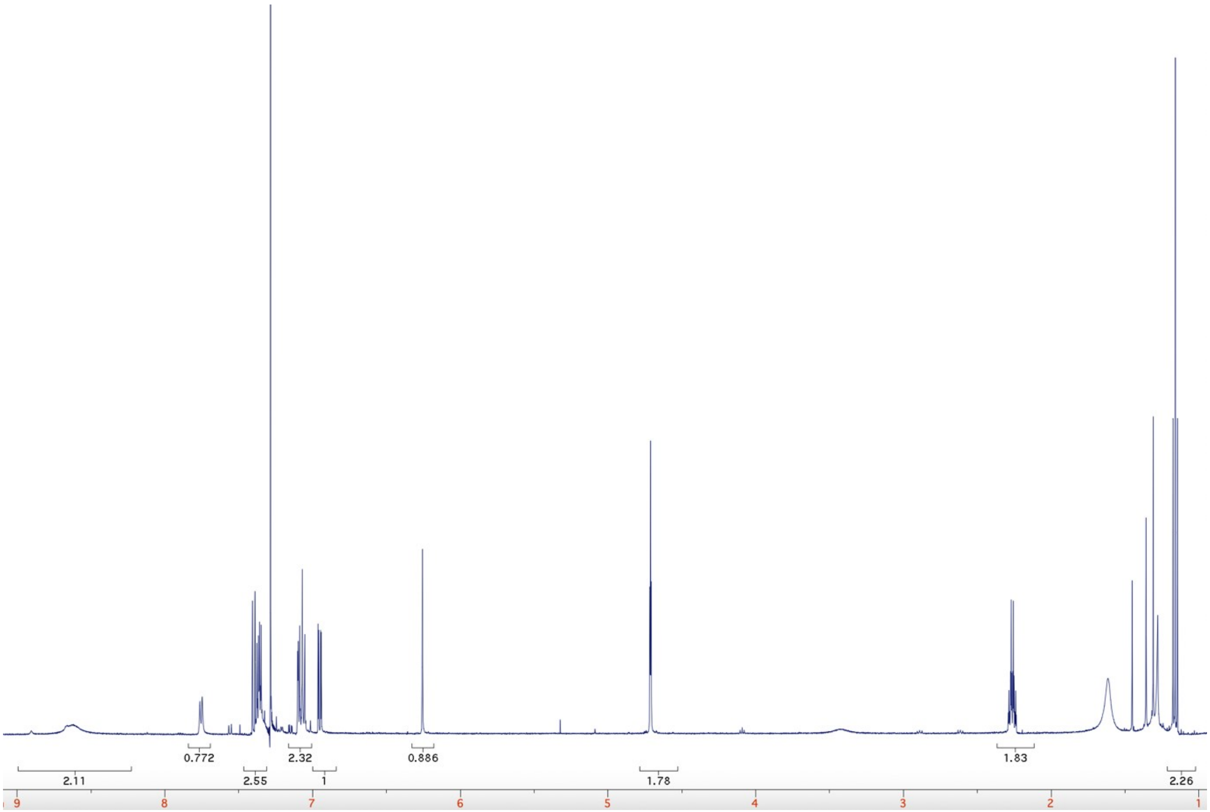

^{13}C NMR:

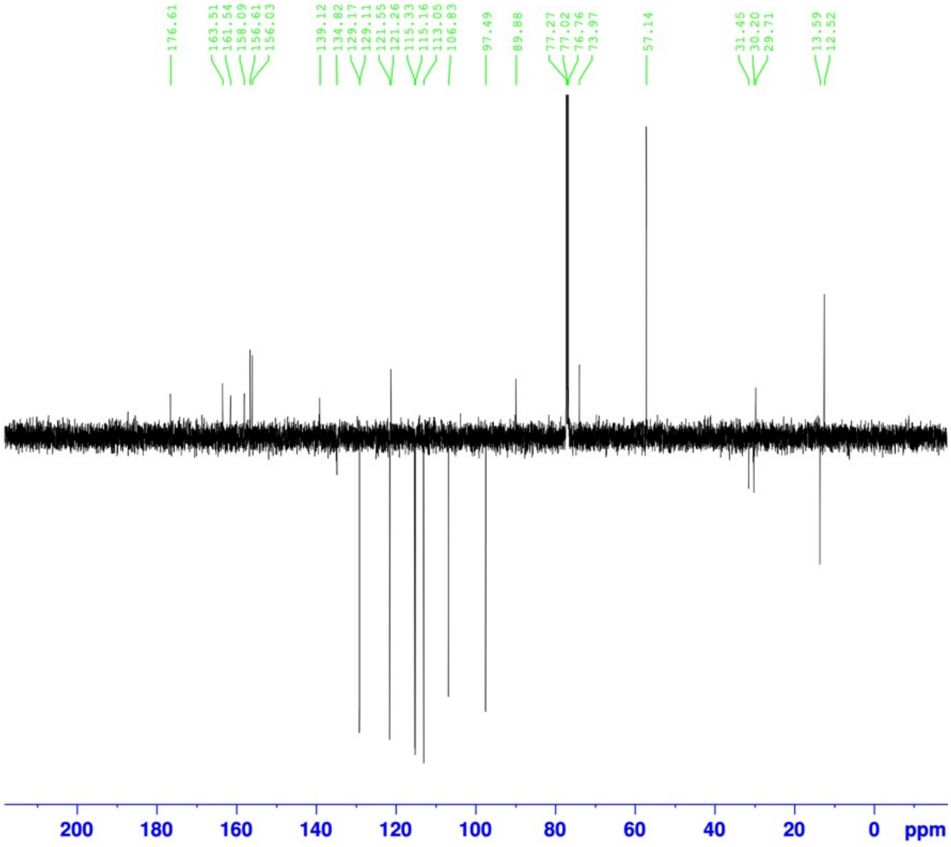

11b ($CDCl_3$)

^{1}H NMR:

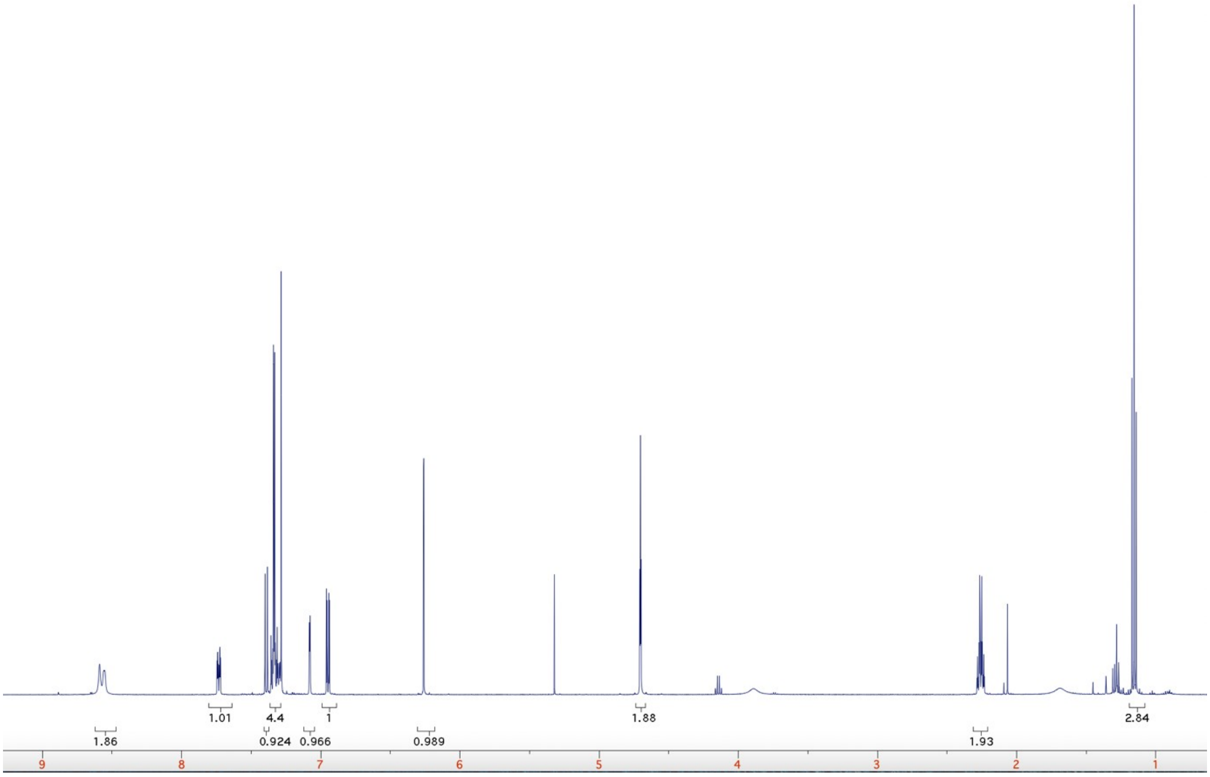

^{13}C NMR:

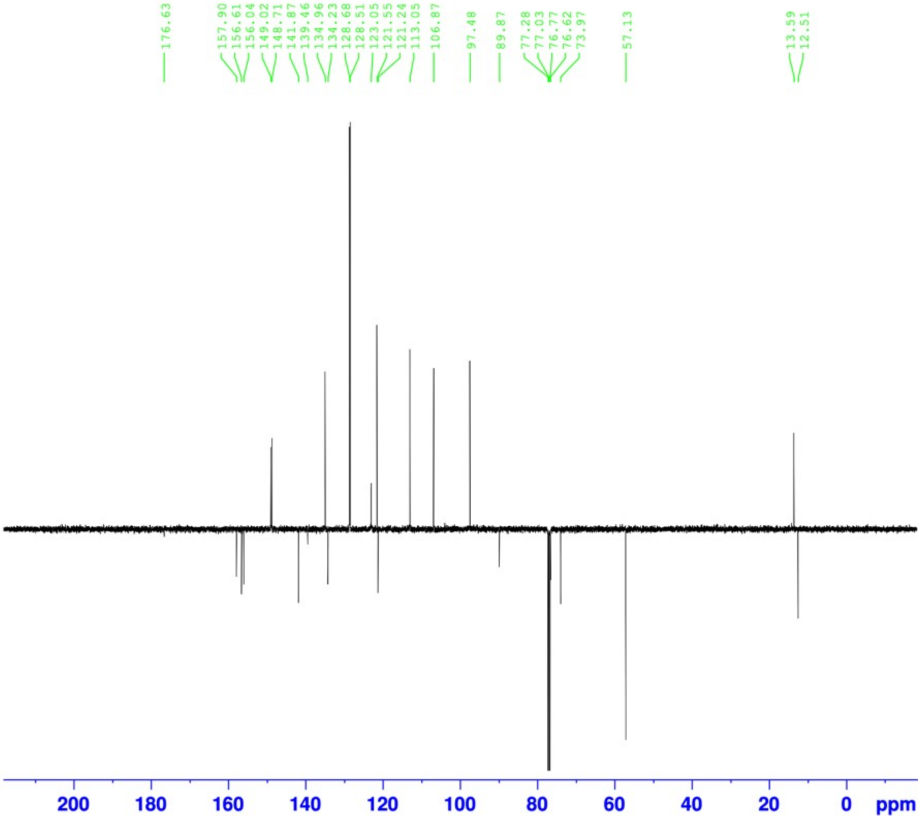

11c ($CDCl_3$)

^1H NMR:

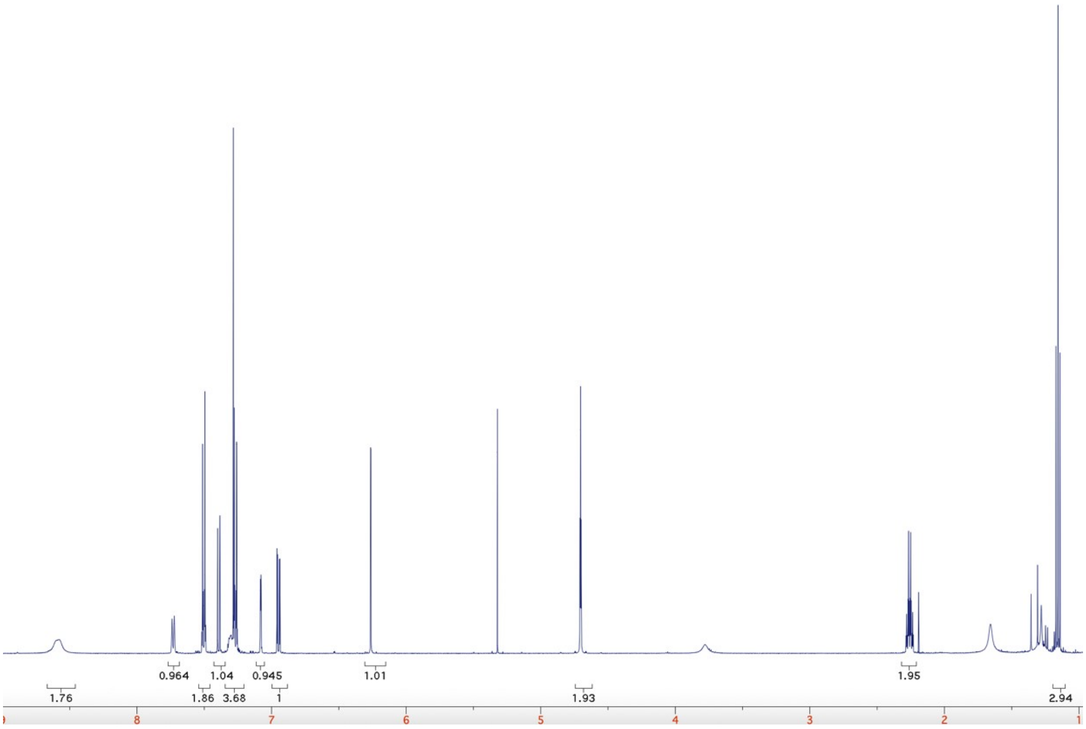

^{13}C NMR:

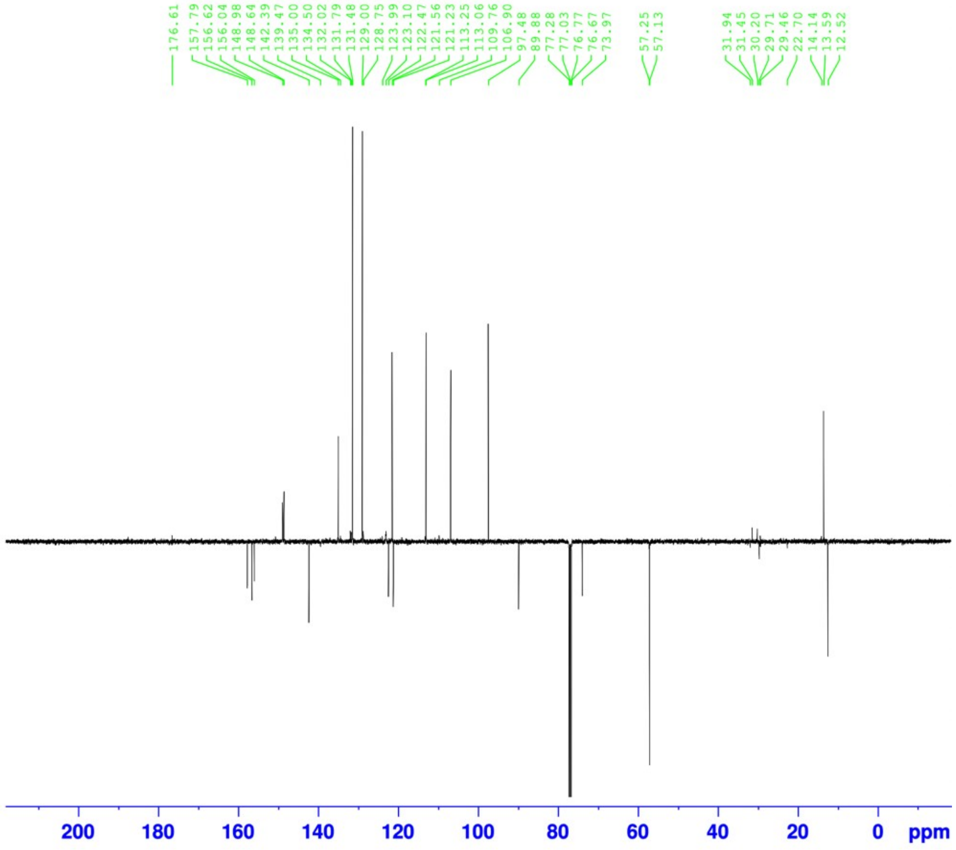

11d ($CDCl_3$)

^1H NMR:

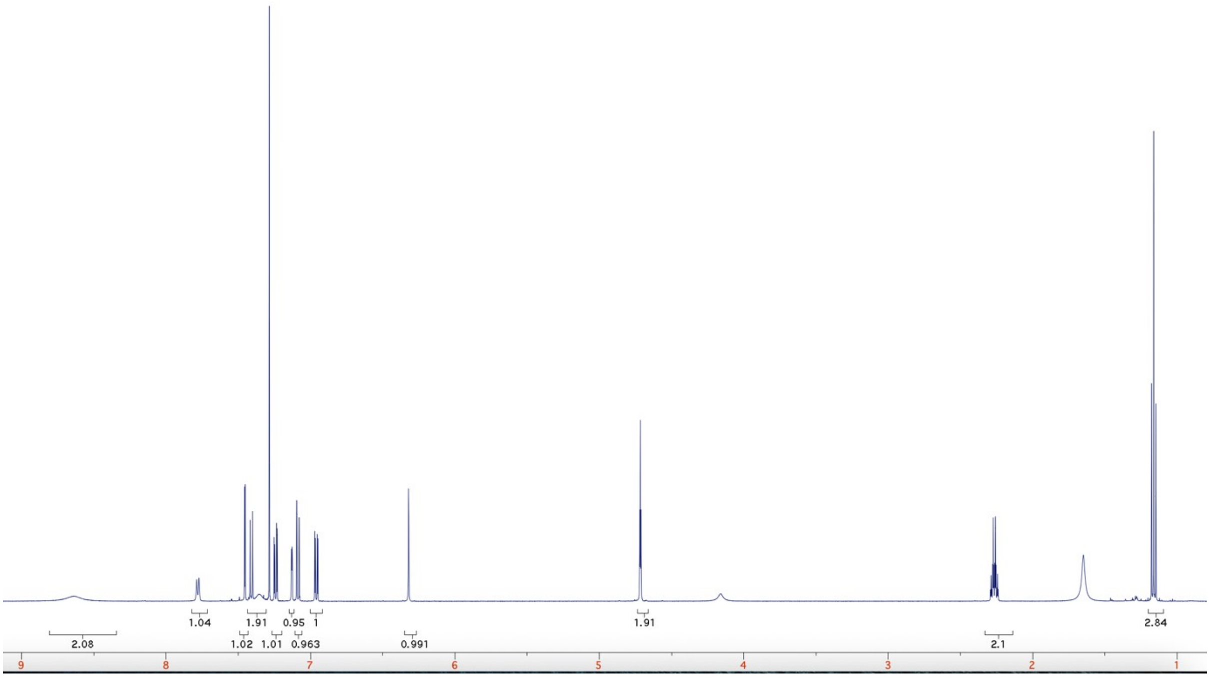

^{13}C NMR:

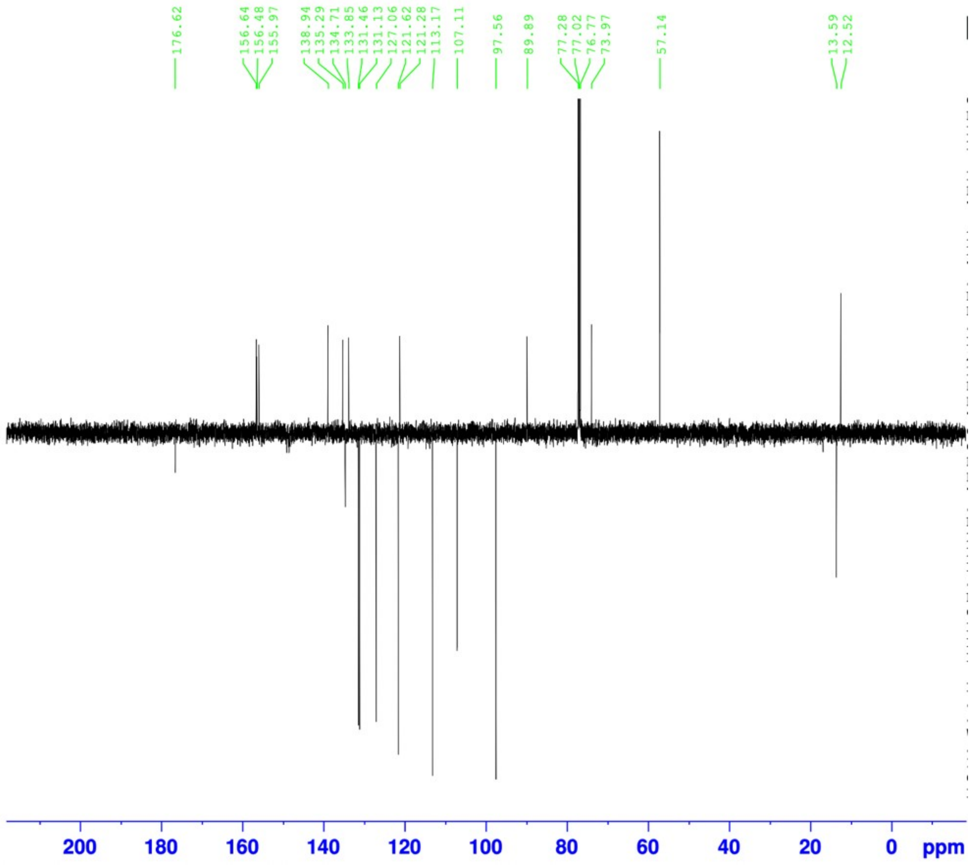

R-**4a**

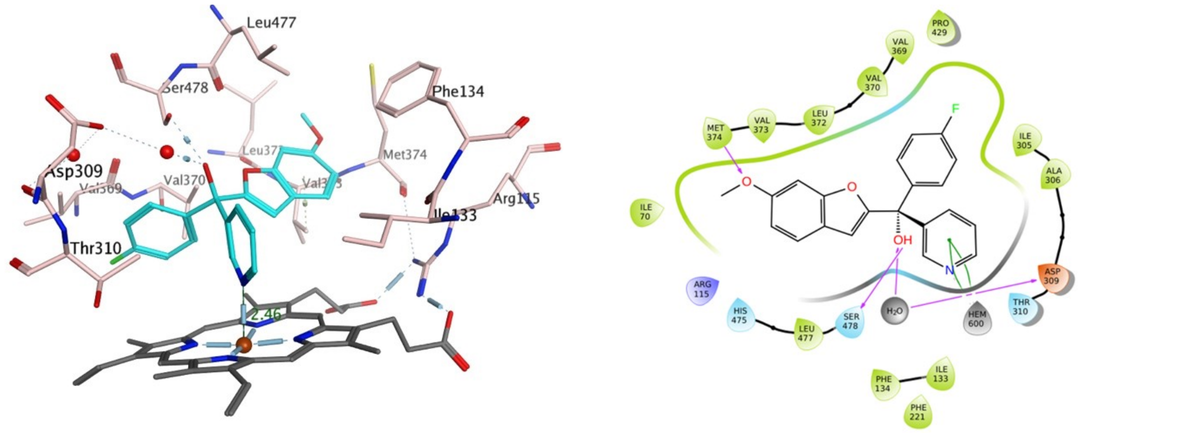

S-**4a**

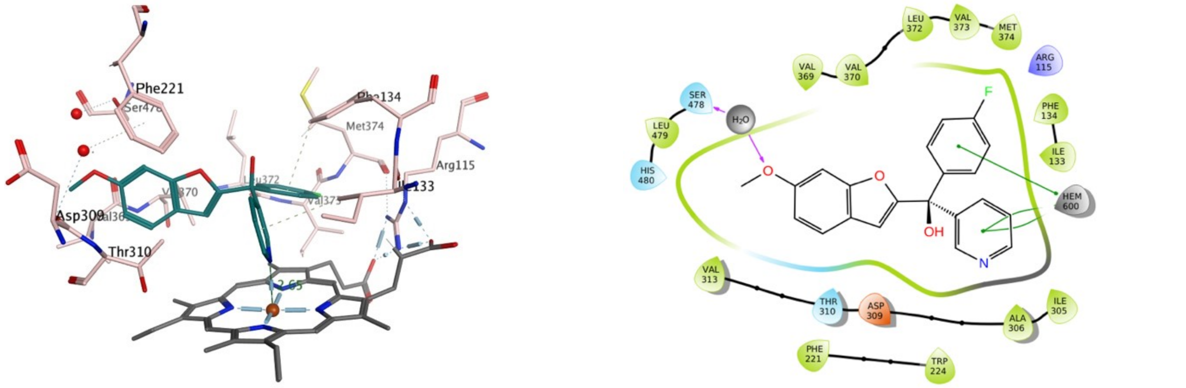

R-**4b**

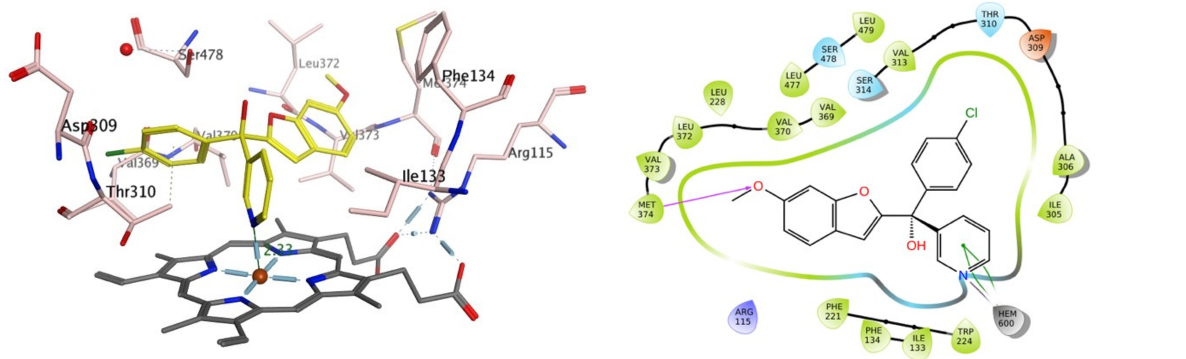

S-**4b**

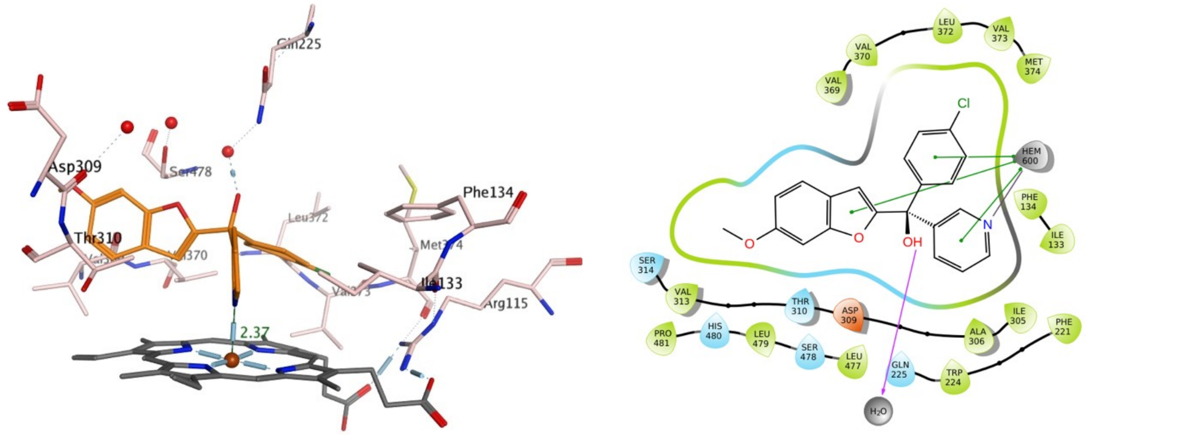

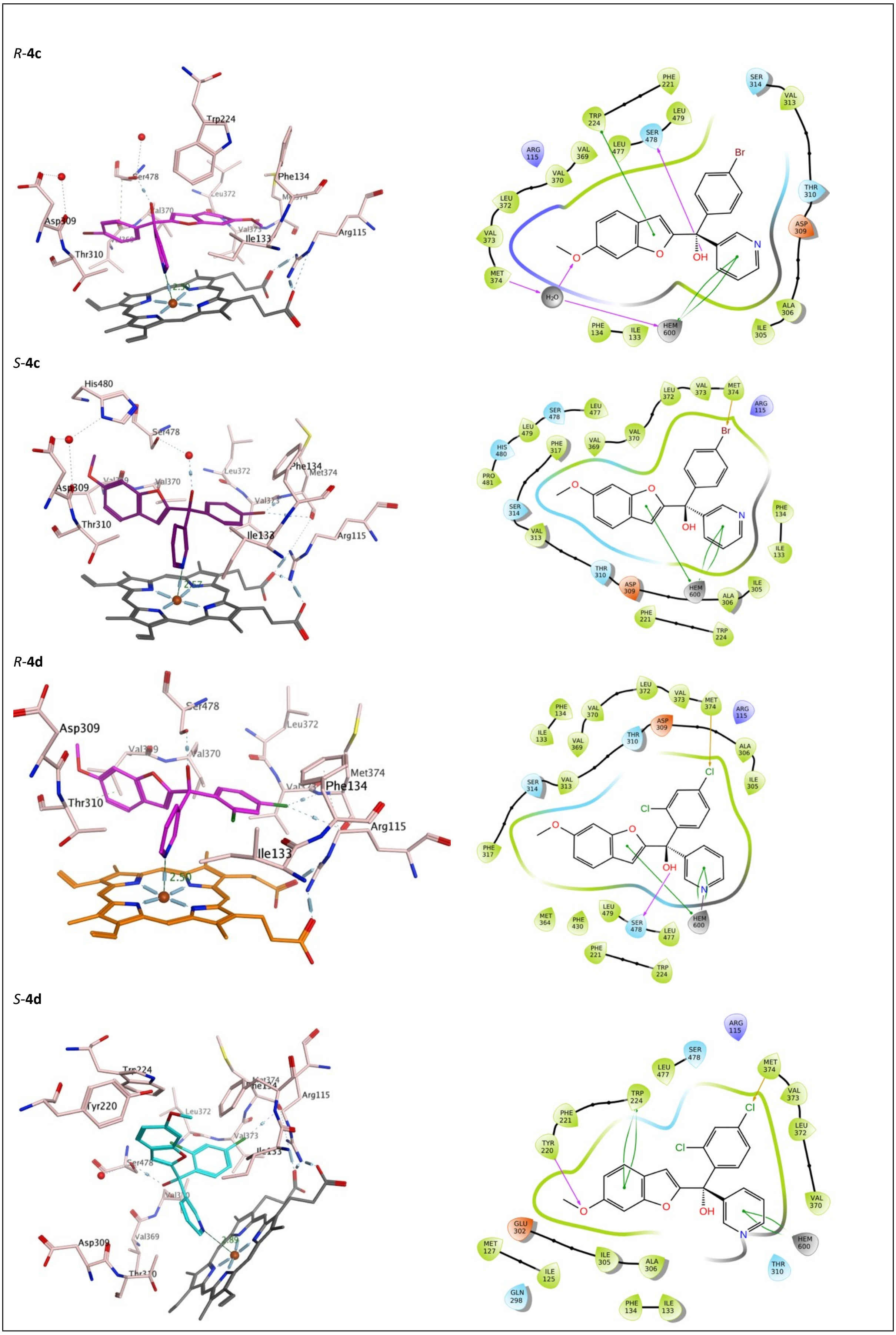

Figure S1. Positioning of the enantiomers of methoxy derivatives (**4**) in the haem active site of CYP19A1 and 2D ligand interaction image of final frame after MD simulation.

R-**10a**

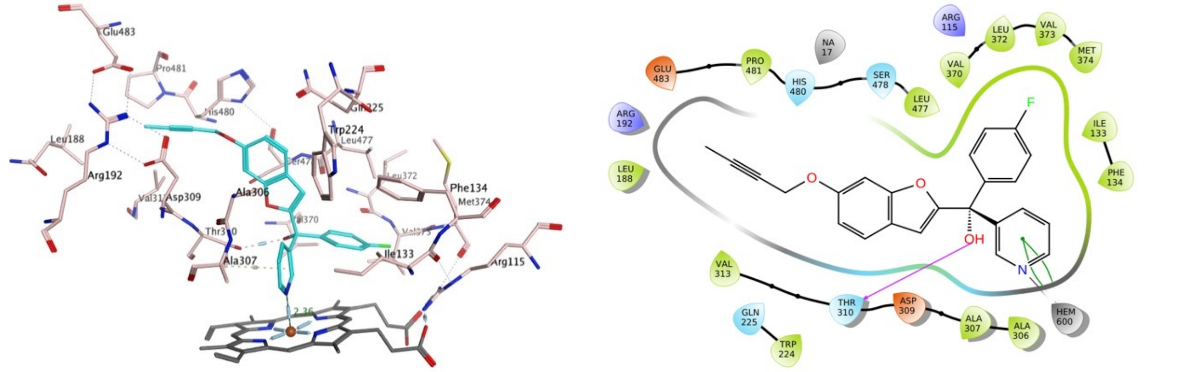

S-**10a**

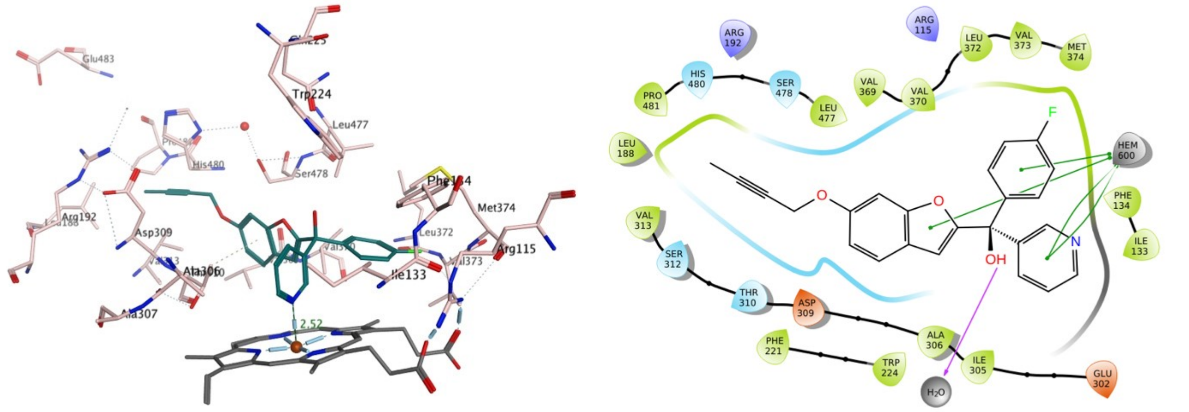

R-**10b**

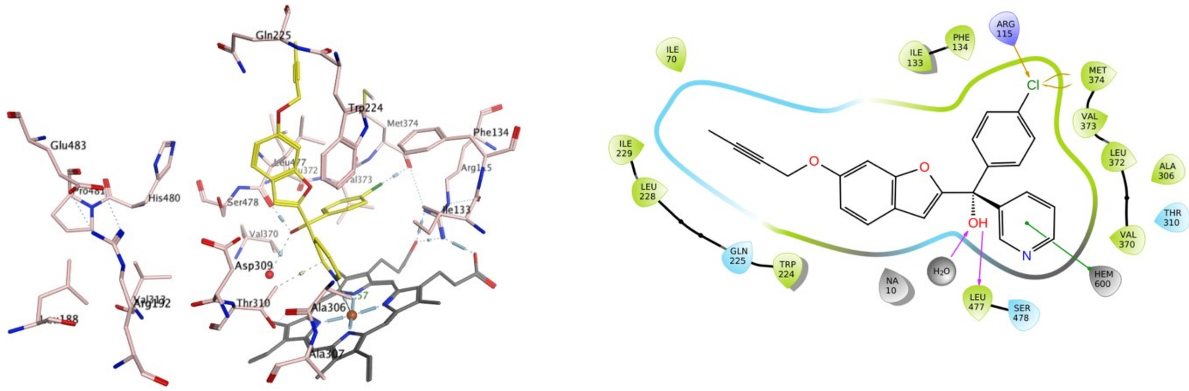

S-**10b**

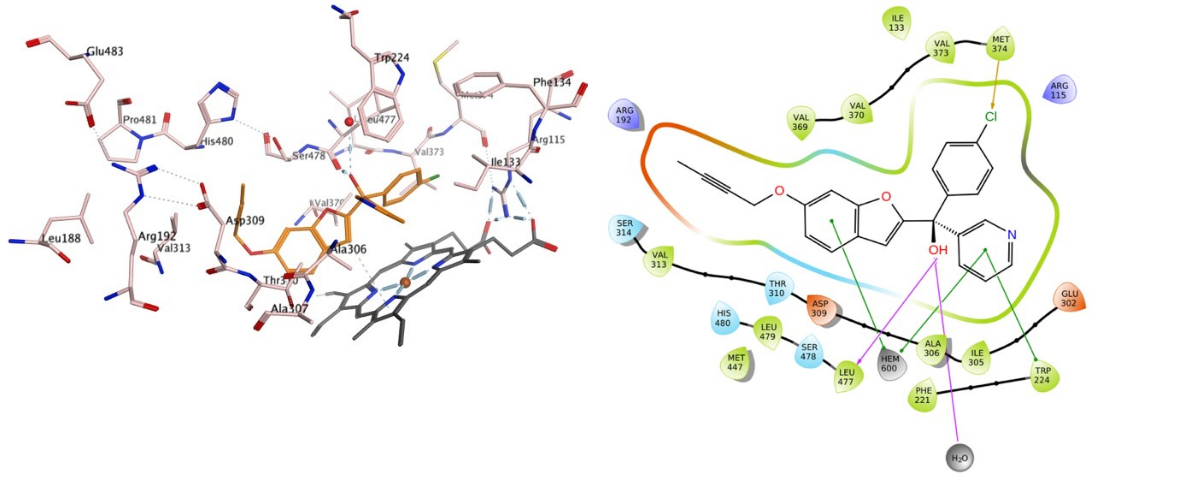

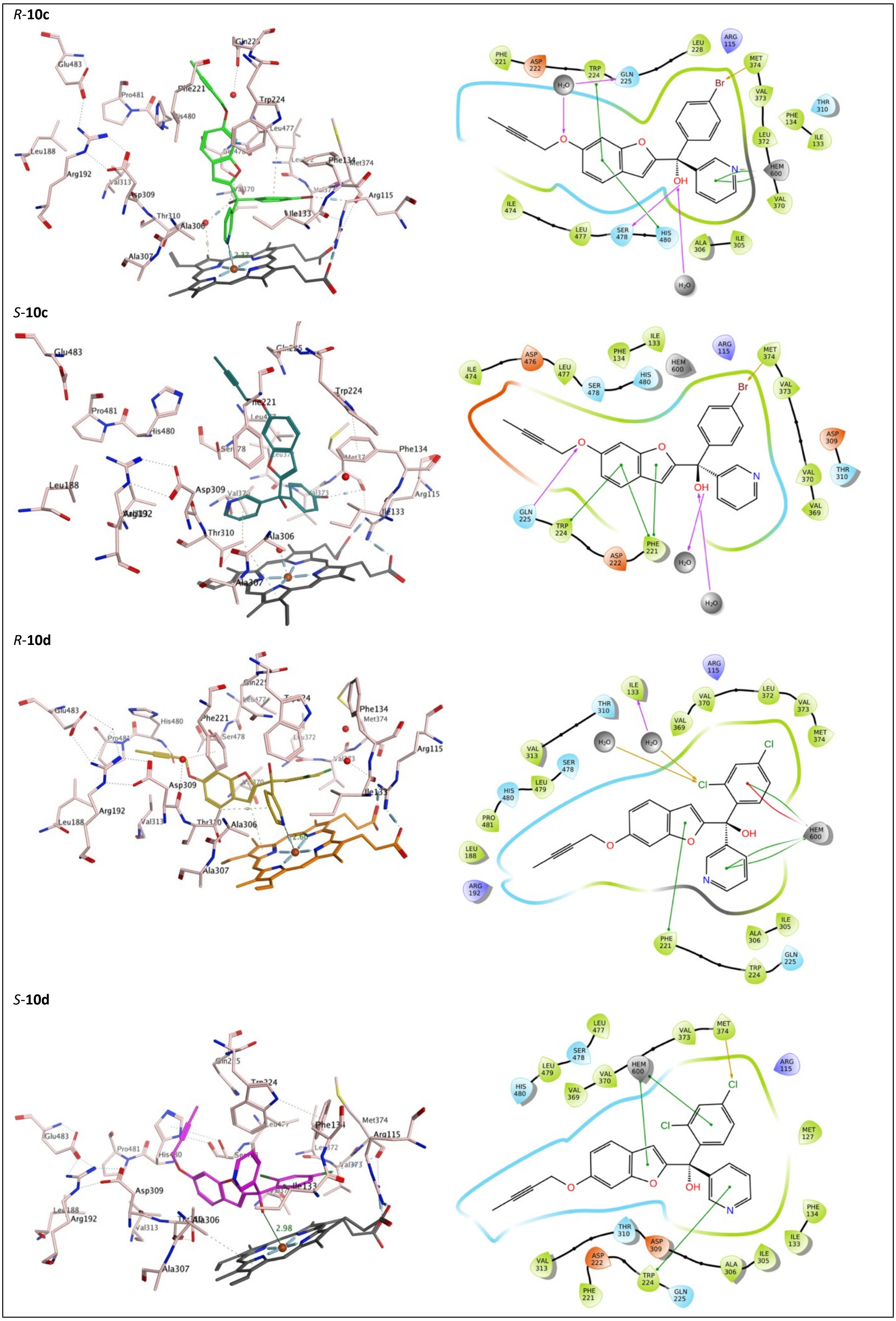

Figure S2. Positioning of the enantiomers of exemplar but-2-ynyloxy derivatives (**10**) in the haem active site of CYP19A1 and access channels and 2D ligand interaction image of final frame after MD simulation. Front door access channel lined by Arg192, Asp309, Pro481, His480 and Glu483 while an alternative access channel is lined by Phe221, Trp224, Gln225 and Leu477.

R-**11a**

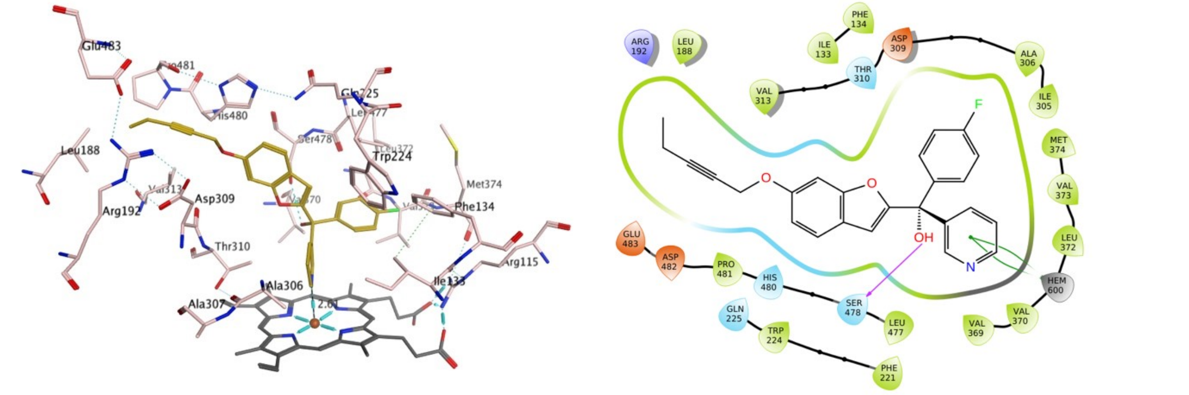

S-**11a**

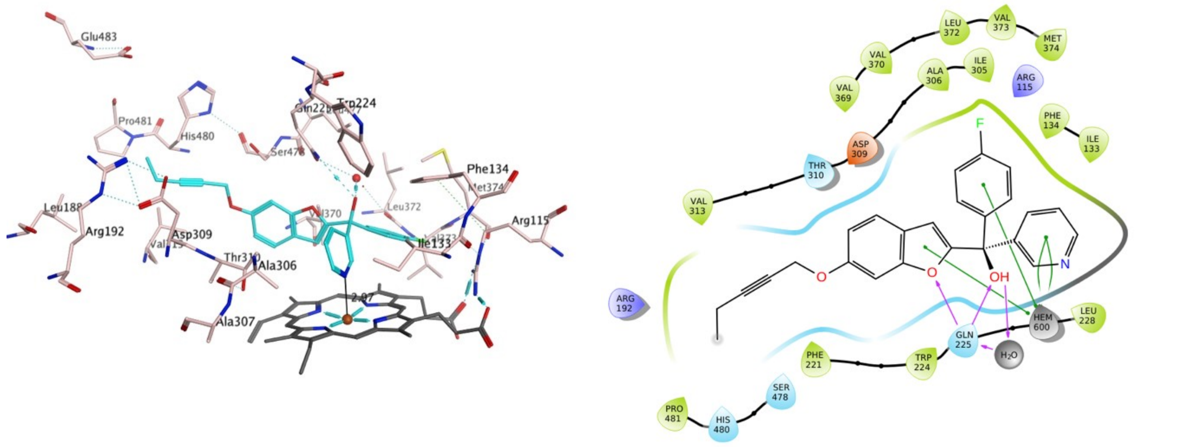

R-**11b**

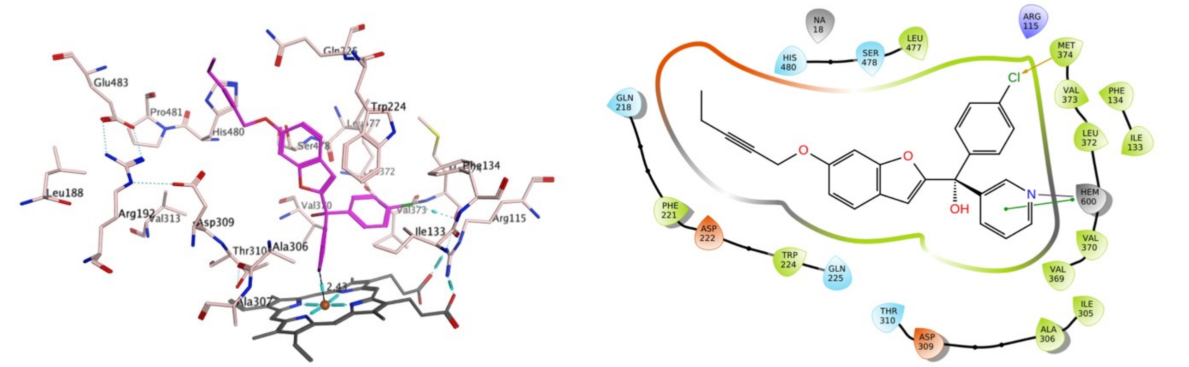

S-**11b**

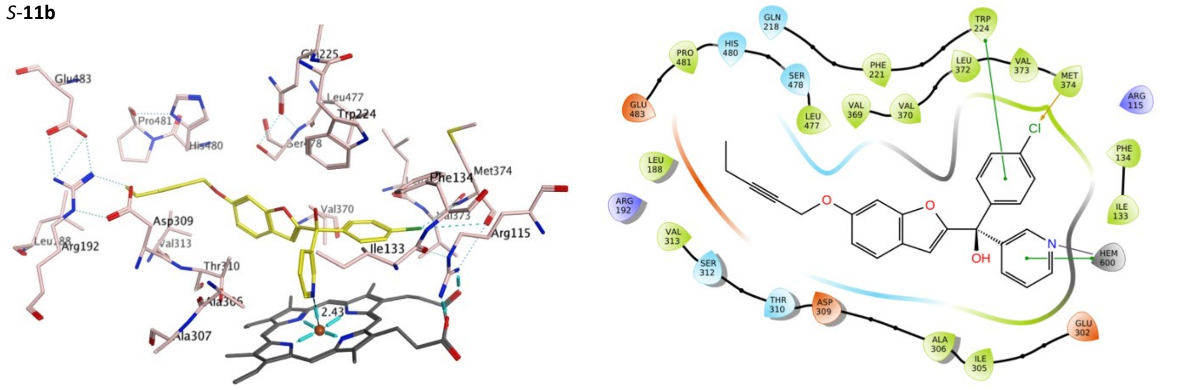

R-**11c**

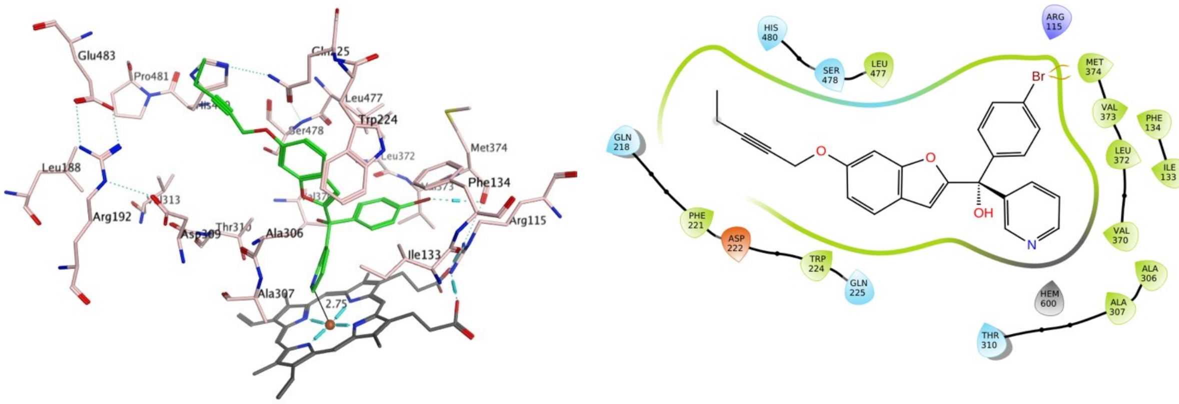

S-**11c**

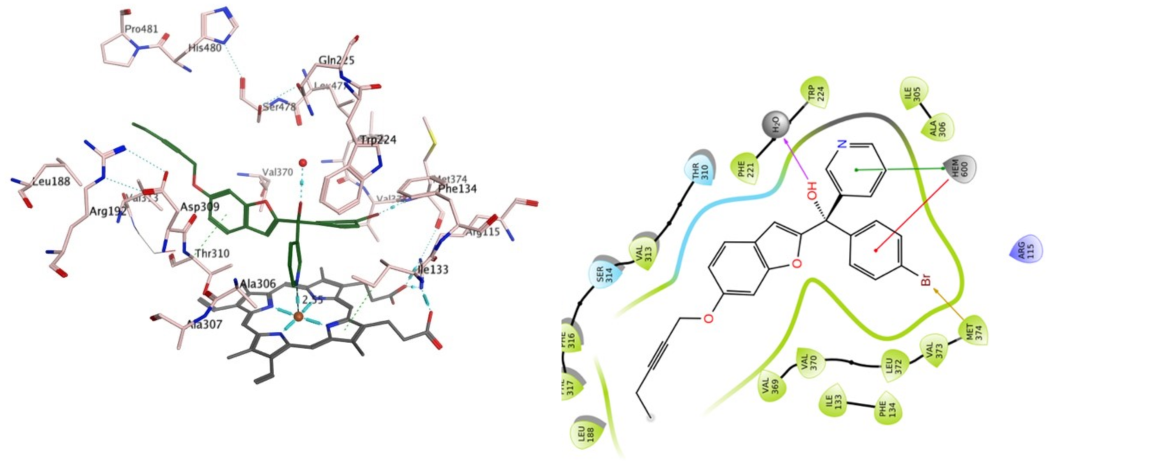

R-**11d**

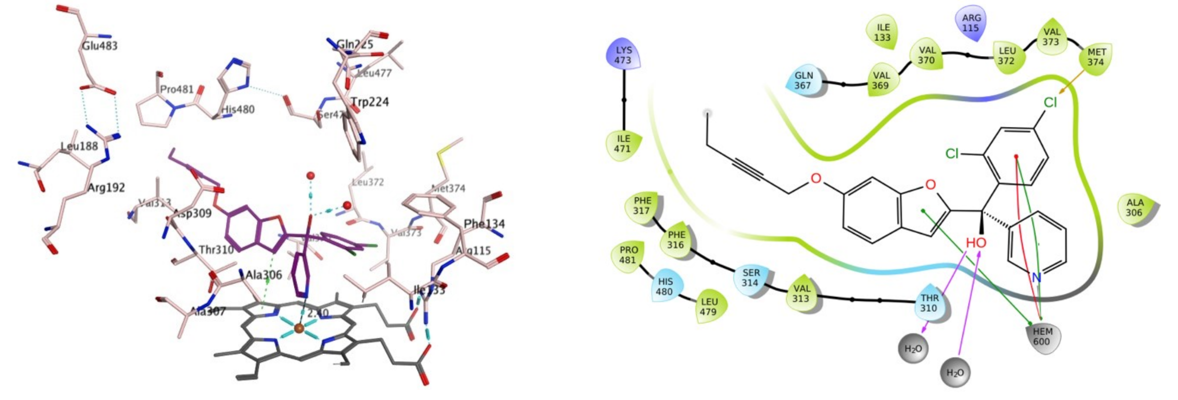

S-**11d**

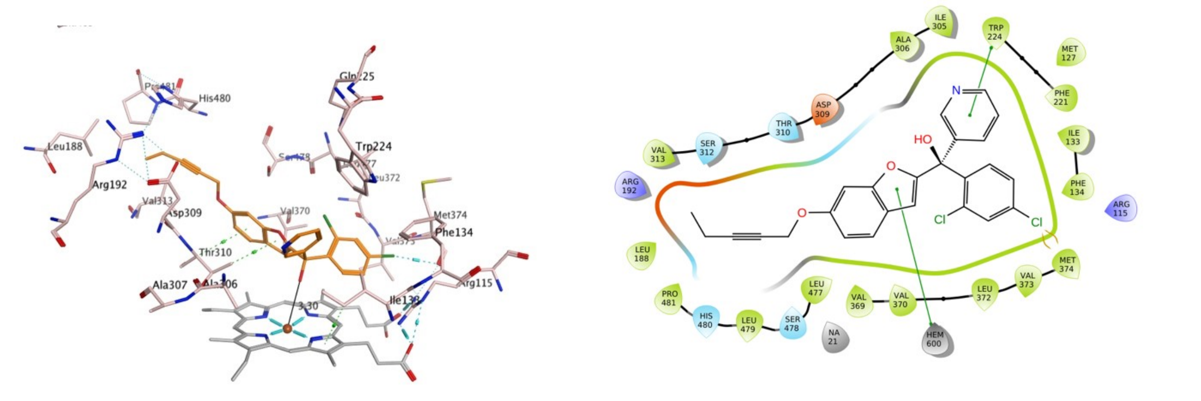

Figure S3. Positioning of the enantiomers of exemplar pent-2-ynyloxy derivatives (**11**) in the haem active site of CYP19A1 and access channels and 2D ligand interaction image of final frame after MD simulation. Front door access channel lined by Arg192, Asp309, Pro481, His480 and Glu483 while an alternative access channel is lined by Phe221, Trp224, Gln225 and Leu477.
